# A Novel *Toxoplasma gondii* Nuclear Factor TgNF3 Is a Dynamic Chromatin-Associated Component, Modulator of Nucleolar Architecture and Parasite Virulence

**DOI:** 10.1371/journal.ppat.1001328

**Published:** 2011-03-31

**Authors:** Alejandro Olguin-Lamas, Edwige Madec, Agnes Hovasse, Elisabeth Werkmeister, Isabelle Callebaut, Christian Slomianny, Stephane Delhaye, Thomas Mouveaux, Christine Schaeffer-Reiss, Alain Van Dorsselaer, Stanislas Tomavo

**Affiliations:** 1 Center for Infection and Immunity of Lille, CNRS UMR 8204, INSERM U 1019, Institut Pasteur de Lille, Université Lille Nord de France, Lille, France; 2 Centre National de la Recherche Scientifique, CNRS UMR 8576, UGSF, Université de Lille 1, Villeneuve d'Ascq, France; 3 Laboratoire de Spectrométrie de Masse Bioorganique, IPHC, CNRS UMR 7178, Université de Strasbourg, Strasbourg, France; 4 Centre National de la Recherche Scientifique, Universités Pierre et Marie Curie-Paris 6 et Denis Diderot-Paris 7, UMR7590, Paris, France; 5 Laboratoire de Physiologie Cellulaire, INSERM U1003, Université de Lille 1, Villeneuve d'Ascq, France; Weill Medical College of Cornell University, United States of America

## Abstract

In *Toxoplasma gondii*, *cis*-acting elements present in promoter sequences of genes that are stage-specifically regulated have been described. However, the nuclear factors that bind to these *cis*-acting elements and regulate promoter activities have not been identified. In the present study, we performed affinity purification, followed by proteomic analysis, to identify nuclear factors that bind to a stage-specific promoter in *T. gondii*. This led to the identification of several nuclear factors in *T. gondii* including a novel factor, designated herein as TgNF3. The N-terminal domain of TgNF3 shares similarities with the N-terminus of yeast nuclear FK506-binding protein (FKBP), known as a histone chaperone regulating gene silencing. Using anti-TgNF3 antibodies, HA-FLAG and YFP-tagged TgNF3, we show that TgNF3 is predominantly a parasite nucleolar, chromatin-associated protein that binds specifically to *T. gondii* gene promoters *in vivo*. Genome-wide analysis using chromatin immunoprecipitation followed by high-throughput sequencing (ChIP-seq) identified promoter occupancies by TgNF3. In addition, TgNF3 has a direct role in transcriptional control of genes involved in parasite metabolism, transcription and translation. The ectopic expression of TgNF3 in the tachyzoites revealed dynamic changes in the size of the nucleolus, leading to a severe attenuation of virulence *in vivo*. We demonstrate that TgNF3 physically interacts with H3, H4 and H2A/H2B assembled into *bona fide* core and nucleosome-associated histones. Furthermore, TgNF3 interacts specifically to histones in the context of stage-specific gene silencing of a promoter that lacks active epigenetic acetylated histone marks. In contrast to virulent tachyzoites, which express the majority of TgNF3 in the nucleolus, the protein is exclusively located in the cytoplasm of the avirulent bradyzoites. We propose a model where TgNF3 acts essentially to coordinate nucleolus and nuclear functions by modulating nucleosome activities during the intracellular proliferation of the virulent tachyzoites of *T. gondii*.

## Author Summary

Apicomplexa including *Toxoplasma gondii* are responsible for a variety of deadly infections. These intracellular parasites have complex life cycles within different hosts and their infectivity relies on their capacity to regulate gene expression in response to different environments. However, to date, little is known about nuclear factors that regulate their gene expression. Here, we have characterized parasite nuclear factors that bind to a stage-specific promoter. We identified several nuclear factors including a novel factor, designated herein as TgNF3. The N-terminal domain of TgNF3 shares similarities with the N-terminus of yeast nuclear FK506-binding protein (FKBP), known as a histone chaperone regulating gene silencing. We show that TgNF3 is predominantly a nucleolar, chromatin-associated protein that specifically binds to *T. gondii* nucleosome-associated histones and promoters. Genome-wide analysis identified promoter occupancies by TgNF3 and we demonstrated a direct role for this factor in transcriptional control of genes involved in parasite metabolism, transcription and translation. Ectopic expression of TgNF3 induces dynamic changes in the size of the nucleolus, and a severe attenuation of parasite virulence *in vivo*. In avirulent bradyzoites, TgNF3 is found exclusively in the cytoplasm, suggesting a potential role in regulating nucleolar and nuclear functions in the virulent tachyzoites of *T. gondii*.

## Introduction

*Toxoplasma gondii* has long been a major medical and veterinary problem capable of causing abortion, or congenital birth defects in both humans and livestock. The advent of AIDS has drawn even more attention to *T. gondii* as a serious opportunistic pathogen. *T. gondii* is distinct from nearly all of the other members of the phylum Apicomplexa, owing to the exceptional range of all warm-blooded animals and humans that serve as hosts. The infection is incurable because of its ability to differentiate from the rapidly replicating tachyzoite stages into latent cysts containing the bradyzoite stages that are impervious to immunity and current drugs. *T. gondii* cysts and dormant bradyzoites persist in the brain of the infected host and also play key roles in pathogenesis because they can convert to virulent tachyzoites in immune compromised individuals with AIDS and in transplant patients. This stage conversion is triggered by the host immune response and impairment of the immune system in HIV infected individuals can lead to lethal toxoplasmic encephalitis.

Although the basal core transcriptional machinery, the protein-coding genes involved in nucleosome assembly and chromatin remodelling machinery were found to be conserved in *T. gondii* genome (http://www.toxodb.org), a surprising finding was the identification of a relatively low number of genes encoding transcription factors in the parasite [1]–[6]. This has led to the proposal that gene regulation in *T. gondii* and other apicomplexan parasites is controlled mainly by epigenetic mechanisms [7]–[9]. However, bioinformatics searches for DNA-binding domains identified, in *Plasmodium spp* and in all apicomplexan parasite genomes sequenced to date, a family of proteins homologous to the plant transcription factor Apetala2, named ApiAP2 for apicomplexan AP2-like factors [10]. De Silva *et al.* have demonstrated the DNA-binding specificities of two ApiAP2 proteins that have a high specificity for unique DNA sequence motifs found in the upstream regions of distinct sets of genes co-regulated during asexual development [11]. One *Plasmodium* ApiAP2 factor has a major role in stage-specific gene regulation by activating a set of genes, including genes reported to be required for midgut invasion. It has also been described that this ApiAP2 factor binds to specific six-base sequences in the proximal promoters [12]. Our current knowledge from *T. gondii* transcriptome indicates that mRNA pools are dynamic and transcriptional control is also a primary means to regulate the developmental transitions of the parasites, suggesting that gene regulation occurs mostly at the transcriptional level [13], [14]. Microarray studies have demonstrated that transcriptional regulation required timed expression of clusters of genes during the bradyzoite development and that for most genes changes in transcription are tied to modulations in protein expression [15]–[18]. Further confirmatory data is provided by the Serial Analysis Gene Expression (SAGE), which supports the notion that transcriptional regulation plays a key role in the developmental program of *T. gondii* [19]. We and others have previously established that *T. gondii* stage conversion is accompanied by the expression of a variety of genes that displayed diverse functions, suggesting that parasite differentiation is clearly regulated in part at the transcriptional level [20]–[28]. In addition, several promoter sequences have been characterized in *T. gondii* [29]–[31]. We have shown that the promoter regions of two stage-specifically expressed genes displayed promoter autonomy that can be exploited to achieve developmental expression of reporter genes [31]. Yet almost nothing is known about the nature of nuclear factors that can specifically bind to *T. gondii* promoters and regulate transcriptional activity.

Here, we report the isolation and characterization of a novel *T. gondii* promoter-specific binding factor designated herein as TgNF3, which shares similarities with yeast nuclear FK506-binding protein (FKBP), known to be a histone chaperone regulating rDNA silencing. We demonstrate that TgNF3 protein is predominantly a nucleolar, chromatin-associated protein that binds specifically to *T. gondii* gene promoters *in vivo*, leading to a direct role in transcriptional regulation. ChIP-seq and genome-wide analysis of TgNF3 targets identified gene promoters mainly involved in parasite metabolism, transcription and translation. Importantly, TgNF3 interacts directly to core and nucleosome-associated histones in the context of gene silencing. Furthermore, we show that TgNF3 is a dynamic chromatin-associated factor, a modulator of nucleolus biogenesis, parasite replication and virulence. Taken together, our findings suggest a major role of TgNF3 in nucleosome activity that may regulate nucleolar and nuclear functions during the intracellular proliferation of *T. gondii*.

## Results

### Isolation of nuclear factors that bind to *T. gondii* promoter

**Figure 1:**
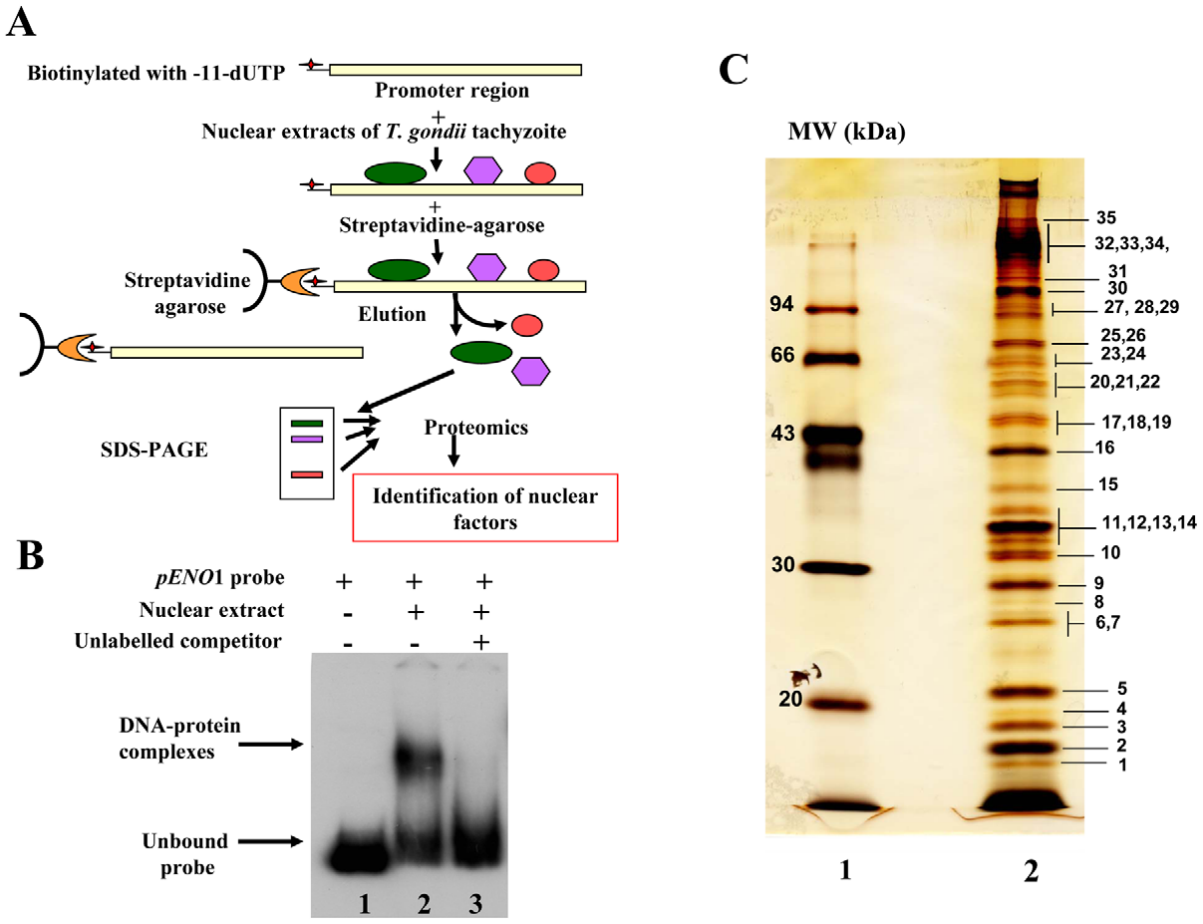
Affinity purification of *T. gondii* nuclear factors. A). A schematic diagram showing the experimental strategy devised for the purification of ENO1 promoter binding proteins. B) Electrophoretic mobility shift assays (EMSA) showing DNA-protein complexes using the biotinylated DNA sequence corresponding to the ENO1 promoter and total nuclear extract of tachyzoites. Lane 1, unbound biotinylated probe alone. Lane 2, gel shift binding assays revealing the biotinylated DNA-protein complexes. Lane 3, specific competitor corresponding to unlabeled ENO1 promoter introduced simultaneously with labelled probe during binding assays. C) SDS-PAGE and silver staining after a large scale affinity purification of ENO1 probe bound proteins using a total nuclear extract from *T. gondii* tachyzoites. The markers (MW) indicated in kilodaltons (kDa) are shown on the left.

To test the suitability of using a stage-specific promoter to purify and determine the identity of nuclear factors that interact and control the activity of a *T. gondii* stage-specific promoter, we performed affinity chromatography using biotinylated DNA sequence (Figure S1) from the previously reported bradyzoite-specific *ENO1* promoter [31] used as bait (experimental strategy outlined in Figure 1A). After biotinylation, we checked whether the probe still binds to parasite nuclear factors. As shown in Figure 1B, the biotinylated probe strongly interacts specifically with nuclear factors in gel retardation. The specificity of the DNA-protein complexes visualized (lane 2) was demonstrated by a competition assay using unlabelled probe (lane 3), confirming the presence of bound parasite nuclear factors. To determine the nature of these nuclear factors, large-scale affinity purification was carried out using the biotinylated bait incubated with a nuclear extract containing about 17 mg of total nuclear proteins obtained from 4×10^10^ tachyzoites. Thirty-five nuclear proteins were identified and isolated after SDS-PAGE and silver staining (Figure 1C). These proteins were excised as gel slices and subjected to proteomics analyses. Using mass spectrometry and database searches, we identified thirty nine putative nuclear factors, which are presented as three groups in Supplementary Table 1 (Table S1): 1) A class of 11 proteins, which displayed significant similarities to known nuclear factors. These include a protein possessing a RNA-specific DEAD/DEAH box helicase domain (genbank identifier (gi) number 211966692) and a protein having a pinin domain (genbank identifier (gi) number 211966969). Members of the pinin family have various localisations (including nuclear location) within eukaryotic cells and are thought to regulate protein-protein interactions [32]. A protein (gi number 211962881), which shares similarities with a nuclear FK506-binding protein (*Spodoptera frugiperda* FKBP46), is also found in this group. The FKBP46 homologue in *Saccharomyces pombe*, SpFKBP39, was reported to be involved in transcription repression of ribosomal DNA [33]. Other nucleolar factors and nuclear proteins containing DNA-binding motifs were also present. 2) A second class of 7 proteins corresponds to kinases, phosphatases and heat shock proteins. 3) Finally, 21 hypothetical proteins, more than half of the total number of factors discovered, displayed no obvious similarity to known factors. However, our further bioinformatics analyses identify two proteins in this group (genbank identification (gi) numbers 211967631 and 211968320 in Table S1) as homologues of Alba, ancient archaeal chromatin-associated factors. The Alba factors are known to be involved in gene silencing operating through chromatin regulation in Archaea [34], [35]. Several proteins identified during the proteomics analyses could clearly be expected to be present in the nucleus of the parasite, for example those with strong similarities to known nucleolar factors. However, the majority of enzymes and hypothetical proteins identified could not be obviously considered as genuine parasite nuclear factors. Therefore, we decided to verify whether some of these factors are truly localized in the nucleus of *T. gondii*.

### Nuclear localization of candidate factors identified by proteomics analyses

**Figure 2:**
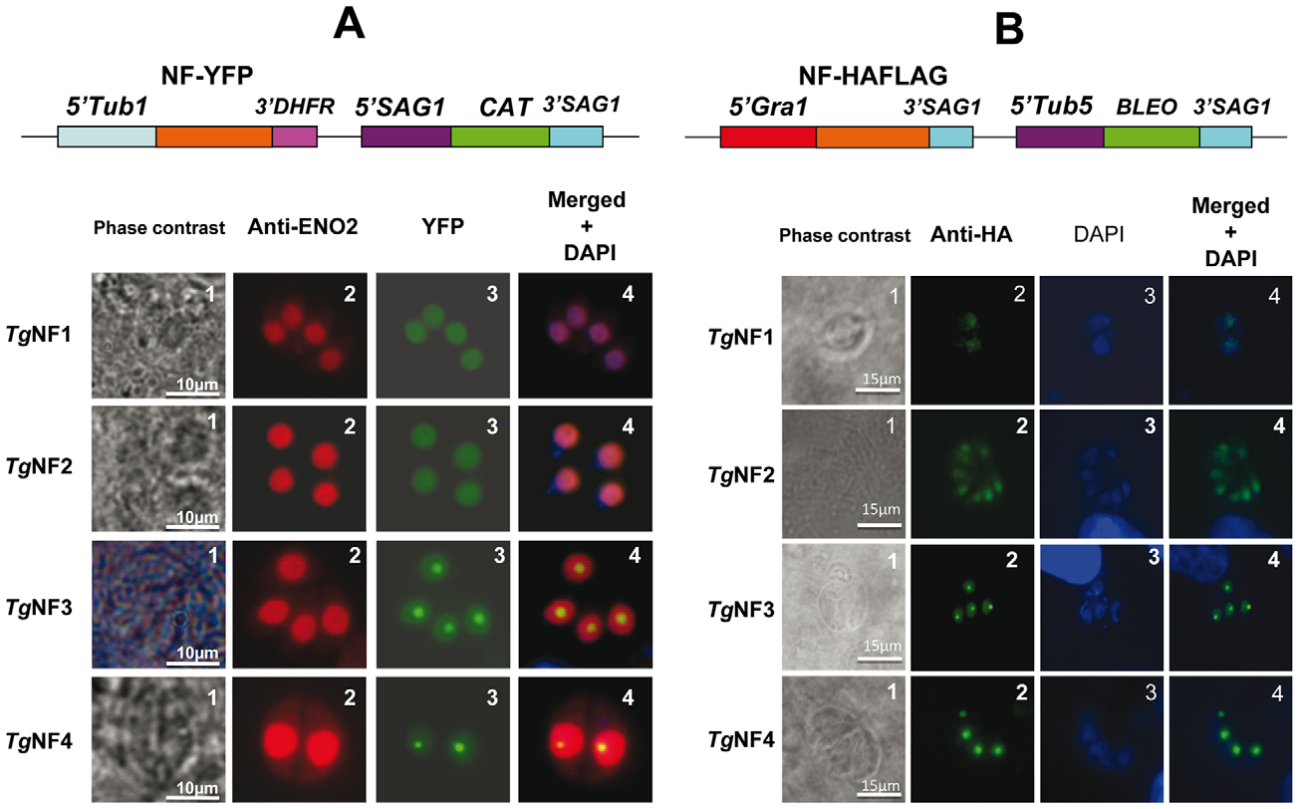
Four chosen candidate factors binding to ENO1 promoter are targeted into *T. gondii* nucleus after transient transfection. A) The full-length cDNA of 4 candidate factors designated TgNF1, TgNF2, TgNF3 and TgNF4 were PCR amplified and cloned in frame to YFP. B) The full-length cDNA of TgNF1, TgNF2, TgNF3 and TgNF4 were also tagged to HAFLAG. The two different vectors are schematically presented on the top of each panel. The expression of these candidate factors was driven either by heterologous tubulin or dense granule 1 gene promoters. These two *T. gondii* vectors also contain a chloramphenicol acetyltransferase (*CAT*) or bleomycin (*Bleo*) cassette whose expression is driven by surface antigen 1 (SAG1) or tubulin gene promoters. Direct fluorescence was detected for YFP expression (panels A) whereas indirect fluorescence assay (IFA) was performed using monoclonal antibody specific to the HA tag (panels B). The nuclei of intracellular tachyzoites were stained with the polyclonal antibodies specific to ENO2 or with a classical nuclear dye (DAPI), both known to label the whole parasite nuclear space except the area defining the parasite nucleolus. The phase contrast image corresponding to TgNF3 shown on left of panel A was captured using a blue filter whereas other images were from a classical filter. The scale bars are shown on these figures.

**Figure 3:**
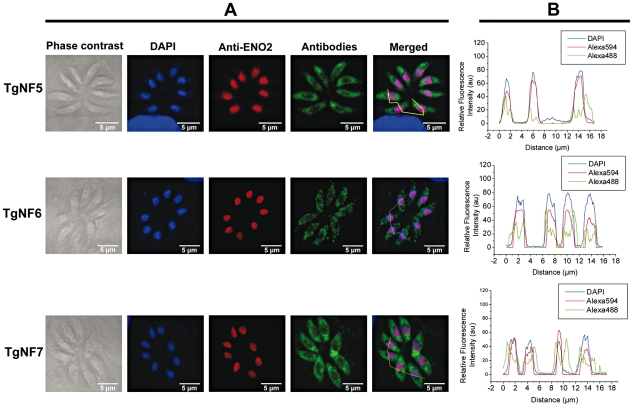
Three other candidate factors have a dual cytoplasm/nuclear localization. The full-length cDNA of 3 candidate factors designated TgNF5, TgNF6 and TgNF7 were PCR amplified and cloned in frame to GST. Purified recombinant proteins were used to generate polyclonal antibodies in mice. A) Indirect immunofluorescence assays using anti-TgNF5, anti-TgNF6 and anti-TgNF7 (green). The nuclei of intracellular tachyzoites were stained with the polyclonal antibodies specific to ENO2 (red) or with DAPI (blue). The scale bars are shown on these figures. B) DAPI (blue), ENO2 (red) and TgNF5, TgNF6 and TgNF7 (green) fluorescence intensities were measured and their respective profiles were superimposed. The quantitative levels of DAPI and fluorescence signals in Panel B relate to the cross sections of intracellular tachyzoites, referenced by the jagged lines in the merged images of Panel A.

We have chosen 7 candidate proteins and used two distinct tagged constructs to examine the presence of these factors in the parasite's nucleus. The full-length cDNAs of these candidate proteins were fused to YFP or HAFLAG tags and their expression was driven by tubulin (Tub 1) and dense granule 1 (GRA1) promoters, respectively. We compared the location of YFP tagged proteins relative to ENO2, a glycolytic enzyme known to be predominantly detected in the nucleoplasm but not in the nucleolus of active replicated intracellular tachyzoites [36]. Figure 2A illustrates four YFP-tagged candidate proteins with convincing nuclear location assessed by direct fluorescence microscopy. These candidate proteins were therefore named TgNFs for *T. gondii* nuclear factors. TgNF1 (gi number 211966692) and TgNF2 (gi number 211966969), containing a DEAD/DEAH box helicase domain and a pinin domain, respectively, showed perfect overlapping fluorescence signals with anti-ENO2 staining, suggesting that these two factors localized in the parasite nucleoplasm (Figure 2A). In contrast, two distinct patterns of fluorescence were observed for TgNF3 (gi number 211962881) and TgNF4 (gi number 211965453) with strong fluorescence in the parasite nucleolus in addition to a faint signal, which co-localized with ENO2 signal in the nucleoplasm (Figure 2A). In a similar approach, we confirmed the location of TgNF1 and TgNF2 in the parasite nucleoplasm (Figure 2B). Both diffuse nucleoplasm and strong nucleolar patterns were confirmed for TgNF3 and TgNF4 in transgenic HAFLAG-tagged protein, whose expression was driven by another promoter (Figure 2B). In contrast, using polyclonal antibodies specific to the two homologues of ancient archaeal chromatin-associated factors named Alba1 and Alba2 (Table S1), and here designated TgNF5 and TgNF6, fluorescence signals were mainly detected in the parasite cytoplasm (Figure 3A). However, we cannot rule out the presence of TgNF5 and TgNF6 in the parasite nucleus, as superimposition of ENO2 signal (red) and DAPI (blue) with TgNF5 or TgNF6 fluorescence (green) showed profiles, which significantly overlap on the nucleus periphery (Figure 3B), suggesting that these Alba homologues may have regulatory functions in both nucleus and cytoplasm, as previously described [34], [35], [37]. Antibodies specific to the candidate factor TgNF7 that has no known functions (Table S1) also showed dual cytoplasm and nuclear localization, which is similar to TgNF5 and TgNF6 (Figure 3A and 3B, lower panels). In addition, TgNF5, TgNF6 and TgNF7 were also detected in both nuclear and cytoplasm-enriched materials after sub-cellular fractionation followed by Western blots. Thus, we conclude that all seven factors experimentally tested herein are capable of entering the nucleus of *T. gondii*.

### Evidence for specific *T. gondii* promoter binding by TgNF1 and TgNF7

**Figure 4:**
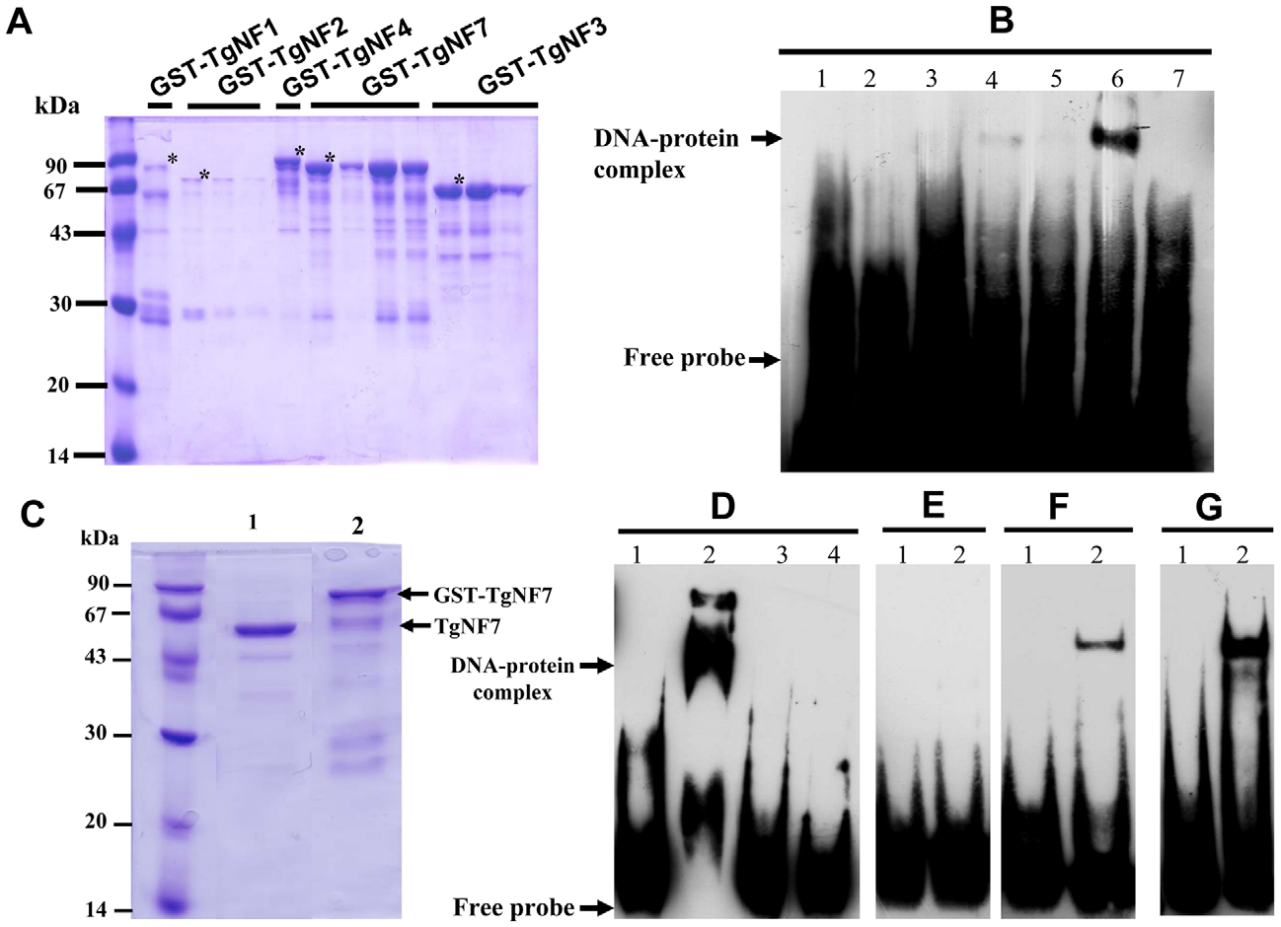
Evidence for specific TgNF7 protein-DNA interactions within the promoter of ENO1. A) Expression and purification of recombinant TgNF1, TgNF2, TgNF3, TgNF4 and TgNF7 proteins fused to GST. The recombinant factors tested were marked with asterisks. B) Electrophoretic band shift assays using recombinant GST alone (lane 1), free probe (lane 2), rTgNF4 (lane 3), rTgNF1 (lane 4), rTgNF2 (lane 5), rTgNF7 (lane 6), rTgNF3 (lane 7) and the biotinylated probe from ENO1 promoter described in Figure 1. C) Purification of rTgNF7 lacking the GST (lane 1), which has been removed by digestion with the PreScission protease. D) Gel shift binding assay using the 47-bp probe at the ENO1 promoter shown in Figure S1 (red) and pure rTgNF3. Lane 1 corresponds to probe alone; lane 2, DNA-rTgNF7 complex; lane 3, specific unlabelled competitor at 10-fold excess; lane 4, specific unlabelled competitor at 50-fold excess. E) Lane 2, unrelated 27-bp probe containing one G nucleotide with pure rTgNF7; lane 1, probe alone. F) Lane 2, unrelated probe containing 27-bp probe containing one GGGGG motif with pure rTgNF7; lane 1, probe alone. G) unrelated probe containing 27-bp probe containing two GGGGG motifs with pure rTgNF7; lane 1, probe alone.

To investigate whether any of these candidate factors can directly bind to *T. gondii* promoter, *E. coli* produced recombinant proteins fused to GST (Figure 4A, stars) were tested in gel shift assays (Figure 4B). Out of the five recombinant proteins, rTgNF7 strongly binds to the biotinylated ENO1 promoter (Figure 4B, lane 6), whereas TgNF1 appears to only weakly interact (Figure 4B, lane 4), as expected for a protein containing a putative DEAD/DEAH box helicase domain. Neither GST alone (Figure 4B, lane 1), nor the other three recombinant factors bound the probe (Figure 4B, lanes 3, 5 and 7). We have extensively scanned the whole ENO1 promoter sequence (Figure S1) for retarded DNA-protein complexes using purified rTgNF7 lacking GST (Figure 4C, lane 1). We identified a 47-bp DNA fragment (Figure S1, red) that specifically binds to rTgNF7 (Figure 4D, lane 2). The specific motif that binds rTgNF7 was then determined by successive mutations or replacements that lead to the identification of GGGGG (Figure S1, blue) as the genuine target motif of rTgNF7. The presence of the GGGGG motif is sufficient for rTgNF7 binding, and was proportional to the number of GGGGG motifs present (Figure 4E–4G). The direct binding of TgNF7 to the GGGGG motif present in *T. gondii* promoters suggested that only one of the three candidate factors with dual cytoplasm and nuclear localization has the DNA binding characteristics of a genuine nuclear factor, whose precise regulatory functions in the parasite await further investigation. With the exception of TgNF1 that contains a DEAD/DEAH box helicase domain, the other candidate factors are probably involved in protein-protein interactions required for promoter binding. We decided to investigate in more detail TgNF3, which shares similarities with nuclear FK506 binding proteins (FKBP), known in yeast as a histone chaperone that regulates rDNA silencing [33].

### *Tg*NF3 belongs to a large superfamily of nuclear chaperones, including insect and fungi FKBP, plant HD2 histone deacetylases and members of the nucleoplasmin family

**Figure 5:**
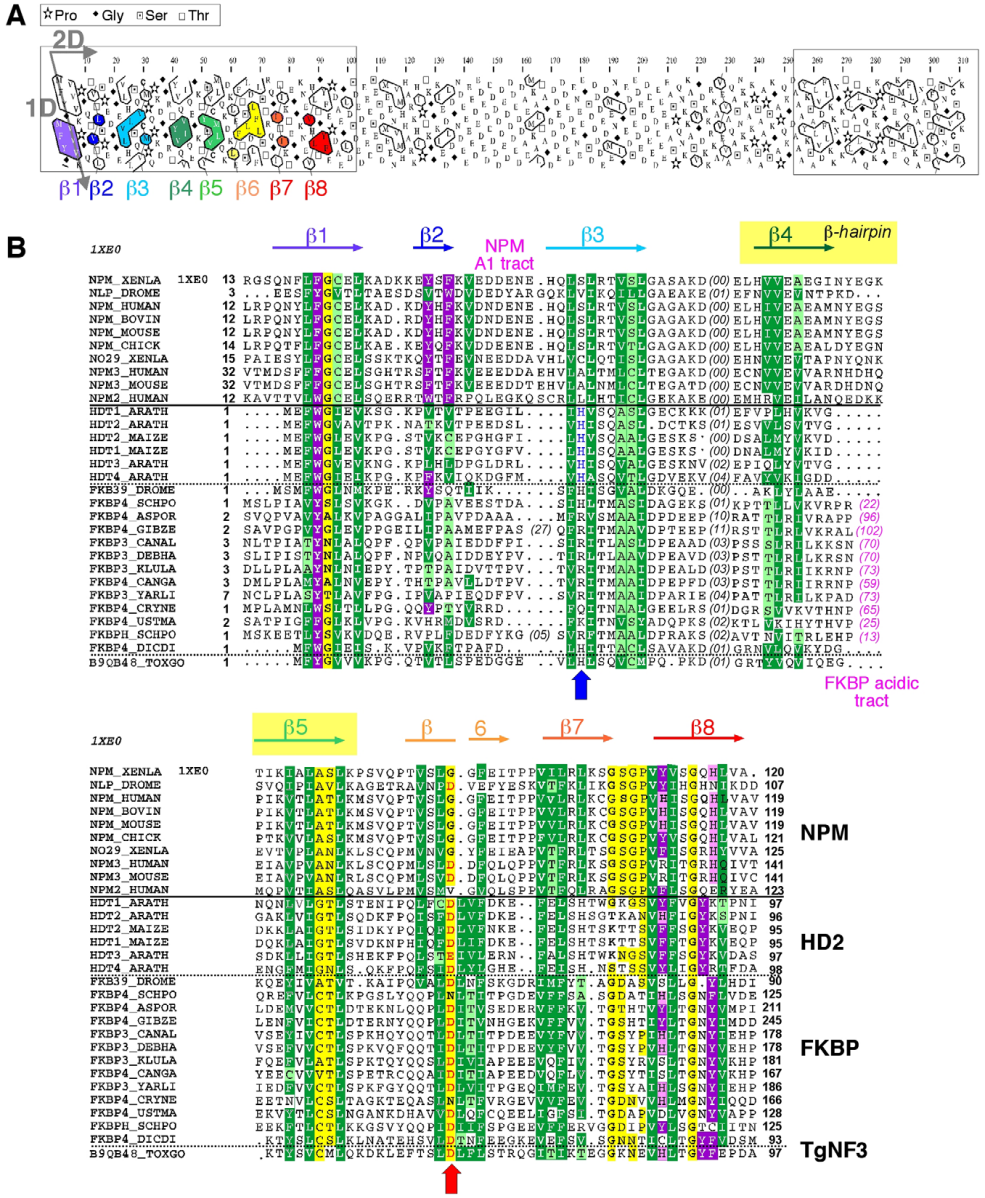
Analysis of the TgNF3 sequence, a member of the HD2/FKBP/NPM superfamily. A) Hydrophobic cluster analysis (HCA) plots of the TgNF3 sequence. The sequence is shown on a duplicated alpha-helical net, in which strong hydrophobic amino acids (VILFMYW) are contoured. These form hydrophobic clusters, which mainly correspond to regular secondary structures [38]. The way to read the sequence (1D) and secondary structures (2D) are indicated with arrows. The plot allows the delineation of globular domains (boxed), made of ∼33% of strong hydrophobic amino acids, which are distributed into clusters whose lengths are typical of those of regular secondary structures. Secondary structures indicated below the plot for the first globular domain were deduced from the similarities shared with the NPM family (see panel B). B) Multiple alignment of the TgNF3/HD2/FKBP/NPM family. The protein alignments were constructed on the basis of data from sequence similarity and fold recognition searches, and refined using HCA. UniProt identifiers are reported in front of the sequences. The secondary structures observed in the NPM experimental 3D structure (NPM_XENLA, pdb identifier 1XE0) are shown above the alignment, with the outstanding features of the NPM sequences (A1 tract, beta-hairpin) indicated. Conserved hydrophobic amino acids (VILFMYW) are indicated in green (light green for amino acids considered as valid substitutions (ACTS)), aromatic amino acids (FYW) in purple (pink for H, considered as a valid substitution), loop-forming (PGDNS) or small residues (AVC) in yellow. The two conserved amino acids described earlier for the HD2/FKBP family [39] are indicated with blue (H) and red (acidic amino acid) arrows.

**Figure 6:**
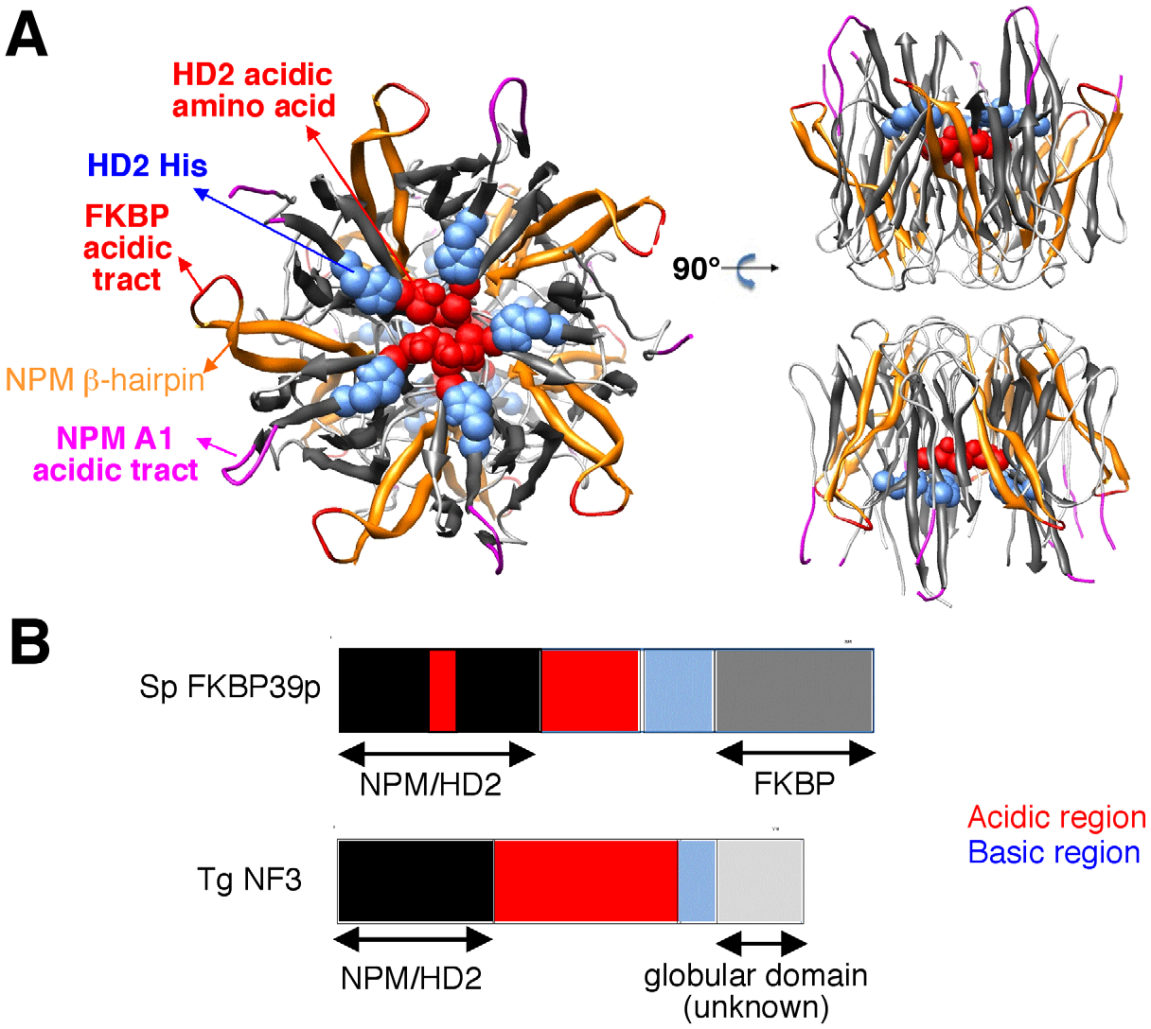
3D-structure of the NPM Np core domain, highlighting the positions of hallmarks of the NPM and HD2/nuclear FKBP/TgNF3 families. A) The NPM Np core domain, as observed in the *Xenopus laevis* No38 (pdb identifier 1xe0) consists in an eight-stranded beta-barrel, which is organized as a dimer of pentamers. On this template, two orthogonal views indicated the positions of the A1 acidic tract (pink), the beta-hairpin (orange), the FKBP acidic tract (red) that is typical of nuclear FKBPs, the HD2 conserved histidines (blue) and the acidic amino acids (red). B) Domain organization of SpFKBP39p and TgNF3. The NPM/HD2 domains in the two proteins are highlighted. An insertion of an acidic region is present in the middle of the yeast FKBP39 NPM/HD2 domain. Numbers indicate amino acid positions. The acidic and basic regions of both proteins are described.

Because TgNF3 was selected for further detailed molecular and functional characterization, we wanted to determine whether this putative homologue of fungi, nuclear FK506-binding protein is a genuine member of this family, and consequently is essential for nucleolar/nuclear functions in the parasite. A first analysis of the TgNF3 protein sequence using Hydrophobic Cluster Analysis (HCA) [38] indicated that it contains two globular domains (boxes, Figure 5A), separated by a linker sequence, rich in acidic residues (unboxed area, Figure 5A). A PSI-BLAST search using the first domain (aa 1–100) of TgNF3 as query indicated significant similarity with the histone deacetylase 2 (HD2)/nuclear FK506-binding protein (FKBP) family, which was first reported by Aravind & Koonin [39] (Figure 5B). The N-terminal domain of nuclear members of the FKBP family found in yeasts and insects has nucleosome assembly activity, which is independent of the activity of their C-terminal FKBP domain, having peptidyl-prolyl isomerase (PPIase) activity [33]. This N-terminal domain shares significant similarities with the N-terminal domain of HD2 proteins, which are described as plant-specific histone deacetylases (HDACs) [39]–[41]. Following the HD2 N-terminal domain alignment, it has been described that the two conserved polar residues, namely an invariant aspartic acid and a histidine (arrows on Figure 5B), may play a key role in lysine deacetylation [40], a prediction that was partly supported by a further experimental investigation [41]. The HD2/nuclear FKBP family, encompassing the N-terminal domains of plant HD2 and nuclear FKBPs found in fungi and insects, also includes the N-terminal domains of parasitic apicomplexan proteins. This family includes TgNF3, which also possesses the two conserved polar residues H25 and D67 (blue and red arrows on Figure 5B) but intriguingly, does not include vertebrate members [40]. Remarkably, after further PSI-BLAST iterations, we found significant similarities with the N-terminal (Np) core domain of the nucleoplasmin/nucleophosmin (NPM) family, which are nuclear chaperones from vertebrates involved in chromatin remodelling [42] (Figure 5B). Reciprocal searches using sequences from the NPM family also highlighted the similarity with the whole HD2/nuclear FKBP family. Thus, the N-terminal domains of the HD2/nuclear FKBP and nucleoplasmin/nucleophosmin (NPM) families form a unique large structural superfamily, sharing a common ability to bind histones. For all members of the family, the N-terminal Np core is followed by acidic stretches, thought to play an important role in histone binding (Figure 5B), but only in few cases, a C-terminal globular domain follows this acidic stretches as for nuclear FKBP, in which this domain has peptidyl-prolyl isomerase (PPIase) activity. For TgNF3, a small domain consisting of α-helices is also present, but it shares no obvious similarity with any other known domain. We thus present in this study a refined alignment between these two families, which contain well-conserved amino acids in some positions, in particular those occupied by hydrophobic amino acids (Figure 5B). Worth noting is that two basic and acidic residues thought to participate in the deacetylase activity in the HD2/nuclear FKBP family (arrows on Figure 5B) are not conserved in the NPM family, suggesting that the deacetylase function, if it is proven to be conserved in the HD2/nuclear FKBP family, might be lost in the NPM family. In addition, we provide a structural interpretation of the HD2/nuclear FKBP alignment using the data from crystal structures of NPM family members. These have revealed that the Np core adopts an eight-stranded beta-barrel structure and organizes itself into pentameric or decameric structures [43]–[45]. These decameric structures (dimers of pentamers) appeared to have direct relevance to histone binding. It has been proposed that histone octamers dock around the NPM decamer periphery, the binding especially involving an acidic stretch (NPM A1 tract) and a signature β-hairpin of the Np core (pink and orange in Figure 5B and 6A). However, the examination of our alignment showed that the NPM-specific acidic A1 tract is not present in the HD2/nuclear FKBPs. Instead, acidic stretches of variable length within the β-hairpin linking strands β4 and β5 are present in the nuclear FKBPs (FKBP acidic tract, Figure 5B). This insertion would be located in close proximity to the loop integrating the NPM A1 tract (Figure 5A). Moreover, we showed that the two residues that may play a key role in lysine deacetylation, namely an invariant aspartic acid and a histidine (arrows on Figure 5B) [39], [43]–[45], are located in close proximity on the NPM Np core (blue and red in Figure 6A), at the end of the funnel shaped cavity formed by the different subunits, and on the subunit distal face (thus opposite to the pentamer-pentamer interface). The two residues are also close to the positions of the acidic stretches and of the β-hairpin. We thus hypothesize here from this 3D mapping that the binding of histones on the NPM/HD2 scaffold may not involve the decamer periphery as previously suggested. Rather the distal ends with the pentameric organization may be probably important for histone binding. Even though the HD2/nuclear FKBPs would adopt a NPM-like quaternary association will require further experimental proofs, the recent electron microscopy observations showed that histones do truly interact with the NPM chaperone distal face [46]. These data are in good agreement with our hypothesis, and the schematic representation of Figure 6B also highlights that SpFKBP39 and TgNF3 have a common N-terminal domain with NPM/HD2, but differ in their C-terminal extremities. The C-terminal globular domain of *T. gondii* TgNF3, which is specific of apicomplexan parasites, has no similarity with the C-terminal propyl peptidyl isomerase of nuclear FKBPs and to other known proteins. Therefore, we embarked in the functional characterization of TgNF3 protein.

### TgNF3 gene is transcribed and translated in both virulent tachyzoites and avirulent bradyzoites of *T. gondii*

**Figure 7:**
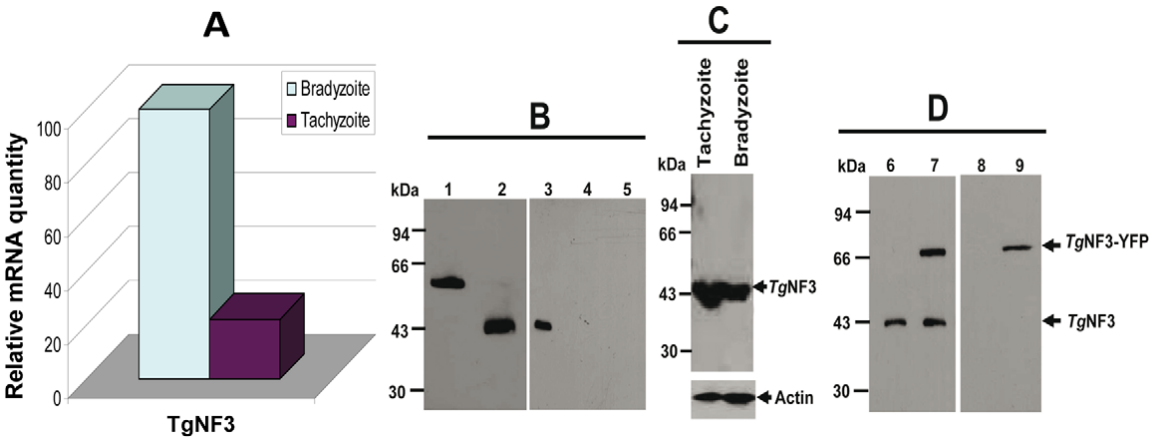
Comparison of the expression of *T. gondii* TgNF3 transcript and protein between tachyzoites and bradyzoites. A) Quantitative real-time RT-PCR analysis on total RNA prepared from tachyzoites cultivated *in vitro* and encysted bradyzoites isolated from brains of chronically infected mice. After first strand synthesis, the cDNA of TgNF3 as well as beta-tubulin cDNA (used as a control) was amplified by two specific primers as described in Materials and Methods. B) Western blot analysis of the bacterially expressed and purified recombinant TgNF3-GST fusion protein (lane 1), proteolytically cleaved and purified recombinant TgNF3 non-fusion protein (lane 2), total SDS-protein extracts from *T. gondii* tachyzoites of 76K strain (lane 3), from non-infected human fibroblast cells (lane 4) and from non-infected mouse brain cells (lane 5). The immunoblot was incubated with the affinity purified anti-TgNF3 antibodies. C) Western blot analysis of total SDS-protein extract prepared from purified tachyzoites and bradyzoites. In the upper panel, the immunoblot was incubated with the polyclonal antibodies anti-TgNF3 whereas the lower panel corresponds to duplicated blot loaded with identical amount of total SDS-protein extract analysed above and incubated with a monoclonal antibody anti-actin used to standardise equal amounts of parasite proteins. D) Ectopic expression of YFP tagged to TgNF3 protein (TgNF3-YFP) in stably transformed tachyzoites. The chloramphenicol-resistant transfectants were cloned and total protein extract of one positive clone shown on these blots (lane 7) was compared to that of wild type tachyzoites (lane 6). Note that the 70 kDa-protein, which corresponds to the TgNF3-YFP fusion protein, is exclusively detected in the transformed positive tachyzoites only. This was confirmed by the monoclonal antibody anti-GFP tested on the total SDS-extract protein of TgNF3-YFP expressers (lane 9) *versus* wild type tachyzoites (lane 8).

Towards the identification of the potential nuclear functions of TgNF3 during intracellular development of the parasite, we analyzed the pattern TgNF3 gene expression in the two invasive life stage forms of *T. gondii* present in intermediate hosts. Specific transcript coding TgNF3 were amplified by quantitative real-time RT-PCR using total RNA isolated from the rapidly replicating tachyzoites and the dormant encysted bradyzoites (Figure 7A). A comparison of the amount of specific transcripts between virulent tachyzoites and dormant bradyzoites was assessed by real-time qRT-PCR using a normalization step with transcript coding the housekeeping β-tubulin. Figure 7A showed that the level of TgNF3 mRNA is at least 5-fold more abundant in the persistent and dormant bradyzoites than in the virulent rapidly replicating tachyzoites. To investigate the functions of TgNF3 in *T. gondii*, we initially attempted to knockout the gene, but failed even in the Δku80 parasite strain that lacks random DNA integration [47], or when using the inducible-anhydrotretracyline system [48], suggesting that the *TgNF3* gene codes for an essential function and the locus may also be inaccessible to double homologous recombination. Therefore, we decided to ectopically express the *TgNF3* gene in transgenic parasites. Moreover, the quantitative real-time RT-PCR data (Figure 7A), which indicates that *TgNF3* transcript level is 5-fold lower in the rapidly replicating tachyzoites relative to the persistent dormant bradyzoites is not only pertinent to this alternative strategy, but also to investigate its biological relevance. We generated transgenic tachyzoites, which ectopically express TgNF3 fused to YFP. Polyclonal antibodies were also raised against purified recombinant TgNF3-GST fusion protein and Figure 7B shows the specificity of the anti-TgNF3 purified sera tested by Western blots using the recombinant nonfusionTgNF3 (lane 2), GST-TgNF3 fusion expressed in *E. coli* (lane 1), total extract proteins from *T. gondii* tachyzoites (lane 3), from uninfected human fibroblast (lane 4) and from brain cells (lane 5). The sera specifically recognized a single band corresponding to a 43-kDa protein in tachyzoites (lane 3), which is in good agreement with the expected molecular mass of TgNF3. This observation is also supported by the co-migration between the bacterial recombinant non-fusion TgNF3 protein (lane 2) and the native parasite TgNF3 protein (lane 3). Neither anti-GST antibodies nor the pre-immune mice sera reacted with *T. gondii* proteins on Western blots. To determine the pattern of expression of TgNF3 in the different life stages of the parasite, cell lysates of tachyzoites and bradyzoites were resolved by SDS-PAGE and probed by Western blots using purified antisera. Figure 7C illustrates the detection of similar levels of TgNF3 protein in tachyzoites and bradyzoites (upper panel), as immunoblots in parallel with monoclonal antibody anti-*Toxoplasma* actin provided loading controls (lower panel). These data suggest that the difference in TgNF3 transcript level does not correlate with the amount of protein detected in the two invasive life stage forms of *T. gondii*. We also transfected parasites with a vector expressing TgNF3 tagged to YFP under the control of the constitutive TUB1 promoter (p*TUB*1-TgNF3-YFP). These and other expression vectors used in this study are depicted schematically in Figure 2 (on the top) and the stable parasites were selected by chloramphenicol and cloned. Western blot analysis of total protein extracts from transgenic parasites expressing TgNF3 (Figure 7D, arrowheads) confirmed that both native TgNF3 (43-kDa protein) and transgenic TgNF3-YFP fusion protein (∼70-kDa protein) were detected with anti-TgNF3 antibodies (lane 7). As anticipated, only TgNF3 was recognized in the wild type parasites (lane 6). The monoclonal antibody specific to GFP recognized only the TgNF3-YFP fusion protein in the transgenic tachyzoites (lane 9) whereas no protein was detected in wild type tachyzoites (lane 8), as expected. It should be noticed that we choose to study a transgenic line where ectopic levels of TgNF3-YFP were roughly equivalent to endogenous ones (Figure 7D, lane 7), as we did not want over-expression of TgNF3-YFP to lead to down-regulation of endogenous TgNF3 levels. Nonetheless, in these transgenic parasites the sum of TgNF3 levels is made up of both ectopically expressed TgNF3-YFP plus endogenous TgNF3. As a consequence, overall TgNF3 protein levels are approximately 2-fold higher than in wild type parasites.

### Dynamics of TgNF3, a prominent nucleolar factor of the virulent tachyzoites of *T. gondii*

**Figure 8:**
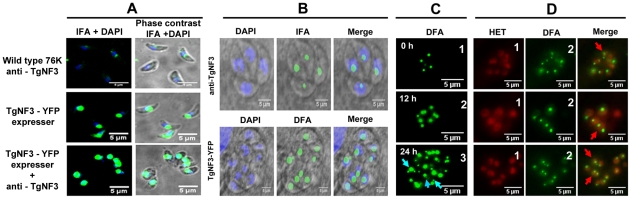
Native TgNF3 and transgenic TgNF3-YFP proteins are preponderant nucleolar resident factors. A) Confocal images of extracellular tachyzoites expressing TgNF3 protein. The upper panel shows a low level of endogenous TgNF3 in the extracellular tachyzoites of the wild type 76K strain detected with the polyclonal anti-TgNF3 antibodies. The middle panel corresponds to extracellular transgenic tachyzoites over-expressing ectopic TgNF3-YFP whose fluorescent signal was directly captured. The lower panel corresponds to extracellular transgenic tachyzoites ectopically expressing TgNF3-YFP protein incubated with the anti-TgNF3 antibodies. B) Confocal image of intracellular dividing tachyzoites stained with the purified polyclonal antibodies anti-TgNF3 and DAPI (upper panel). The lower panel corresponds to direct fluorescence captured by confocal microscopy on the fixed intracellular tachyzoites ectopically expressing TgNF3-YFP, which were also stained with DAPI. Note that the fluorescence signal is mostly present in the nucleolus, which is not stained by DAPI. C) Time lapse imaging of fluorescence in living dividing tachyzoites, which stably express ectopically TgNF3-YFP. One vacuole containing four daughter parasites were selected at time 0 (0 h) and constantly followed during intracellular development and parasite replication for 24 h. In these experiments, no nuclear staining dye was used. The blue arrows indicate the presence of one or two smaller nucleoli close to a large nucleolus in the parasite nucleus. D) Three separate sets of intracellular replicating tachyzoites, which stably express ectopically TgNF3-YFP in the presence of hydroethidine (HET), a living nuclear dye staining (red fluorescence), prior to direct imaging of fluorescence (green). In the merged pictures, the red arrows indicate nucleolar fragmentation or its *de novo* synthesis during direct fluorescence experiment in which hydroethidine (HET) dye has been used to confirm the presence of smaller nucleoli with a large nucleolus in a single given parasite.

We next compared the localization of TgNF3 in parental and TgNF3-YFP ectopically expressing tachyzoites using purified anti-TgNF3 antibodies, YFP-direct fluorescence detection or both anti-TgNF3 and YFP signals. Figure 8A shows confocal images of extracellular transgenic tachyzoites that ectopically express TgNF3-YFP. In contrast to nucleoli with weaker fluorescence signal in the parental tachyzoites, stained with the anti-TgNF3 antibodies (Figure 8A, upper panels), the ectopically TgNF3-YFP expressers contain nucleoli with strong fluorescent signals, which occupied an important proportion of the nuclear volume of the parasites (Figure 8A, middle panels). This conclusion is further supported by an even stronger signal of enlarged nucleoli observed in transgenic TgNF3-YPF expressers, stained with the anti-TgNF3 antibodies (Figure 8A, lower panels), thereby confirming the relative over-expression of TgNF3 protein in the ectopically expressing TgNF3-YFP parasites as described by Western blot experiments (Figure 7). In addition, Figure 8B shows confocal images of intracellular transgenic tachyzoites ectopically expressing TgNF3-YFP (lower panel), which contain nucleoli with stronger fluorescent signals, which often occupied a large proportion of the nucleus volume as compared to parental tachyzoites (upper panels). These data confirm that TgNF3 is most prominent in the nuclear areas, which defines *T. gondii* nucleoli and the protein contains itself all the sequence information required for nucleolar retention after being targeted into the nucleus.

Having observed the presence of TgNF3 in the parasite nucleoli, we determined the dynamics of its expression by live imaging using time-lapse video-microscopy. The direct fluorescence collected for TgNF3-YFP in the intracellular tachyzoites revealed in some cases the presence of two smaller nucleoli in close vicinity of one larger nucleolus (Figure 8C, blue arrows), suggesting a dynamic biogenesis of the nucleolus in *T. gondii*. To determine the precise location of smaller sized nucleoli in the nucleus, we simultaneously introduced hydroethidine (HET), a live nuclear dye staining during time-lapse imaging. Tracking three sets of independent vacuoles containing actively replicating TgNF3-YFP expressing tachyzoites confirmed two smaller sized and spherical nucleoli that positioned separately in the nucleus stained by HET (Figure 8D, red arrows) although a less intense staining was observed in the entire nuclear areas, which defines the nucleoplasm surrounding the nucleolus. In some cases, only one very small and spherical nucleolus was also detected. These two complementary approaches revealed the dynamics of nucleolar biogenesis during the intracellular replication *of T. gondii* tachyzoites *in vitro*, a phenomenon that so far has not been reported. Collectively, these data demonstrate that TgNF3 is predominantly a resident factor of the parasite's nucleolus even if it is also convincingly detected in the other areas of the nucleus, likely in the nucleoplasm.

### Ectopic expression of TgNF3 induces an increased nucleolus size

**Figure 9:**
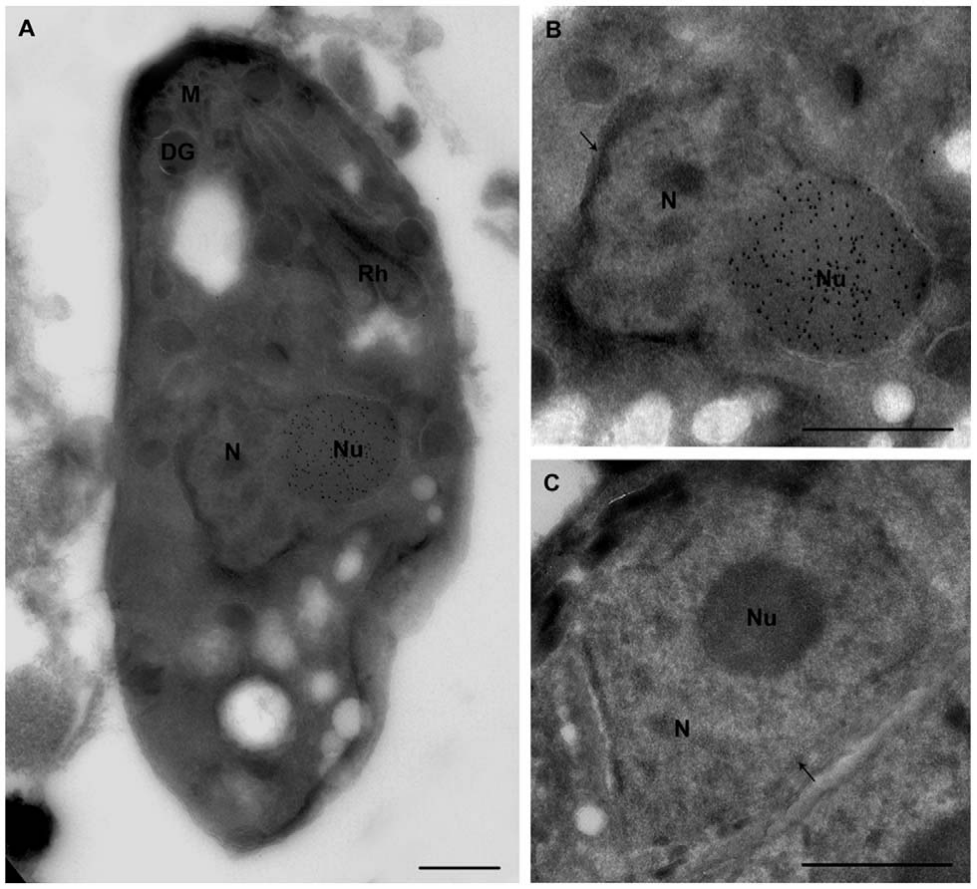
Ultrastructural analyses show that ectopic expression of TgNF3-YFP enhanced considerably nucleolar size. A) This electron micrograph corresponds to cryo-immunogold detection of one longitudinal section of transgenic tachyzoite TgNF3-YFP expressers. B) An enlarged picture of the parasite area containing the immunogold stained nucleus of the tachyzoite ectopically expressing TgNF3-YFP shown in panel A. C) Enlarged section of a negative control as the nucleus of parental wild type 76K tachyzoite incubated with anti-TgNF3 antibodies shows no gold labelling. N, nucleus; Nu, nucleolus; arrow indicates the nuclear membrane; Rh, rhoptries; M, micronemes; DG, denses granules. The scale bar = 500 microns.

The nucleolar localization of TgNF3 was also demonstrated using electron microscopy and cryo-immunogold labeling performed using intracellular tachyzoites ectopically expressing TgNF3-YFP and a monoclonal antibody specific to GFP (Figure 9). Using immuno-gold staining and electron microscopy, we also discovered that the expression of the YFP-tagged version of TgNF3 induced profound changes in morphology of the parasite nucleus with a considerable increase of nucleolus size (Figure 9A and 9B). This increased nucleolus size also represents an unusual feature caused by TgNF3 over-expression in the parasites. These observations were confirmed in all transgenic extracellular or intracellular tachyzoites, which ectopically expressed TgNF3-YFP. Using confocal acquisitions that allow 3D-reconstruction of the whole tachyzoite's body, we showed that tachyzoites ectopically expressing TgNF3-YFP contain nucleoli with a remarkable increase in size (Video S2) relative to that of the parental parasite (Video S1). We conclude that the most direct effect of ectopic expression of TgNF3-YFP in all cases is the presence of huge nucleoli very close to nuclear membrane with a protuberance at one pole of the nucleus, thus deforming the nuclear shape externally (Figure 9B). As a negative control, the nucleolus in the wild type parasite incubated with anti-GFP show no gold labelling and the nucleolus consistently appeared normal in size and was often centrally positioned in the nucleus (Figure 9C). We estimate that ectopic expression of TgNF3 protein induces about 4–5 fold-increase in nucleolar size of transgenic parasites (Figure 9B), relative to that of the wild type tachyzoites (Figure 9C). In addition, when the anti-TgNF3 antibodies were incubated with the wild type tachyzoites, following by gold labelling, the nucleolus remained normal in size and position. In this case, the nucleolus of the ectopic expressing TgNF3-YFP again appeared larger and positioned towards the periphery of the nucleus.

### Ectopic expression of TgNF3 enhances parasite replication *in vitro* but drastically attenuates parasite virulence *in vivo*

**Figure 10:**
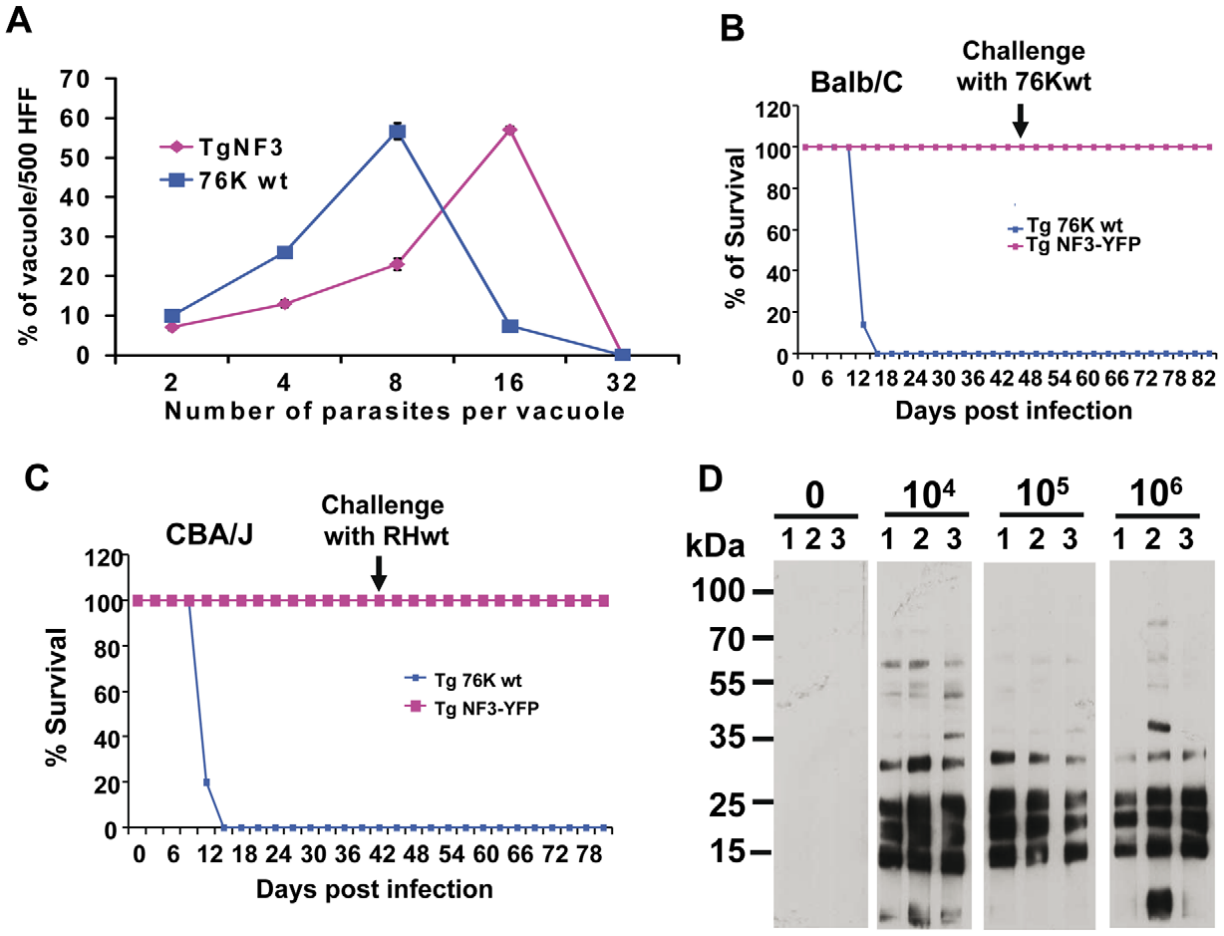
Ectopic expression of TgNF3 enhances parasite replication *in vitro* but drastically attenuates parasite virulence *in vivo*. A) The percentage distribution of vacuole size (number of parasites/vacuole) was determined at 24 h after infection with the tachyzoites of the parental 76K strain and transgenic ectopically expressing TgNF3-YFP. The parasites were grown in fibroblast cells under classical culture conditions, stained and counted. The number of parasites per vacuole was counted for 500 vacuoles in three different experiments (P<0.001). The relative time-to-death of mice infected with TgNF3-YFP expressers and parental wild type 76K strain were compared. B) A group of 10 females of BALB/c mice was inoculated with lethal doses (10^4^ parasites) of tachyzoites, and mortality was monitored over 6 weeks. C) A group of 10 females of CBA/J was also inoculated with lethal doses (10^4^ parasites) of tachyzoites, and mortality was monitored over 6 weeks. Mice infected with TgNF3-YFP expressers survived whereas all of those infected with wild type tachyzoites died. The surviving mice were then challenged at 42 days post-infection either with 10^5^ tachyzoites from 76K strain or 10^5^ tachyzoites of the highly virulent RH strain, and monitored for life expectancy and protection. Three independent experiments have been performed (n = 3) and P<0.001. D) A group of mice were infected with 10^4^, 10^5^ and 10^6^ TgNF3-YFP expressing tachyzoites, respectively. Infected mice survived as above and at 42 days post-infection, the serum of each surviving mouse was tested on total SDS-protein extract of *T. gondii* by Western blots. These experiments were performed twice with identical results.

**Figure 11:**
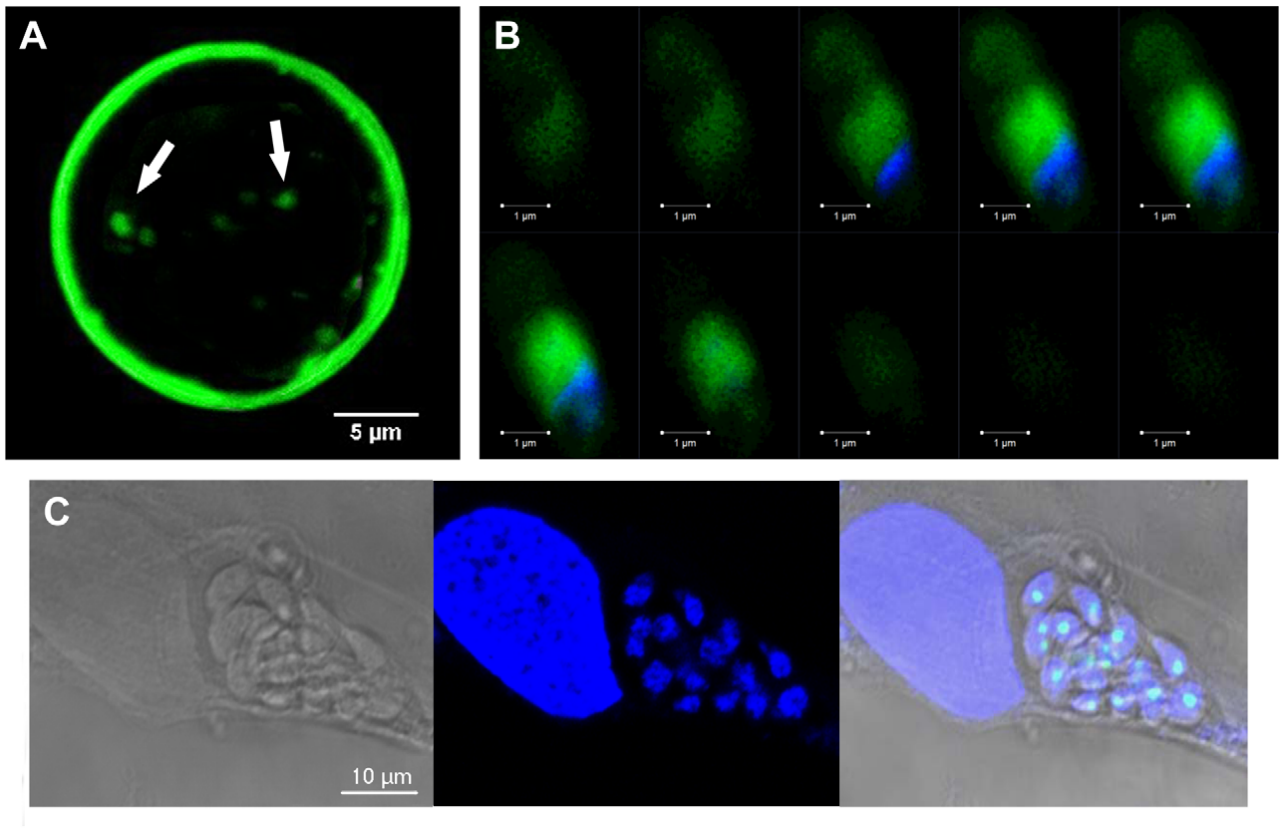
TgNF3 is present exclusively in the cytoplasm of dormant bradyzoites and relocates in the nucleolus during bradyzoite to tachyzoite conversion. A) Superimposition of five confocal images of one representative cyst, which was isolated from the brain of the infected mice analyzed above, stained specifically with the Dolichol biflorus FITC-labeled lectin, which recognizes the cyst wall. The fluorescence of bradyzoites expressing TgNF3-YFP was also simultaneously visualized (see also Video S3 and S4). Z-stacks were generated in increments of 0.45 µm. B) Z-stack confocal acquisitions of one representative bradyzoite expressing TgNF3-YFP released from cysts isolated from infected mice brain as described above, fixed and nuclear stained with DAPI. Z-stacks corresponding to 10 confocal images, which were generated in increments of 0.6 µm. Note that the fluorescence signal is restricted to the cytoplasm of the bradyzoites. C) Relocation of TgNF3-YFP in the nucleolus of newly converted tachyzoites, which are dividing inside human fibroblast cells inoculated by transgenic bradyzoites expressing TgNF3-YFP at 36 h post-infection.

Figure 10A shows that ectopic expression of TgNF3-YFP in tachyzoites of *T. gondii* induces a faster replication rate relative to the parental parasites. Ectopic expression of TgNF3 protein harbouring a C-terminal HA-FLAG tag leads to a similar increase in replication rate *in vitro* as TgNF3-YFP transgenic tachyzoites. The increased replication rate is not correlated to the elevation of host cell invasion since there is no significant difference in fibroblast cells or macrophage cells entry between these transgenic lines and wild type parasites. In addition, neither stable transgenic tachyzoites ectopically expressing another nuclear factor TgNF2 nor transgenic tachyzoites ectopically expressing the nuclear enolases (ENO1 and ENO2) displayed changes in nucleoli morphology and in replication rate relative to wild type parasites using the same type II 76K strain (our unpublished data). This suggests that the increased replication rate may be specific to TgNF3. Therefore, we conclude that TgNF3 may be involved in nucleolar dynamics and in functions that are important for the replication rate of *T. gondii* growing inside the host cells. To assess the influence of stable TgNF3-YFP expressers *in vivo*, the transgenic TgNF3-YFP and parental wild type parasites were used to inoculate a group of 10 mice at doses up to 10^4^ tachyzoites. After 4–5 days of infection, all mice from two genetically distinct groups (Balb/c and CBA/J), infected with the parental tachyzoites show the same characteristic symptoms of disease and succumbed approximately 12–14 days (Figure 10B and 10C). Surprisingly, all mice (Balb/c or CBA/J) infected with tachyzoites ectopically expressing TgNF3-YFP displayed only mild symptoms of disease and recovered faster than those infected with the parental tachyzoites. In this case, 100% of mice survival was obtained with the two groups infected with TgNF3-YFP expressers (Figure 10B and 10C). In order to ensure that the mice that survived had truly been infected, immune sera of each group of infected mice were collected 30 days post-inoculation and were tested in Western blots. All mice infected with the TgNF3-YFP expressers were positive and they also survived a subsequent challenge with 10^4^ RH (type I strain) and 10^5^ 76K (type II strain) wild type tachyzoites, doses that confer 100% mortality in the primo-infected mice used as controls (Figure 10B and 10C). These data were confirmed when a group of mice was respectively infected with 10^4^, 10^5^ and 10^6^ tachyzoites ectopically expressing TgNF3-YFP, again no mice succumbed and all reacted positively against *T. gondii* total protein extracts as above. All sera from surviving mice recognized parasite antigens ranging mostly from 15–35 kDa whereas the sera from non-infected mice failed to detect any *T. gondii* antigens, as expected (Figure 10D). These results demonstrated that the mice were truly infected with tachyzoites TgNF3-YFP expressers and consequently, positive immune responses have been developed. Therefore, we conclude that ectopic expression of TgNF3-YFP attenuates *T. gondii* virulence in mice and induces live vaccination in mice that can confer protection against *T. gondii* challenge. To examine if the attenuated TgNF3-YFP expressers were able to establish a chronic infection in mice, we searched for the presence of cysts using specific staining with the *Dolichos biflorus* lectins, which specifically labeled the cyst wall [49]. The presence of few cysts *per* brain with fluorescently positive cyst-wall stained with the lectin and the presence of YFP-expressing encysted dormant bradyzoites (Figure 11A) was indicative of a chronic infection in all survived mice monitored and analyzed. Movies representing cysts at different depth and created with the Zen software, at a rate of 5 frames per second, confirmed the presence of encysted bradyzoites that expressed YFP-TgNF3 proteins (Video S3). These observations were also validated by direct confocal imaging of lectin-unlabelled cysts by Z-stacks acquisitions enabling the 3D localization of fluorescent signals (Video S4). Interestingly, all cysts analyzed in TgNF3-YFP infected mice, which were challenged with either RH or 76K parental tachyzoites contained only YFP-positive encysted bradyzoites, suggesting that mice infection with transgenic tachyzoites expressing TgNF3-YFP raises an efficient sterile protection against *T. gondii*.

### TgNF3 is present exclusively in the cytoplasm of dormant bradyzoites and relocates in the nucleolus during bradyzoite to tachyzoite conversion *in vitro*

**Figure 12:**
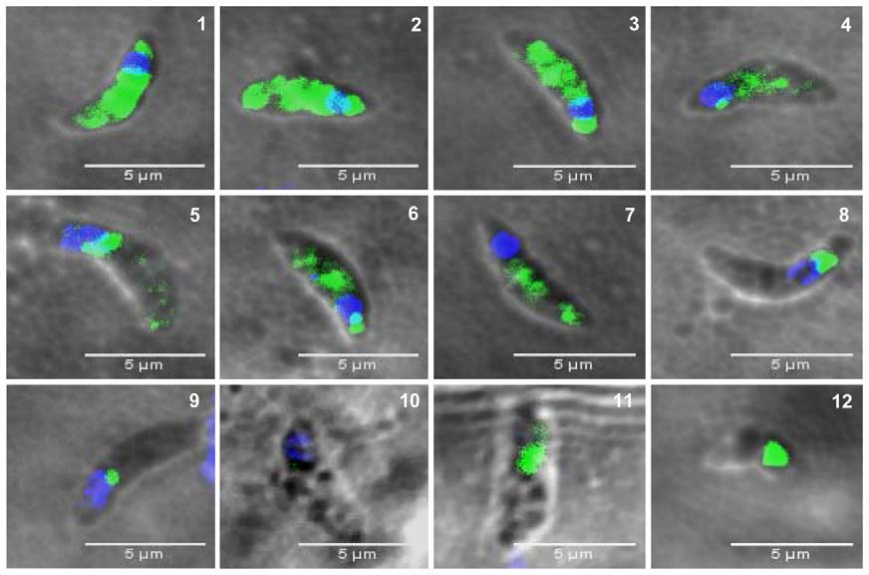
The presence of TgNF3 in the cytoplasm of dormant bradyzoites is accompanied by profound changes in parasite's nucleus. The bradyzoites released from wild type *T. gondii* 76K cysts isolated from chronically infected mice were formaldehyde-fixed and loaded on glass slides. The bradyzoites were incubated with the purified polyclonal antibodies specific to TgNF3 and processed for confocal imaging. Panels 1 to 12 correspond to 12 individual positive bradyzoites, which confirmed the presence of TgNF3 signal exclusively in the cytoplasm of these dormant bradyzoites. The Video S5 and S6 show the rotation at 360° around the y axis, at a rate of 5 frames per second, which demonstrate the complete absence of fluorescence and TgNF3 protein in the nucleus of the encysted dormant bradyzoites. TgNF3 is detected only in the parasite cytoplasm. In addition, note that the nuclei of slowly dividing bradyzoites displayed a remarkable half reduction in size (panels 4, 6 and 7), probably due to nuclear condensation relative to that of rapidly replicating tachyzoites. All bradyzoites analyzed do not contain nucleoli. Few bradyzoites also contain degenerated nucleus (panels 8, 9 and 10) and few lack the whole nucleus (panels 11, 12). The dramatic reduction in TgNF3 protein level in the cytoplasm of bradyzoites (panels 1 and 9 or 2 and 10) can be correlated with the degeneration and disappearance of nucleus in the bradyzoites.

The confocal observations described above confirmed the presence of TgNF3-YFP expression in the encysted bradyzoites during chronic infection of mice. We next determined whether TgNF3-YFP factor actually displayed the dual nucleolar/nuclear localization in the dormant encysted bradyzoites of *T. gondii*. Surprisingly, we found that bradyzoites isolated from brains of infected mice expressed TgNF3-YFP proteins exclusively in the cytoplasm, as demonstrated by ten Z-stack acquisitions, which allows the whole bradyzoite's body to be visualized at different depth (Figure 11B). Interestingly, when the bradyzoites were used to infect fibroblast cells and growth *in vitro* for 36 hours, TgNF3-YFP factor was again relocated in the nucleoli of all intracellular newly transformed tachyzoites (Figure 11C). The location of native TgNF3 in the cytoplasm of bradyzoites from the wild type of *T. gondii* 76K strain was also validated using anti-TgNF3 antibodies (Figure 12). We invariably detected native TgNF3 in the cytoplasm of all wild type bradyzoites and the signals varied from very intense fluorescent signal (panels 1–3) to weaker cytoplasmic signal (panel 4–7) and to very focused signal close to the nucleus [8]–[9]. Despite the presence of some apparent overlapping signal between the cytoplasm and nucleus (panel 1–3), 3D constructions of panels 1 and 2 from Figure 12 showed that TgNF3 signal is exclusively localized to the cytoplasm of the bradyzoites (Video S5 and S6). This novel localization of TgNF3 becomes obviously apparent when TgNF3 signal decreased in some dormant encysted bradyzoites (panels 6–7). During the course of the confocal imaging, we discovered that the nucleus, which is more posterior in bradyzoites, displays a half reduction in size relative to nucleus of tachyzoites. In some cases, profound alterations (panels 8–10) and a total absence of nucleus were observed in few bradyzoites (panels 11 and 12). The alterations of nuclear morphology and the complete lack of nucleus always correlated with the strong decrease in TgNF3 signals. In the meantime, we also observed the striking absence of nucleoli in all bradyzoites analyzed (Figure 12). 3D-reconstructions of confocal imaging of the entire bodies of all bradyzoites investigated confirmed the exclusive presence of TgNF3 in the cytoplasm of these dormant encysted *T. gondii* forms, suggesting that the nucleolar and nuclear functions of TgNF3 are only operating in the rapidly replicating and virulent tachyzoites. Therefore, we decided to further investigate in more detail how TgNF3 protein functions biochemically *in vitro* and *in vivo*.

### TgNF3 physically interacts with both free core and nucleosome-associated histones

**Figure 13:**
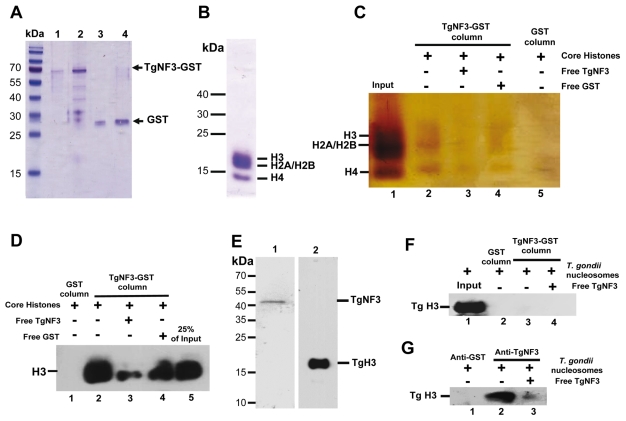
TgNF3 associates with core and nucleosome-associated histones *in vitro*. A) The purified recombinant protein TgNF3-GST or GST alone were coupled to glutathione-Sepharose 4B and the equivalent of 0.5 µg (lanes 1and 3) and 2 µg (lanes 2 and 4) of bound proteins were boiled and electrophoresed on SDS-PAGE, in order to verify that equal amounts of GST and TgNF3-GST bound protein were being used for the pull down assays. B) The quality of purified core histones containing histone H3, H4, H2A/H2B were checked by SDS-PAGE and coomassie blue staining. C) Silver staining of purified core histones of HeLa cells pulled down by TgNF3-GST. Lane 1, input corresponding to core histones; lane 2, TgNF3-GST incubated with core histones; lane 3, competition assay using non-fusion recombinant TgNF3 incubated with core histones prior to pull down with TgNF3-GST beads; lane 4, competition assay using GST alone incubated with core histones prior to pull down with TgNF3-GST beads; 5, GST bound beads incubated with core histones. D) Western blot after TgNF3-GST pull down using HeLa cell core histones and specific anti-histone H3 antibodies. Lane 1, GST alone incubated with core histones; lane 2, TgNF3-GST incubated with core histones; lane 3, competition assay using non-fusion recombinant TgNF3 incubated with core histones prior to pull down with TgNF3-GST beads; lane 4, competition assay using GST incubated with core histones prior to pull down with TgNF3-GST beads; lane 5, 25% of input corresponding to core histones. E) Western blots of total protein extract from purified *T. gondii* nucleosomes. Lane1, *T. gondii* nucleosomes probed with specific anti-TgNF3 antibodies; lane 2, *T. gondii* nucleosomes probed with specific anti-histone H3 antibodies. F) Western blot after TgNF3-GST pull down using *T. gondii* nucleosomes and specific anti-histone H3 antibodies. Lane 1, input corresponding to *T. gondii* nucleosomes; lane 2, the beads containing GST alone were incubated with *T. gondii* nucleosomes; lane 3, the TgNF3-GST beads were incubated with *T. gondii* nucleosomes; lane 4, competition assay using non-fusion recombinant TgNF3 incubated with TgNF3-GST beads prior to pull down with *T. gondii* nucleosomes. G) Western blot after immunoprecipitation using *T. gondii* nucleosomes and anti-TgNF3 antibodies. Lane 1, the anti-GST antibodies were incubated with *T. gondii* nucleosomes; lane 2, the anti-TgNF3 antibodies were incubated with *T. gondii* nucleosomes; lane 3, the anti-TgNF3 antibodies were incubated with non-fusion recombinant TgNF3 prior to addition of *T. gondii* nucleosomes. The immunoblot was revealed with the antibodies specific to histone H3.

Because we found a striking structural similarity between TgNF3, nuclear FKBP and nucleoplasmin-like proteins, which function as chaperones binding directly to histones and assemble histone octamers involved in nucleosome activity [42]–[46], [50], we asked whether TgNF3 can associate with purified mammalian core histones. To this goal, purified recombinant GST-tagged TgNF3 and GST alone produced in *E. coli* were purified and immobilized on glutathione-Sepharose, which was used to perform a series of pull-down experiments. To ensure that equal amount of TgNF3 and GST proteins were being used, aliquots of bound TgNF3-GST and GST alone to beads were eluted in SDS buffer and analyzed by SDS-PAGE (Figure 13A). Afterwards, the beads containing TgNF3-GST or GST alone were incubated with purified core histones from HeLa cells, which were tested for quality before use (Figure 13B). Only TgNF3-GST was found to specifically pull down the core histones of HeLa cells, as shown by SDS-PAGE and silver staining of Figure 13C (lane 2). No binding was observed when the GST alone was used (Figure 13C, lane 5). In addition, the level of core histones pulled down by TgNF3 was significantly reduced by direct competition assay in which the core histones were incubated with recombinant non-fusion TgNF3 protein prior to pull down experiments (Figure 13C, lane 3). No significant decrease of bound core histones was observed by competing with the GST alone (Figure 13C, lane 4). The specific binding of TgNF3 to histones was also confirmed when the same pulled down material analyzed by silver staining above was also subjected to Western blots using anti-H3 antibodies (Figure 13D). We found that approximately 25% of the original input bound specifically to TgNF3 (Figure 13D, lane 2). We confirmed the specificity of the competition with non-fusion recombinant TgNF3 (Figure 13D, lane 3). In addition, no binding of core histones to GST alone was observed (Figure 13D, lane 1). Next, we showed that *T. gondii* histone H3 (here named TgH3, lane 2) and TgNF3 (lane 1) are present in nuclesomes, which were purified from isolated nuclei of *T. gondii* (Figure 13E). In addition, we further demonstrated that histone TgH3 cannot be pulled down by GST-TgNF3 beads, suggesting that its association with native TgNF3 in the parasite nucleosome prevents binding site recognition and histone TgH3-TgNF3 complex formation (Figure 13F). This conclusion is supported by reciprocal immunoprecipitation using anti-TgNF3 antibodies, which validated TgNF3 as a genuine nucleosome-associated factor that interacts directly and specifically to *T. gondii* histone H3, present in parasite nucleosomes (Figure 13G). Altogether, these data support the notion that *T. gondii* TgNF3 has histone binding activity that is likely required for nucleosome functions in *T. gondii*.

### Genome-wide TgNF3 occupancy defined by ChIP-seq identifies genes involved in parasite metabolism, transcription and translation

**Figure 14:**
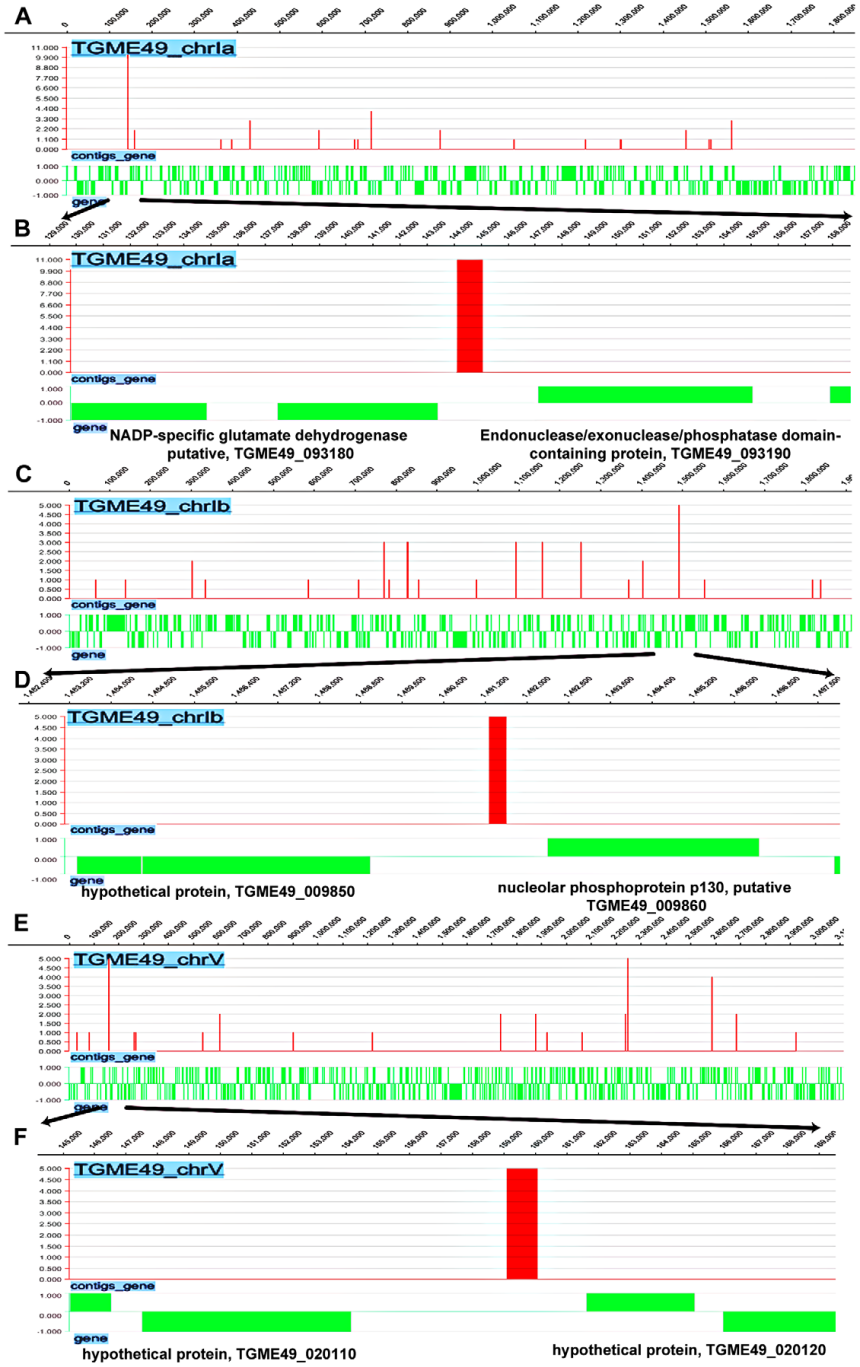
Genome-wide TgNF3 occupancy of gene promoters by ChIP-seq. A) Linear representation of Chr1a and TgNF3 binding sites. B) Enlargement of area showing Chr1a region comprised between 120000–158000 bp with a site of TgNF3 occupancy noted at 1 kb relative to gene coding NADP-specific glutamate dehydrogenase putative, (TGME49_093180) and 2 kb relative to gene coding endonuclease/exonuclease/phosphatase domain-containing protein (TGME49_093190). C) Linear representation of Chr1b and TgNF3 binding sites. D) Enlargement of track showing Chr1b region comprised between 1,482,400–1,497,800 bp with a site of TgNF3 occupancy noted at 0.8 kb relative to gene coding nucleolar phosphoprotein p130, putative (TGME49_009860) and 2.4 kb relative to gene coding hypothetical protein (TGME49_009850). E) Linear representation of ChrV and TgNF3 binding sites. F) Enlargement of track showing ChrV region comprised between 145000–169000 bp with a site of TgNF3 occupancy noted at 2 kb relative to gene coding hypothetical protein (TGME49_020120) and 5 kb relative to gene coding hypothetical protein (TGME49_020110).

We next investigated the direct and physical interactions of TgNF3 with promoter sequences *in vivo* using chromatin immunoprecipitation followed by high-throughput sequencing (ChiP-seq). Towards this goal, intracellular and actively dividing tachyzoites of wild type *T. gondii* 76K strain were fixed by formaldehyde, released from host cells before chromosome fragmentation by sonication and chromatin was immunoprecipitated using specific anti-TgNF3 antibodies, or pre-immune sera used as ChIP negative control. Both immunoprecipitates from specific anti-TgNF3 and pre-immune sera were subjected to high-throughput sequencing and bioinformatics analyses using genome data from http://www.toxodb.org. After comparison of sequences and removal of common genes targeted by both pre-immune and specific anti-TgNF3 sera, 5'untranslated regions corresponding to putative promoters of 516 genes were found to be exclusively pulled down by the anti-TgNF3 antibodies (Table S2). Figure S2 shows the schematic representations of TgNF3 hits on all 14 chromosomes of *T. gondii*. Among genes identified were 50% (264) of gene promoters expressing hypothetical proteins, 15% of metabolic enzymes, 5% of translation factors and 2.5% of transcription proteins. Interestingly, ChIP-seq also identified gene promoters corresponding to putative NADP-specific glutamate dehydrogenase with the highest hits (11 hits), DEAD/DEAH box helicase containing protein (5 hits), nucleolar phosphoprotein and histone deacetylases (Figure 14 and Table S2). It is worth noting that theses enzymes or factors have also been identified as candidate nuclear factors during the affinity purification of nuclear factors that bind to ENO1 promoter and the proteomics analyses reported in this study (Table S1). In addition, several gene promoters regulating expression of proteins involved in RNA metabolism and protein synthesis such as essential amino-acyl tRNA (histidine, lysine, tyrosine, methionine) were also found. The presence of several gene promoters of nucleolar factors, ribosomal proteins and RNAs is consistent with TgNF3 localization and its functions in the nucleolus. In addition, the presence of DNA-directed RNA polymerase II and RNA polymerase II subunits also supported the notion that TgNF3 is likely involved in the regulation of genes, which are present in other areas of the nucleus. Finally, several promoters of genes coding for kinases, Ras family and related regulatory factors, and factors involved in cell division were also found, suggesting that binding and regulation of genes of these later factors may be part of mechanisms involved in the rapid replication rate of TgNF3-YFP ectopic expressers. However, the regulation of the numerous parasite metabolic processes, protein synthesis through the control of translation might underlie the growth rate of the transgenic ectopically expressing TgNF3-YFP. It is worth noting that excepting a few putative and yet uncharacterized promoters of genes coding for putative kinases of rhoptries, the vast majority of promoters of genes potentially controlling parasite-specific organelles such as dense granules, micronemes and major surface proteins were absent (Table S2). Thus, TgNF3 is probably important to nuclear/nucleolar functions linked to transcription and translation of genes involved parasite metabolism.

### *In vivo* binding of TgNF3 to ENO1 and 18S rDNA promoters is pertinent to stage-specific gene silencing in *T. gondii*

**Figure 15:**
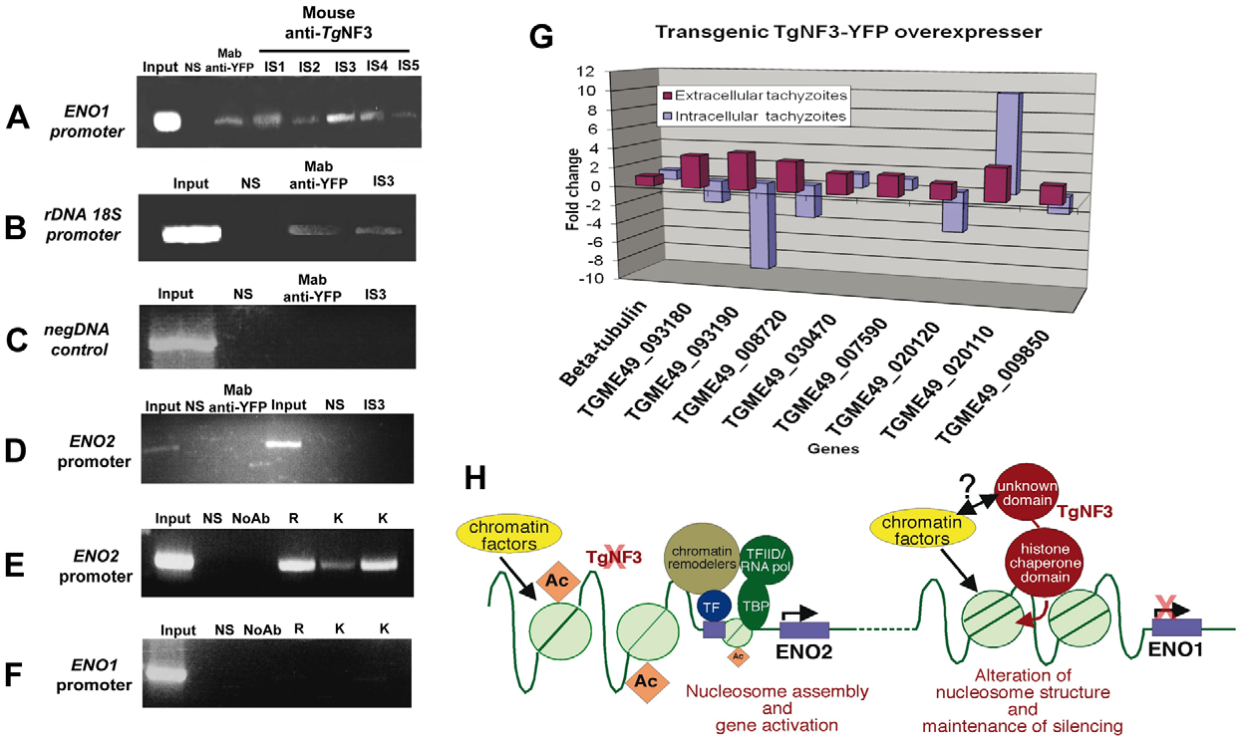
Chromatin immunoprecipitation (ChIP) analyses of TgNF3 binding sites *in vivo*. A) The anti-TgNF3 serum from five mice immunized with the recombinant TgNF3 protein was individually processed for ChIP experiments using tachyzoites of wild type 76K strain. Note that the stage-specific ENO1 promoter was specifically precipitated using either the anti-TgNF3 sera (IS1 to IS5) or the monoclonal anti-GFP antibody from chromatin extracts of wild type parasite and tachyzoites ectopically expressing TgNF3-YFP. The irrelevant naive mouse serum (NS), which was used as control did not immunoprecipitate ENO1 promoter. B) Both polyclonal antibody anti-TgNF3 (IS3 serum with the strongest signal, see panel A) and the monoclonal antibody anti-TgNF3 specifically immunoprecipitated ribosomal DNA 18S promoter using chromatin extracts from tachyzoites of wild type 76K strain and transgenic TgNF3-YFP expressers, respectively. C) A chromatin sample with an irrelevant genomic DNA corresponding to the open reading frame (ORF) of ENO1 used as negative control. D) No chromatin was immunoprecipitated when the tachyzoite-active ENO2 promoter was probed after ChIP assays using anti-GFP and anti-TgNF3 antibodies (IS3 serum). E) Chromatin samples corresponding to the tachyzoite-active ENO2 promoter were specifically amplified when the same three distinct epigenetic histone marks were tested using ChIP assays. F) No significant chromatin samples corresponding to bradyzoite-silent ENO1 promoter was immunoprecipitated using three epigenetic (acetylated and methylated) histone marks. The input corresponds to total chromatin extracts, which have not been immunoprecipated by antibodies but were directly PCR amplified by the same set of primers tested. G) Validation of the ChIP-seq data by qRT-PCR. Changes in gene expression were confirmed by qRT-PCR analysis for several genes that were upregulated (eight genes) in the extracellular tachyzoites or downregulated (five genes) in intracellular tachyzoites of TgNF3-YFP overexpressers. These experiments have been repeated twice with triplicate samples. H) Model of TgNF3 functions during promoter activity in *T. gondii*. TgNF3 binds to tachyzoite-silent ENO1 promoter whose core histones are not acetylated whereas the tachyzoite-active ENO2 promoter with epigenetic active marks through acetylated core histones does not interact with nuclear TgNF3. We discovered that TgNF3 can also positively or negatively regulate the expression of numerous nucleolar and nuclear genes according to the extracellular or intracellular niche of the parasite. We propose that TgNF3 may be a *T. gondii* nuclear chaperone involved in alterations in chromatin structure and function. This supports the notion that changes in chromatin structure and function may link the nuclear TgNF3 functions to the control of gene regulation in *T. gondii*.

Having observed that TgNF3 is probably involved in functions related to cellular metabolism and protein synthesis through control of translation, we decided to validate *in vivo* the binding of TgNF3 to two gene promoters, which are markers of nucleomorph and nucleolus, ENO1 and ribosomal DNA 18S. To address this, polyclonal antibodies specific TgNF3 and monoclonal anti-GFP antibody were used for chromatin immunoprecipitation using wild type *T. gondii* 76K and TgNF3-YFP transgenic parasites. Figure 15 shows that all anti-TgNF3 polyclonal antibodies individually collected from five immunized mice (designated immune sera IS1 to IS5) can positively pull down the ENO1 promoter from the chromatin extract of wild type tachyzoites (Figure 15A). Moreover, the monoclonal anti-GFP antibody immunoprecipitated the ENO1 promoter from the chromatin extracts of transgenic tachyzoites ectopically expressing TgNF3-YFP (Figure 15A). We next showed that the strongest positive polyclonal anti-TgNF3 antibody (IS3) and the anti-GFP monoclonal antibody also specifically pulled down the ribosomal DNA 18S chromatin *in vivo* (Figure 15B). A pool of pre-immune sera (lanes labelled NS on the top) is not able to immunoprecipitate chromatin DNA of the ENO1 promoter and 18S rDNA in both independent experiments described in Figure 15A and 15B, and irrelevant DNA encompassing the coding region of ENO1 can not be precipitated using both anti-GFP and anti-TgNF3 antibodies (Figure 15C). Neither anti-GFP, nor anti-TgNF3 can immunoprecipitate the ENO2 promoter that is active in the virulent tachyzoites using ChIP assays performed on the chromatin extracts from tachyzoites ectopically expressing TgNF3-YFP and parental parasites, respectively (Figure 15D). This indicates that TgNF3 is capable of binding to ENO1 promoter that is silent in the tachyzoites [31]. Indeed, we confirmed the silent status of ENO1 promoter in tachyzoites by probing this promoter with three distinct epigenetic histone marks such as acetylated and methylated histones [7], [8]. The data showed that these three epigenetic histone marks are absent on ENO1 promoter, which is consistent with this promoter being silent in tachyzoites (Figure 15F). In contrast, the ENO2 promoter is readily modified by all three acetylated histone marks (Figure 15E), which confirmed data previously reported for active promoters [7], [8]. Furthermore, we have used quantitative reverse-transcriptase PCR (qRT-PCR) to validate the expression profiles of eight genes identified by ChIP-seq, including the gene promoters shown in Figure 14 (Table S2 and Figure 14). For qRT-PCR analysis, RNA was purified from extracellular (48 h post-infection) and intracellular (24 h post-infection) tachyzoites and mRNA levels of both wild type and ectopic TgNF3-YFP over-expressing tachyzoites were compared. The results of the qRT-PCR revealed that eight genes tested were positively regulated in the extracellular tachyzoites as the RNA levels of these genes were 2- to 4-fold higher in the TgNF3 overexpressers than that of the wild type tachyzoites (Figure 15G). As a control, the levels of the housekeeping gene, β-tubulin was unchanged. In sharp contrast, we noticed that four up-regulated genes in the extracellular tachyzoites (TGME49_093180 (NADP-specific glutamate dehydrogenase, putative), TGME49_093190 (endonuclease/exonuclease/phosphatase domain-containing protein), TGME49_008720 (phosphatase, putative) and TGME49_020120 (hypothetical protein)), with greater hits of promoter binding by TgNF3 in ChIP-seq experiments, were also negatively regulated in the intracellular tachyzoites, as the levels of their mRNA in TgNF3 overexpressers were 2- to 10-fold less than that of wild type tachyzoites (Figure 15G). These data suggest that TgNF3 can either up-regulate or down-regulate gene expression and this depends principally on the extracellular to intracellular status of the parasite. Taken together, we propose a model for TgNF3 function (Figure 15H), which we define as a truly chromosome-associated factor that is probably involved in gene regulation, either repression or activation depending on its interacting partners, probably the promoter context and extracellular and intracellular niche of the parasite. The molecular mechanisms underlying its activity could involve modulation of nucleosome assembly and/or disassembly.

## Discussion

In this study, we sought novel nuclear factors that bind to a parasite stage-specific promoter, because almost nothing is known about transcription factors or other nuclear components involved in *T. gondii* gene regulation. Since it is very difficult to obtain enough nuclear protein from the persistent and dormant bradyzoites, we focused our efforts on the characterization of nuclear factors that bind to the *ENO1* promoter, which is transcriptionally silent in the rapidly replicating tachyzoites. Several factors isolated from nuclear extracts of *T. gondii* tachyzoites have either putative DNA-binding motifs and/or putative nuclear localization sequences. Consistent with the *ENO1* gene being transcriptionally silent in tachyzoites, two candidate nuclear factors appear to be homologues of the chromatin associated protein called Alba. This factor is known to be involved in transcription repression, a process requiring Sir2 and acetylation/deacetylation of a specific lysine residue for DNA-binding in Archaea [51]. Another candidate displays high sequence similarity in its N-terminal domain to yeast nuclear FK506-binding protein (FKBP), whose translocation into the nucleolus is associated with the transcriptional silencing of ribosomal RNA genes [33]. The identification in *T. gondii* of proteins sharing similarities with yeast and archaeal proteins, known as transcriptional repressors, is in good agreement with the silencing of the *ENO1* promoter in the rapidly replicating tachzyoites. We have validated that these candidate factors are capable of entering the nucleus of *T. gondii* tachyzoites when fused to either YFP, or HA-FLAG tags, or revealed by specific antibodies. Furthermore, we demonstrated that TgNF7 physically interacts with the GGGGG motif present in the bradyzoite silenced ENO1 promoter. It is interesting to note that this GGGGG motif is the homologue of the yeast stress-responsive element (STRE) that we have previously reported to specifically interact with *T. gondii* nuclear extract [31]. Here, we report that TgNF7 displays dual cytoplasm/nuclear localization and is a genuine binding factor for the GGGGG motif in the ENO1 promoter. In addition, we showed that TgNF3, a homologue of the yeast nuclear FKBP39, is largely present in the parasite nucleolus, even if the factor can also be detected in the nucleoplasm, the other nuclear compartment surrounding the nucleolus. This dual nucleolar/nucleoplasm location of TgNF3 is also consistent with the behaviour of yeast FKBP39, which displays a similar dual sub-nuclear distribution [33], suggesting that these two factors may share similar nuclear functions. The *Saccharomyces pombe* SpFKBP39 is a novel chromatin-modulating factor that acts as a histone chaperone regulating rDNA silencing. Furthermore, SpFKBP39 is a bi-partite protein with its N-terminal domain having the chaperone activity required in nucleosome assembly and disassembly and this involves interactions with chromatin-remodelling factors and chromatin-modifying enzymes. Our bioinformatics analyses provide evidence that the N-terminal domain of TgNF3 belongs to the HD2/nuclear FKBP family, including nuclear FKBP factors previously found only in yeasts and insects, plant-specific histone deacetylase 2 (HD2) and proteins from parasitic apicomplexans such as *Plasmodium falciparum*, the causative agent of malaria [39], [40]. Whether TgNF3 has a deacetylase activity towards histones awaits further investigation. It worth noting that the genome-wide analyses of gene promoter occupancy by TgNF3 also identified several histone deacetylases, suggesting these deacetylases may be TgNF3 partners, given that this enzymatic activity is likely absent from the factor. In fact, we were unable to demonstrate any histone deacetylase activities for TgNF3 using the recombinant protein and commercially available kits. In contrast to its yeast homologues nuclear FKBP and plant HD2, TgNF3 also lacks the propyl peptidyl isomerase domain at the C-terminus. Instead, a divergent C-terminal domain with no predicted functions is present in TgNF3 protein, supporting the notion that this novel C-terminal domain may be involved in protein-protein interactions, for instance, an interaction with histone deacetylases required to ensure transcriptional repression through its chaperone functions involved in nucleosome activities [33]. Moreover, the nuclear partners that interact with the yeast homologue FKBP (SpFKBP39p) have not been identified to date. Therefore, the transgenic parasites expressing HA-FLAG described in this study may be a useful tool for the identification of nuclear/nucleolar partners that interact with TgNF3 in *T. gondii*. This will also shed light on the regulatory networks that involve TgNF3 functions and will also define the precise biological roles of the two distinct domains of TgNF3 in binding to chromatin remodelling components and/or to transcription factors. Nevertheless, we have been able to demonstrate that TgNF3 can specifically bind to both mammalian core histones containing H3, H4 and H2A/H2B complexes and to parasite nucleosome-associated histones. Consistent with its presence in the nucleolus, TgNF3 also specifically interacts *in vivo* to the 18S ribosomal RNA gene promoter of *T. gondii*, which is consistent to the nucleolar functions previously described for its homologue FKBP in yeast [33]. In contrast, TgNF3 is also significantly present in other areas of the nucleus and binds to numerous gene promoters that cannot be considered as genuine nucleolar resident factors. It is tempting to hypothesize that the C-terminal divergent domain of TgNF3 may be involved in other transcriptional activities distinct from ribosomal RNA gene promoter regulation. Knockout mutants complemented with truncation versions of TgNF3 will be of great help to decipher the precise functions of the C- and N-terminal domain of the protein. Ectopic expression of TgNF3 in transgenic parasite lines described here has, however, demonstrated its direct role as a positive or negative regulator of transcriptional activity of genes present in both the nucleolus and nucleoplasm of *T. gondii*. The generation of TgNF3 knock out mutants combined with microarray analysis will also be useful to clarify the transcriptional functions of this novel factor. Unfortunately, our attempts to knockout the *TgNF3* gene in *T. gondii* have failed so far, suggesting that the *TgNF3* gene codes for an essential protein. The new methods for conditional knockdown described by Hudson *et al* [52], the alternative replacement of endogenous gene promoter by homologous anhydrotetracyclin inducible promoter [53] or fusion to the ligand-controlled destabilization domain (DD-FKBP) to achieve conditional expression [54] might be helpful in the future to understand how TgNF3 controls gene expression and parasite virulence.

Ectopic expression of TgNF3-YFP did not appear to be toxic, even though the size of parasite nucleoli was increased in size. The YFP-tagged TgNF3 expression allowed us to investigate the dynamics of the nucleolus in *T. gondii* during the parasite development inside the host cell, an issue that has not been investigated previously. Time-lapse images and movies consistently showed a dynamic nucleolar biogenesis with the appearance of two smaller sized nucleoli, or one small nucleolus close to a larger one during parasite replication, suggesting either fragmentation and/or *de novo* synthesis of the parasite nucleolus. TgNF3-YFP, ectopically expressed in tachyzoites, induced a severe attenuation of parasite virulence *in vivo*. However, the molecular mechanisms involved in the virulence attenuation of over-expressing transgenic parasites remain to be determined. We believe that the identification of genes transcriptionally regulated by TgNF3, using comparative microarray studies between wild type parasites and the transgenic over-expressers, will likely reveal the putative virulence factors that are controlled by TgNF3. Interestingly, the presence of abundant levels of both TgNF3-YFP and wild type TgNF3 protein only in the cytoplasm of avirulent bradyzoites strongly suggests that this factor is exclusively required for nuclear functions in the virulent tachyzoites. We noticed that the decrease of TgNF3 signal in the cytoplasm correlates with a profound size reduction and degeneration in some cases of bradyzoite nuclei. The exclusive location of TgNF3 in the cytoplasm of dormant bradyzoites also suggests that nucleolar functions may be important for the replication of *T. gondii* tachyzoites. Because TgNF3 and the other nuclear factors were identified through their binding to a transcriptionally silent gene in tachyzoites, we conclude that silencing of bradyzoite-specific *ENO1* gene and other genes may activate expression of tachyzoite-specific genes and promote the rapid parasite replication and growth *in vitro*. Furthermore, genome-wide analyses revealed that TgNF3 binds to numerous gene promoters mainly involved in cellular metabolism and nucleolar functions linked to translation such as ribosomal and protein synthesis. It is worth noting that TgNF3 is also substantially present outside the nucleolus, namely in the nucleoplasm, where it can interact with the bradyzoite-specific *ENO1* and other gene promoters controlling expression of cellular metabolism, translation and transcription. Since the *ENO1* gene is transcriptionally silent in tachyzoites and its promoter has been used to isolate TgNF3 and the other novel nuclear factors, an attractive model, which is schematically presented in Figure 15H, is that TgNF3 may be a *T. gondii* nuclear chaperone involved in altering chromatin structure and function [55]. TgNF3 could also affect protein translation and ribosomal synthesis, both of which are known to be involved in diverse cellular pathways and metabolism during growth of metazoan and protozoan species. Our findings that TgNF3 regulates ribosomal protein synthesis is consistent with recent descriptions of a pathway involved in the regulation of the cell cycle and ribosomal proteins, which is controlled by apetela2 (AP2) transcription factors in *T. gondii* [52], [56]. In addition to transcriptional regulation of nucleolar genes, as validated by ChIP-seq and qRT-PCR, we discovered that TgNF3 can positively or negatively regulate the expression of numerous genes present in other areas (the nucleoplasm) of the nucleus, and these genes are upregulated or downregulated according to the extracellular or intracellular niche of the parasite. This is in sharp contrast with the function of the yeast homologue FKBP (SpFKBP39p) where transcriptional repression of ribosomal DNA has only been described [33]. In conclusion, we propose that the nuclear functions of TgNF3 involve changes in chromatin structure and this may link TgNF3 activity to the control of the expression of genes that regulate parasite proliferation, virulence, as well as differentiation and cyst formation.

## Materials and Methods

### *In vitro* growth of *T. gondii* and nuclear extract preparation

Human foreskin fibroblasts (HFFs) were maintained in Dulbecco's modified Eagle's medium (DMEM, BioWhittaker, Verviers, Belgium) supplemented with 10% fetal calf serum, 2 mM glutamine (Sigma), and 0.05% gentamycin (Sigma). Tachyzoites from *T. gondii* 76K strain were grown in monolayers of HFF cells until they lysed the host cells spontaneously. Freed tachyzoites were harvested and purified using glass wool columns and 3-µm pore filters. Encysted bradyzoites were released from cysts purified from brains of mice chronically infected by *T. gondii* 76K strain using the percoll gradient method and 0.05% of pepsin/HCl, as previously described [23]. Nuclear extracts were obtained from about 4×10^10^ purified tachyzoites of *T. gondii* 76K strain, as previously described [31]. The parasite nuclear extracts were aliquoted and stored at −80°C before use.

### Promoter DNA-biotinylation and affinity purification

The nucleotide sequence corresponding to ENO1 promoter was amplified by PCR and cloned into BlueScript. The 200 µg DNA fragment was sequentially digested with EcoRI and HincII enzymes at 37°C for 2 hours. The released DNA fragment was purified by GeneClean after electrophoresis on agarose gel. The purified DNA fragment was biotinylated using Klenow enzyme, 1 mM of dATP, 1 mM dCTP, 1 mM dGTP and 8 µl of dUTP 16-Biotin at 37°C for 2 h. The biotinylated DNA was purified by High Pack PCR kit (Roche). Total nuclear extract of about 17 mg of proteins was diluted with 7 ml of binding buffer (12% glycerol, 1.5 mM MgCl_2_, 12 mM Hepes pH 7.9, 4 mM Tris.HCl pH 7.9, 60 mM KCl, 1 mM EDTA, 1 mM DTT) and incubated with 800 µl of Streptavidine-agarose at 4°C for 1 h. After centrifugation, the supernatant was mixed with 350 µl of biotinylated DNA and the mixture was incubated at room temperature for 30 minutes. The DNA-protein complexes were isolated by affinity purification using 800 µl of Streptavidine-agarose for 4 h at 4°C. The beads were washed with binding buffer 10 times and eluted with 3 ml of elution buffer (12% glycerol, 20 mM Tris.HCl pH 6.8, 1 M KCl, 5 mM MgCl_2_, 1 mM EDTA, 1 mM DTT) at 4°C for 40 min. The eluted proteins were dialyzed and concentrated by centrifugation using Centricon tubes (MWCO10, Millipore) and analyzed by SDS-PAGE followed by silver staining.

### Sample preparation for proteomic analysis

Silver stained gels were systematically cut into slices, and in-gel digestion was performed with an automated protein digestion system, MassPREP Station (Waters, Milford, MA). The gel slices were washed three times in a mixture containing 25 mM NH_4_HCO_3_: CH_3_CN (1∶1, v/v). The cysteine residues were reduced with 50 µL of 10 mM dithiothreitol at 57°C and alkylated with 50 µL of 55 mM iodoacetamide. After dehydration with acetonitrile, the proteins were cleaved in gel with 40 µL of 12.5 ng/µL of modified porcine trypsin (Promega, Madison, WI, USA) in 25 mM NH_4_HCO_3_ at 37°C for 4 hours. The tryptic peptides were extracted with 60% acetonitrile in 0.1% formic acid.

### Mass spectrometry analysis

NanoLC-MS/MS analyses were performed using an Agilent 1100 series nanoHPLC-Chip/MS system (Agilent Technologies, Palo Alto, USA) coupled to a HCT Plus ion trap (Bruker Daltonics, Bremen, Germany). The chip contained a Zorbax 300SB-C18 column (43 mm×75 µm, 5 µm particle size) and a Zorbax 300SB-C18 enrichment column (40 nL, 5 µm particle size). The solvent system consisted of 2% acetonitrile, 0.1% formic acid in water (solvent A) and 2% water, 0.1% formic acid in acetonitrile (solvent B). 2 µL of each sample was loaded into the enrichment column at a flow rate set to 3.75 µL/min with solvent A. Elution was performed at a flow rate of 300 nl/min with a 8–40% linear gradient (solvent B) in 7 minutes followed by a 3 min stage at 70% of solvent B before reconditioning the column at 92% of solvent A. The system was fully controlled by ChemStation Rev B.01.03.SRI (Agilent Technologies). The MS instrument was operated with the following settings: source temperature was set to 320°C while cone gas flow was at 3 l/min. The capillary voltage was optimized to −1850 V. The MS spectra were acquired in the positive ion mode on the mass range 250 to 2500 m/z using the standard enhanced resolution mode at a scan rate of 8.100 m/z/s. The Ion Charge Control was fixed at 100000 with a maximum accumulation time of 200 ms and the number of averages was set to 4. For tandem MS experiments, the system was operated with automatic switching between MS and MS/MS modes. The 3 most abundant peptides were selected on each MS spectrum for further isolation and fragmentation with a preference for doubly charged ions (absolute threshold of 2000 and a relative of 5%). Ions were excluded after the acquisition of 2 MS/MS spectra and the exclusion was released after one minute. The Smart Parameters Setting option was used for the selected precursor ions. The MS/MS spectra were acquired on the mass range 50 to 2800 m/z using the ultrascan resolution mode at a scan rate of 26.000 m/z/s. The Ion Charge Control was fixed at 300000 and 6 scans were averaged to obtain a MS/MS spectrum. The MS/MS fragmentation amplitude was set to 1.5 V. The system was fully controlled by the Esquire Control 5.3 Build 11.0 software (Bruker Daltonics). Mass data collected during the nanoLC-MS/MS analyses were processed and converted into *.mgf files using the DataAnalysis 3.3 Build 146 software (Bruker Daltonics).

### Protein identification

The MS/MS data were analyzed using the MASCOT 2.2.0. algorithm (Matrix Science, London, UK) and Open Mass Spectrometry Search Algorithm (OMSSA) [57] for search against an in-house generated protein database [58], which is composed of protein sequences of *Apicomplexa* downloaded from http://www.ncbi.nlm.nih.gov/sites/entrez (on may 25, 2009) concatenated with reversed copies of all sequences (total 307386 entries). Searches were performed with a mass tolerance of 0.25 Da in both MS and MS/MS mode and with the following parameters: full trypsin specificity with one missed cleavage, methionine oxidation, protein amino-terminal acetylation and cystein carbamidomethylation. The Mascot and OMSSA results were loaded into the Scaffold software (Proteome Software, Portland, USA). To minimize false positive identifications, results were subjected to very stringent filtering criteria as follows. For the identification of proteins with two peptides or more, a Mascot ion score above the identity score or an OMSSA E-value below −log(e^2^) was required. The target-decoy database search allowed us to control and estimate the false positive identification rate of our study [59], [60]. Thus, the final catalogue of proteins presents an estimated false positive rate below 1%.

### Plasmid construction, transfection and transformation

The full-length cDNAs corresponding to several proteins identified by proteomic analysis were fused to YFP using pTub1-YFP and pGRA1-HAFLAG plasmids, which were generously provided by Dr Dzierszinski F & Roos DS (University of Pennsylvania, USA) and Dr Hakimi MA (University of Grenoble, France), respectively. The expression vectors were transiently transfected in the tachyzoites and processed for IFA after 24 h post-infection. For stable transformation of *T. gondii*, Tub1-TgNF3-YFP plasmid was transfected in 10^7^ tachzyoites of *T. gondii* 76K strain and grown in the presence of 20 µg/ml of chloramphenicol until emerged resistant and stable parasites were cloned. For recombinant protein expression, the full-length cDNA of TgNF3 was cloned in pGEX and used to transform *E. coli*. The recombinant TgNF3 was induced by IPTG and the soluble recombinant protein was purified using GST column according to the manufacturer's recommendations. A lot of five mice were immunized by 100 µg of recombinant protein per mouse in the presence of complete Freund adjuvant (Sigma). After two boosts with 50 µg of recombinant TgNF3 per mouse in the presence of incomplete adjuvant (Sigma), the sera were tested by Western blots before a final challenge.

### Quantitative real-time RT-PCR, GST pull down assays and Western blotting

For qRT-PCR, the RNA from 10^8^ tachyzoites purified from infected HFF cells and 10^6^ bradyzoites isolated from 2,000 cysts of brains of chronically infected mice were used. The total RNA were reverse transcribed for one hour at 42°C in a buffer containing 1 M oligo(dT)_18_ primer, 2 mM dNTPs, 40 U of rRNasin (Promega) and 25 U of AMV reverse transcriptase (Roche). The primers used for RT-PCR were as follows: *T. gondii* β-tubulin gene, forward, 5′-TCCTCGCTCCTTTTGATGTC-3′, and reverse, 5′- ATTGGAGACAATCCCGTCAG-3′; and *T. gondii* TgNF3, forward, 5′-CACCACGCCAGAAAGCACATC-3′, reverse, 5-CCTCGTCGTCCTCATCGTCAT-3′. We checked that each primer pair amplified a single fragment, identified as a single band in acrylamide gel and as a single peak within the qPCR dissociation curve. The primer pairs displayed an amplification efficiency of greater than 90%. The qPCR was then performed with the Maxima TM SYBR Green qPCR Master Mix Kit (Fermentas) and using the Mx3005P TM real-time PCR System (Stratagene). ROX Solution was used as a passive reference for all analyses and the qPCR was repeated three times, each time in duplicate. Gene expression in tachyzoite is represented as a percentage of bradyzoite gene expression after normalisation.

For GST-pull down experiments, 100 µg of purified recombinant GST-TgNF3 and GST alone were resuspended in binding buffer (20 mM Hepes pH 7. 9, 150 mM NaCl, 0.5 mM EDTA, 10% glycerol, 0.1% Tween 20 and a cocktail of protease inhibitor and PMSF) and incubated with 100 µl of glutathione-Sepharose 4B (Amersham Pharmacia Biotech) at 4°C overnight. The beads were washed three times with the binding buffer without protease inhibitors. Five µg of purified core histones from HeLa cells (Upstate, Millipore) or purified nucleosomes from isolated nuclei of *T. gondii* [7], kindly provided by Dr Hakimi MA, were resuspended with the binding buffer and added to 10 µl of GST-TgNF3, GST and to anti-TgNF3 antibodies coupled to Sepharose beads. For competition assays, 5 µg of core histones or purified *T. gondii* nucleosomes were incubated with recombinant non-fusion TgNF3 or GST for one hour at room temperature prior pull down assays. After incubation at room temperature and four washes, pulled down proteins were eluted by boiling in 25 µl of Laemmli sample buffer and analyzed by SDS-PAGE for silver staining or Western blots using rabbit polyclonal anti-Histone H3 antibody (Upstate, Amersham). Total protein extracts from *T. gondii* tachyzoites (5×10^6^ tachyzoites per lane) or purified *T. gondii* nucleosomes were also boiled in Laemmli's buffer, separated by SDS-PAGE and transferred to Hybond ECL nitrocellulose (Amersham). Immunoblots were carried out using murine immune sera, non-immune sera as control, monoclonal antibodies specific to GFP (Clontech) or with mouse polyclonal antibody raised against recombinant anti-TgNF3. The blots were incubated with peroxidase conjugated secondary antibodies followed by chemiluminescence detection.

### Electron microscopy and immunofluorescence assays

For immuno-electron microscopy, the intracellular transgenic tachyzoites expressing TgNF3-YFP grown in HFF cells were fixed overnight at 4°C in 8% paraformaldehyde in PBS buffer, thoroughly washed in the same buffer and infused in sucrose 2.3 M containing 20% polyvinyl pyrrolidone 10000 in phosphate buffer 0.1 M. The pellets were mounted on ultracryotome supports and rapidly frozen in melting nitrogen. Ultrathin sections of about 90–100 nm were obtained using Reichert UltraCut E ultramicrotome equipped with a FC4 device. Before mounting in methyl cellulose, sections were incubated in blocking medium (0.05 M glycine, 5% fish gelatine in 0.1 M PBS buffer) for 30 min. The grids were incubated with the monoclonal antibody anti-GFP for 1 hour at 37°C or overnight at 4°C. After washing, sections were incubated at room temperature for 30 min in the corresponding secondary gold conjugates (Jackson ImmunoResearch Laboratories Inc.) diluted in the same buffer. Following a final thorough wash in PBS alone, the grids were fixed in 4% paraformaldehyde for 10 min at room temperature and washed in water. After staining with 0.5% uranyl acetate in 1.5% methyl cellulose, sections were observed on a Hitachi H600 transmission electron microscope at 75 kV accelerating voltage.

For immunofluorescence assays (IFA), intracellular tachyzoites were fixed with 4% paraformaldehyde in PBS for 15 minutes on ice, followed by two PBS washes and dried on Teflon slides. Intracellular parasites were permeabilized with 0.1% Triton X-100 in PBS containing 0.1% glycine for 10 minutes at room temperature. Samples were blocked with 3% BSA in the same buffer and mice immune sera diluted at 1∶1000 were added on parasites in the same buffer for one hour at 37°C. Rabbit secondary antibody coupled to Alexa-488 (Molecular Probes) diluted at 1∶1000 was added in addition to DAPI for nucleus staining. For co-localization assay, the rabbit anti-enolase serum and the goat secondary antibody coupled to Alexa-594 (Molecular Probes) were used at the same dilution. Fluorescence was visualized with a ZEISS Axiophot microscope.

### Imaging by confocal microscopy

Confocal imaging was performed with a LSM710 microscope (Zeiss) and a Plan Apochromat objective (Plan-Apochromat 63×/1.40 Oil DIC M27, Zeiss). The associated software (Zen 2008) enabled the adjustment of acquisition parameters. YFP was excited at 514 nm and its fluorescence emission was collected from 520 to 620 nm. The Alexa-488 signal was excited at 488 nm and emission was collected from 500 to 580 nm. Nuclear DAPI signal were excited at 405 nm and emission was collected from 410 to 500 nm. Fluorescent signals were collected sequentially, with a 4 lines average, a zoom factor (varying between 2 and 4) and resulting images are 512×512 pixels in size, and 8 bits in resolution (256 gray levels). By setting the photomultiplier tubes and the pinhole size (1 AU) correctly, there was no signal bleed-through. The images were treated with ImageJ (NIH). Z-stack acquisitions enabled to visualize the 3D localization of fluorescent signals. Rotation (360° around the y axis) movies were created with the Zen software, at a rate of 5 frames per second.

### Live video microscopy

Images were obtained from an AxioObserver Z1 (Zeiss), equipped with a regulation chamber for temperature and CO_2_, through a Plan Apochromat objective (Plan-Apochromat 100×/1.46 Oil, Zeiss). Samples were excited with a Colibri source (Zeiss) and fluorescence signal was collected with an Axio mRm Camera (Zeiss), through different filters sets (Filter Set 38HE, 60HE, Zeiss). Experiments were performed by exposing the sample at a rate of one image every 30 minutes during 72 h. Z-stack acquisitions were performed, and resulting images, which correspond to the projections of these stacks, were shown. The images were treated using ImageJ (NIH).

### Chromatin immunoprecipitation

ChIP was performed as described [7] with slight modifications. Briefly, chromatin from extracellular or intracellular parasites grown in HFF cells was cross-linked for 10 min with 1% formaldehyde at room temperature and purified. Chromatin extract was obtained after sonication yielding fragments of 500–1,000 bp. Immunoprecipitations were performed with the monoclonal antibody anti-GFP or the mouse serum anti-TgNF3 at 4°C overnight and washed as described [7]. DNA was then subjected to proteinase K digestion for 2 h and purified using the Qiagen PCR purification kit (http://www.qiagen.com). As a negative control, pre-immune sera were used. For PCR, specific primers of ENO1 gene promoter, forward 5′-ATGTGCTGCTGGTTTTTGTTTC-3′, reverse, 5′-TTAAGGCGTCCGCAAGACTAGTG-3′ and ribosomal RNA 18S gene promoter, forward, 5′-GGGGTGGTGGATGGGGACGGGCGC-3′, reverse 5′-GCCCGTTCCTTGACCCCGCTGCC-3′ were used. ChIP products amplified by PCR using these specific primers were electrophoresed on agarose gels, stained with ethidium bromide, and photographed using UV-light scanner.

### Chromatin immunoprecipation and high-throughput sequencing

Chromatin immunoprecipitated by anti-TgNF3 as above was amplified using GenomePlex Amplification of ChIP DNA kit (Sigma-Aldrich). The amplified ChIP products were electrophoresed on agarose gels and stained with ethidium bromide. The fragments of DNA length up to 500 bp were purified and processed for high-throughput sequencing (GenoScreen, Pasteur Institute of Lille). The GsFLX bead adaptors and specific tag (MID) were introduced to the flanking 5′ and 3′ end of each purified DNA sample according to manufacturer's instructions. The ChIP-seq GsFLX libraries were controlled on a BioAnalyzer 2100 using Agilent RNA 6000 Pico methods and quantified by Quant-iT TM RiboGreen (Invitrogen). Equimolar librairies were mixed, fixed on beads and amplified by GS FLX Titanium emPCR Kit (454 Life Sciences, Roche Diagnostics). The amplified beads were purified, enriched, counted using Beckman Coulter Z1 and deposited on the GS FLX Titanium PicoTiterPlate (454 Life Sciences, Roche Diagnostics). The pyrosequencing reaction was performed using GS FLX Titanium Sequencing Kit (454 Life Sciences, Roche Diagnostics) on Genome Sequencer FLX Instrument (454 Life Sciences, Roche Diagnostics).

### Bioinformatics

Sequence similarity searches were performed using PSI-BLAST at the National Center for Biological Information (NCBI, nonredundant (nr) database, default parameters). Fold recognition was performed using Phyre [61]. Hydrophobic Cluster Analysis (HCA, [39]) was used to analyze protein domain architecture and refine sequence alignments. For ChIP-seq bioinformatics analyses, the GSMapper software v 2.3 (Roche) was used to align reads for each sample using the updated *T. gondii* ME49 genome databases downloaded from http://www.toxodb.org v 6.0 of 15 July 2010. The sequences of a minimum overlapping length of 40 nucleotides with at least 90% identity were only considered for further analyses. The ChIP-seq data specific to TgNF3 was subtracted from non-specific sequences obtained by the pre-immune sera used as negative control and data collected for specific sequences immunoprecipitated by anti-TgNF3 antibodies were grouped into 4 sub-groups of associated contigs. SignalMap software was used to schematically represent the number of reads per contig and their corresponding positions, which allowed to visualizing putative gene promoters occupancy by TgNF3 defined by ChIP-seq for all chromosomes of *T. gondii*.

### Experimental infection in mice and ethics statement

All animal experiments were performed following the guidelines of the Pasteur Institute Pasteur of Lille animal study board, which conforms to the **A**msterdam **P**rotocol on animal protection and welfare, and **D**irective 86/609/EEC on the **P**rotection of **A**nimals **U**sed for **E**xperimental and **O**ther **S**cientific **P**urposes, updated in the **C**ouncil of **E**urope's **A**ppendix A (http://conventions.coe.int/Treaty/EN/Treaties/PDF/123-Arev.pdf). The animal work also complied with the French law (n°87-848 dated 19-10-1987) and the **E**uropean **C**ommunities **A**mendment of **C**ruelty to **A**nimals **A**ct 1976. All animals were fed with regular diet and all procedures were in accordance with national regulations on animal experimentation and welfare authorized by the French Ministry of Agriculture and Veterinary committee (Permit number: 59-009145). The Pasteur Institute of Lille and the CNRS Committee on the Ethics of Animal Experiments specifically approved this study. Purified tachyzoites from the parental and transgenic ectopically expressing TgNF3-YFP were inoculated into group of 10 female 6–8-week-old BALB/c or CBA/J mice at 10^4^ per mouse (otherwise stated) and monitored until death or survival for 2–3 months. To check that surviving mice were infected and the immune response had developed in the infected mice, the serum of each mouse was tested against the total extract antigens prepared from the parental parasites using Western blots. To assess whether the primary infection of mice with tachyzoites ectopically expressing TgNF3-YFP conferred protection against the parental 76K and the highly virulent RH strains, infected mice that survived after 42 days post-inoculation were challenged with lethal parasite doses. *In vivo* cyst formation was determined by harvesting mouse brain at 6–8 weeks after infection. Cysts were purified using Percoll gradients, washed with PBS, and observed by inverted phase and confocal microscopy.

### Statistical analysis

Statistical differences between groups of mice used in this study were evaluated by the Student's t-test. The Mann-Whitney test was also used for counting intracellular growth of TgNF3-YFP and wild type tachyzoites after colorimetric staining and microscopic observation, and also for the test for mice survival curves.

## Supporting Information

Figure S1 Nucleotide sequence of the promoter of *T. gondii* bradyzoite-specific ENO1 gene used to purify novel nuclear factors after biotinylation and affinity purification. The same sequence has been used to generate biotinylated probe for electrophoretic mobility shift assays (EMSA). The 47-bp fragment binding to recombinant TgNF7 is shown in red and the GGGGG motif is in blue and underlined [31].
(0.29 MB TIF)

Figure S2 Genome-wide TgNF3 occupancy defined by ChIP-seq and bioinformatics analyses showing overall linear view of the 14 chromosomes of *T. gondii* and the different hits in gene promoters were shown as red vertical bars.
(1.46 MB TIF)

Table S1 Protein identified by mass spectrometry using *nano* LC-MS/MS. The proteins, whose nuclear localization has been validated by reverse genetics using a yellow fluorescence protein tagged version or specific polyclonal antibodies, were designated TgNF for *T. gondii* nuclear factors.
(0.06 MB PDF)

Table S2 Identification of *T. gondii* genes and promoters defined by genome-wide TgNF3 occupancy and ChIP-seq.
(0.10 MB PDF)

Video S1 3D-confocal reconstruction of TgNF3 signal in the wild type or parental parasites expressing native TgNF3 protein using specific polyclonal antibodies. Ten confocal images generated in increments of 0.6 µm.
(1.27 MB AVI)

Video S2 3D-confocal reconstruction of TgNF3-YFP signal in parasite over-expressing TgNF3 protein. Ten confocal images generated in increments of 0.8 µm.
(1.41 MB AVI)

Video S3 Video representing the displacement along the z-axis (confocal Z-stack acquisition) created with the Zen software, at a rate of 5 frames per second showing the presence of TgNF3-YFP in bradyzoites (green dots appearing during the movie) present inside the cyst whose wall was stained with *Dolichos biflorus* lectins. The video was generated with 41 images in increments of 0.45 µm.
(1.92 MB AVI)

Video S4 Video representing the displacement along the z-axis (confocal Z-stack acquisition) created with the Zen software as above and showing the presence of TgNF3-YFP in bradyzoites (green dots appearing during the movie) inside the cyst whose wall was not stained with *Dolichos biflorus* lectins. The video was generated with 24 images in increments of 0.45 µm.
(2.19 MB AVI)

Video S5 Video S5 3D-confocal reconstruction of panel 1 from Figure 12 showing that native TgNF3 signal is exclusively localized to the cytoplasm of the bradyzoites. The video was generated with 7 images in increments of 0.8 µm.
(2.03 MB AVI)

Video S6 Another example of 3D-confocal reconstruction of panel 2 from Figure 12 is shown. It also shows the presence of native TgNF3 signal only in cytoplasm of the bradyzoites. The video was generated with 7 images in increments of 0.8 µm.
(1.62 MB AVI)
