# Supplementary figures and images for: A Novel Toxoplasma gondii Nuclear Factor TgNF3 Is a Dynamic Chromatin-Associated Component, Modulator of Nucleolar Architecture and Parasite Virulence
Source: PLoS Pathog. 2011 Mar 31;7(3):e1001328. doi: 10.1371/journal.ppat.1001328 (PMC3068996; doi:10.1371/journal.ppat.1001328)

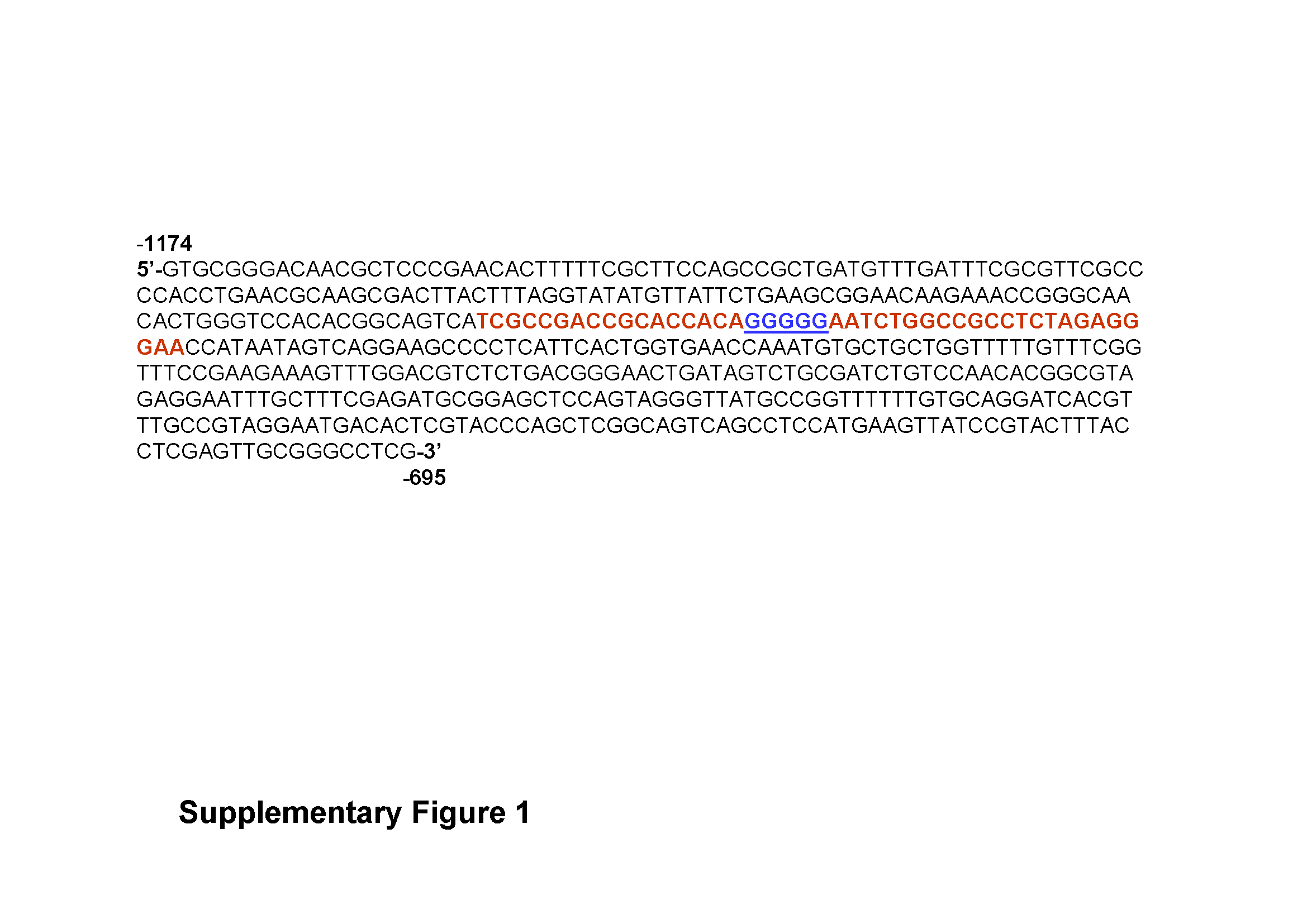

Supplement: Figure S1 — Nucleotide sequence of the promoter of T. gondii bradyzoite-specific ENO1 gene used to purify novel nuclear factors after biotinylation and affinity purification. The same sequence has been used to generate biotinylated probe for electrophoretic mobility shift assays (EMSA). The 47-bp fragment binding to recombinant TgNF7 is shown in red and the GGGGG motif is in blue and underlined [31]. (0.29 MB TIF) [file ppat.1001328.s001.tif]

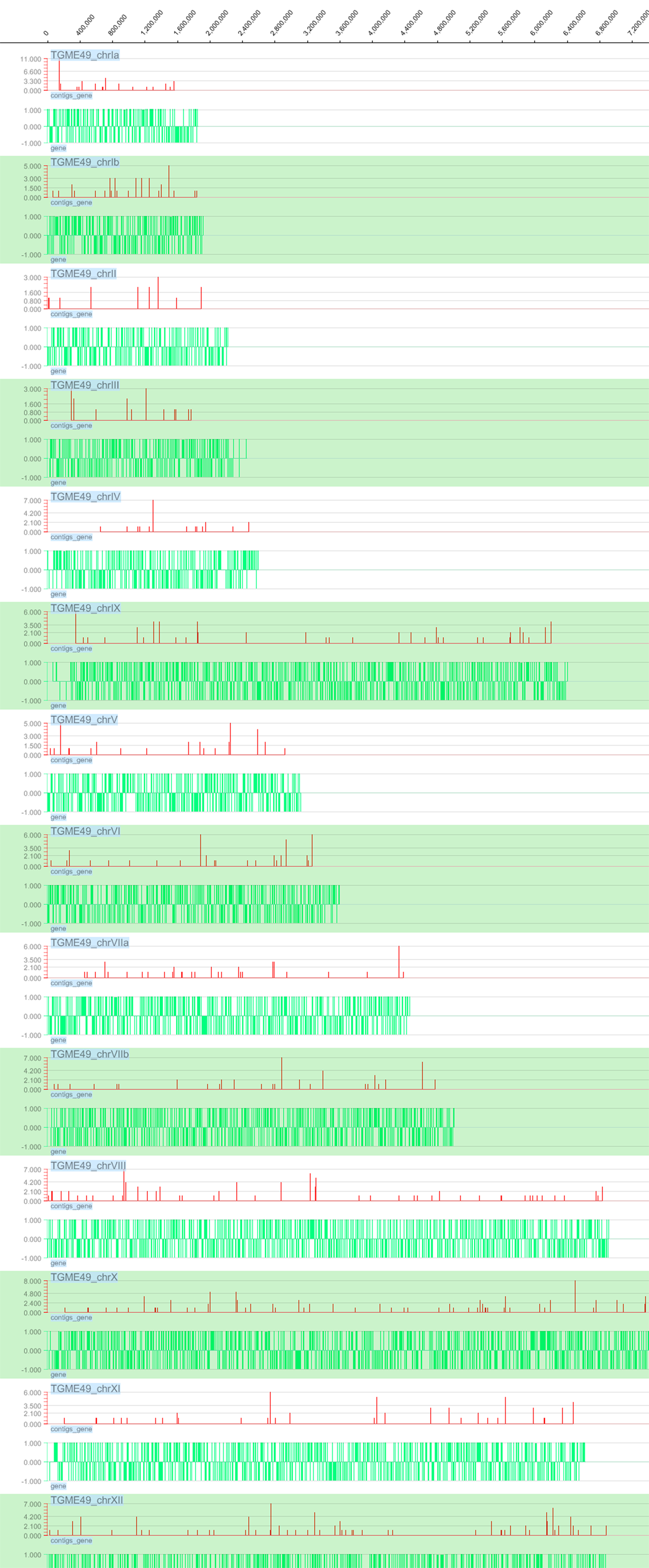

Supplement: Figure S2 — Genome-wide TgNF3 occupancy defined by ChIP-seq and bioinformatics analyses showing overall linear view of the 14 chromosomes of T. gondii and the different hits in gene promoters were shown as red vertical bars. (1.46 MB TIF) [file ppat.1001328.s002.tif]
