# Supplementary material for: A Novel Toxoplasma gondii Nuclear Factor TgNF3 Is a Dynamic Chromatin-Associated Component, Modulator of Nucleolar Architecture and Parasite Virulence
Source: PLoS Pathog. 2011 Mar 31;7(3):e1001328. doi: 10.1371/journal.ppat.1001328 (PMC3068996; doi:10.1371/journal.ppat.1001328)
Supplement: Table S1 — Protein identified by mass spectrometry using nano LC-MS/MS. The proteins, whose nuclear localization has been validated by reverse genetics using a yellow fluorescence protein tagged version or specific polyclonal antibodies, were designated TgNF for T. gondii nuclear factors. (0.06 MB PDF) [file ppat.1001328.s003.pdf]

Supplementary Table 1

T. gondii nuclear factors	Protein name	Protein accession number	Protein molecular weight (Da)	Number of unique peptides	Percentage sequence coverage	Peptide sequence	Nuclear localization using YFP tagging and/or specific antibodies	Slice number
**Nuclear functions**								
**TgNF1**	DEAD/DEAH box helicase, putative TGME49_036650 [Toxoplasma gondii ME49]	gi|211966692	59 951	3	7.5%	DMIGIAETGSGK	**YES**	**21**
						SGGFGGGPNALPLGSRPY		
						VTYLVMDEADR		
**TgNF2**	pinin/SDK/memA/ domain-containing protein TGME49_086790 [Toxoplasma gondii ME49]	gi|211966969	36 006	9	40.9%	AMCTELDTSEPR	**YES**	**16**
						ESSEVKAEGETVAAGEDER		
						LEEVSTELSNK		
						LEEVSTELSNKENELMR		
						MFGALAGHLQR		
						NMEVFIGTEAQPTLFWAPAK		
						QLLMEQSTDLAHK		
						RPPGELFSLDKPEASAPSLPQK		
						VTYSFEGEEFQVEK		
**TgNF3**	46 kDa FK506-binding nuclear protein, putative TGME49_060440 [Toxoplasma gondii ME49]	gi|211962881	33 952	7	21.9%	ANASTFDVSNGVCSLK	**YES**	**16**
						DKLEFTSLDLFLSTR		
						TPLATLGQK		
						TYSVCMLQK		
						TYVQVIQEGK		
						SALVDFLK		
						SALVDFLKK		
	SWIB/MDM2 domain-containing protein TGME49_073930 [Toxoplasma gondii ME49]	gi|211963658	27 990	3	12.2%	DQVSMFELNK		**1**
						HIWDYIK		
						MINADSTLRPLFQK		
	Gbp1p protein, putative TGME49_062620 [Toxoplasma gondii ME49]	gi|211963026	31 742	4	17.1%	ADVFEDFQGR		**9**
						ADVMTMPDGR		
						GCGIVEYTNVEDAQK		
						GVGTVLFSTPEGAQR		
	Nucleolar protein family A, putative TGME49_072010 [Toxoplasma gondii ME49]	gi|211963509	20 913	2	16.2%	EEIGKVDEILGPINEMLFSVR		**9**
						GTLSQQVPYFNGR		
	RRM domain-containing protein TGME49_036540 [Toxoplasma gondii ME49]	gi|211966681	53 421	3	8.8%	QEMEFAAQGGDPR		**17-20**
						TTGEAYVQLPDPGLR		
						TVMAAISGNTDSLDASR		

S1 (continued)

T. gondii nuclear factors	Protein name	Protein accession number	Protein molecular weight (Da)	Number of unique peptides	Percentage sequence coverage	Peptide sequence	Nuclear localization using YFP tagging and/or specific antibodies	Slice number
	Centromere/microtubule binding protein, putative TGME49_014210 [Toxoplasma gondii ME49]	gi|211968418	56 726	10	28.9%	ALETLTGALFQRPPVVAAVK		**23-24**
						DHLVTMHDVLDAMHVYDSTR		
						DSCVNAICYGAK		
						EATSDPGLVASAGTEGGK		
						KTDNYAIEPNSSSPPIDTSK		
						LCGSEETENGEATEAAVKEEEQPR		
						LLDYDEQR		
						MKEATSDPGLVASAGTEGGK		
						RTIFPLELLLTGFPR		
						TPASWLK		
	Nucleolar phosphoprotein nucleolin, putative TGME49_091930 [Toxoplasma gondii ME49]	gi|211966199	73 100	3	6.1%	GIAFITFETEEAAQK		**28**
						GQAFIEFDSIESATK		
						SLFEECGEIQEVR		
	krr1 family, zinc finger-containing protein TGME49_019150 [Toxoplasma gondii ME49]	gi|211968343	99 371	2	4,0%	AFVDAAEGVEEGGAFLQK		**32**
						ALADDVAYAPLAEDEER		
	sec63 domain-containing DEAD/DEAH box helicase, putative TGME49_023390 [Toxoplasma gondii ME49]	gi|211963689	247 618	19	12.7%	ALVAEQVQAFSQR		**35**
						ASNRYPVVNVEFQVSK		
						DTSTGVAPAVHLGEPTGEPESLAGR		
						DVGDWLGVSSSGLFNFHPSVR		
						EILQSEAEAVK		
						GATVLDADVTEIFFYKPTTQQTR		
						GLFFFGNHYRPVPLK		
						GTQVYLPEK		
						IVAMANSLANAK		
						LELAAFVQPLTR		
						LFSLSSEFK		
						LTAIDLLLQAAADDDPKK		
						LVEQSQLILATPENWDFVSR		
						LVGLSATLPNYDDVAVCLR		
						LVIIDEIHLLHDAR		
						MEQVYSGSFK		
						WLLSESSLPISFK		
						YENFELAK		
						YNTMNEVTYEK		

S1 (continued)

T. gondii nuclear factors	Protein name	Protein accession number	Protein molecular weight (Da)	Number of unique peptides	Percentage sequence coverage	Peptide sequence	Nuclear localization using YFP tagging and/or specific antibodies	Slice number
**Enzymatic and chaperone functions**								
	Receptor for activated C kinase, RACK protein, putative TGME49_016880 [Toxoplasma gondii ME49]	gi|211968660	35 128	10	43.9%	DGVAMLWDVNEGK		**12-13**
						FSPSANKPLIVSCGWDK		
						GVLEGHTDCVTAISTPSLK		
						IWDLENK		
						LWDLNAGVTVR		
						LWNTLAECK		
						NVLSEITPEK		
						SFQGHTSDVNSVAFSPDNR		
						SGAPWCTSLNWSHDGR		
						VIVWTLNDNPDDSGSVGYAR		
	Acid phosphatase, putative TGME49_019320 [Toxoplasma gondii ME49]	gi|211968360	46 589	3	11.6%	LAPADATEAAAAENHGYPK		**18**
						LVSGTTGETLYTHK		
						WQSEFENVYSDANGALK		
	NADP-specific glutamate dehydrogenase, putative TGME49_093180 [Toxoplasma gondii ME49]	gi|211967784	53 642	5	13.1%	AANAGGVAVSGLEMSQNAMR		**18**
						DVPAGDIGVGAR		
						GFPLGQIQR		
						IVAEGANMPTTR		
						VQYSSAAGPYK		
	delta-aminolevulinic acid dehydratase, putative TGME49_053900 [Toxoplasma gondii ME49]	gi|211967109	71 357	3	6.2%	AGADAVATYYAK		**23**
						AGADMVCPSDMMDGR		
						GYISEKDTVLEVLK		

S1 (continued)

T. gondii nuclear factors	Protein name	Protein accession number	Protein molecular weight (Da)	Number of unique peptides	Percentage sequence coverage	Peptide sequence	Nuclear localization using YFP tagging and/or specific antibodies	Slice number
	Heat shock protein 70, putative TGME49_073760 [Toxoplasma gondii ME49]	gi|211963641	72 276	19	35.7%	AIQEALDWLDK		**25-26-30**
						ATAGDTHLGGEDFDNR		
						DAGTIAGLSVLR		
						EIAEAYLGK		
						EVESVCTPIITK		
						FDDPSVQSDMK		
						FEELCMDYFR		
						IQQLITDFFNGK		
						KFDDPSVQSDMK		
						LVDFCVQDFK		
						NDAVEIIANDQGNR		
						NPENTIFDAK		
						NQLAEKEEFEAK		
						SINPDEAVAYGAAVQAAILK		
						SNQITITNDK		
						SQTFTTYADNQPGVLIQVYEGER		
						SVSEVVLVGGSTR		
						TTPSYVAFTDTER		
						VIAGPGDKPLIEVTYQGEK		
	Heat shock protein 70, putative TGME49_111720 [Toxoplasma gondii ME49]	gi|211962068	73 236	18	33.4%	DFFNGKEPNR		**26**
						DKDLLPYEIINK		
						DVEAVCNPIISK		
						ETAEQFLGK		
						FEELNSDLFQK		
						IINEPTAAAIAYGLDK		
						ITPSYVAFTDDDR		
						ITPSYVAFTDDDRK		
						LTPEEIER		
						MKETAEQFLGK		
						NALEGYLHSMK		
						NAVVTVPAYFNDAQR		
						NEATINPTNTLFDVK		
						NGILSVSAVDK		
						VDIIPNDQGNR		
						VEVENLMEGVDFSETLTR		
						VTEAQEWLNTNPDADAEETR		
						VYGQSGGPGAGGAAGGADDDDYGGHDEL		

S1 (continued)

T. gondii nuclear factors	Protein name	Protein accession number	Protein molecular weight (Da)	Number of unique peptides	Percentage sequence coverage	Peptide sequence	Nuclear localization using YFP tagging and/or specific antibodies	Slice number
	p97 protein TGME49_075650 [Toxoplasma gondii ME49]	gi|211969256	101 140	12	21.8%	AHPGPLAATLADPEGWALYR		**28**
						FAEWLEQQQQNPVEK		
						GNTTVPATYAEWMK		
						GPLHEVLQDPEGWK		
						GPLTTVLADPDGWDLYK		
						GQPVPDTYEEWR		
						IEATYEEWMAK		
						KAHEGPLEQVLADPEGWK		
						MVSEAPGVPSSPSTEVR		
						NMVESSGASIEQTFEEWAQSK		
						NVVDVPQSFEDFLQQPDQQK		
						YEDWVSHPTVR		
**Unknown functions**								
**TgNF4**	Hypothetical protein TGME49_005580 [Toxoplasma gondii ME49]	gi|211965453	51 203	11	26.6%	AFSAVIPESAK	**YES**	**23-24**
						EKPVPVYLTDLQTGAIR		
						ETEEPEFDSAAMR		
						GVPMVIYER		
						KPEENANTEEKQEDGTGGATSPAVSNLK		
						LEIPGFGLVSGGVDLVIAK		
						LHLFTMGR		
						QEDGTGGATSPAVSNLK		
						TRPPFAVDGR		
						TVLVHLLR		
						YGWCTAESR		
**TgNF5**	Hypothetical protein, conserved TGME49_021380 [Toxoplasma gondii ME49] ALBA 1	gi|211967631	27 211	7	25.1%	AVTLAEVLK	**YES**	**13**
						AVTLAEVLKR		
						DKEPIGSNEIR		
						DPGYQAPIDESLVK		
						EMKPEEITK		
						LLTEQNMR		
						VMNYVAYAAR		
**TgNF6**	Hypothetical protein, conserved TGME49_018820 [Toxoplasma gondii ME49] ALBA 2	gi|211968320	15 374	5	33.8%	DDKPACDEVIITGLGMATK	**YES**	**2-4-5**
						TAIGAASLLQR		
						TPEFAEQIK		
						TPEFAEQIKK		
						VETSYFSSTR		

S1 (continued)

T. gondii nuclear factors	Protein name	Protein accession number	Protein molecular weight (Da)	Number of unique peptides	Percentage sequence coverage	Peptide sequence	Nuclear localization using YFP tagging and/or specific antibodies	Slice number
**TgNF7**	Hypothetical protein TGME49_048810 [Toxoplasma gondii ME49]	gi|211964891	54 264	9	22.5%	DMSATEYELVVLK	**?**	**21**
						EGHMVVGDESAVITLK		
						FVGPAAEEVER		
						IISDIPEAEK		
						MFQDWLSVYATADR		
						SPHVAVGLSGGER		
						TDMCLFVK		
						VDLPSSISQADWER		
						YRNPLAESSEEAK		
	Hypothetical protein TGME49_013050 [Toxoplasma gondii ME49]	gi|211968718	19 684	2	12.4%	EGVQSAAANITEK		**2**
						VANLQQAMR		
	Hypothetical protein, conserved TGME49_018820 [Toxoplasma gondii ME49]	gi|211968320	15 374	2	33.8%	TPEFAEQIK		**4**
						VETSYFSSTR		
	Hypothetical protein TGME49_028630 [Toxoplasma gondii ME49]	gi|211964139	24 254	2	12.6%	GTLQATGASSPISSQK		**4**
						SSWGWSTPPDQWK		
	Hypothetical protein TGME49_007440 [Toxoplasma gondii ME49]	gi|211967222	34 765	9	28.9%	EVMMIVMQR		**10**
						FEAGNMAMVTGGHNVGR		
						GVPALITHDGR		
						IAAPHHWMLDK		
						ISNVFVIGK		
						ISNVFVIGKGEK		
						LRYALTYR		
						TDQCYPAGFMDVISIEK		
						VRTDQCYPAGFMDVISIEK		
	Hypothetical protein, conserved TGME49_119850 [Toxoplasma gondii ME49]	gi|211967516	56 644	2	7.3%	GDYYGGYGPGSSSGGPQSDR		**15**
						TGAASTQAGSDAATMNSTR		
	Hypothetical protein [Toxoplasma gondii RH]	gi|95007135	83 567	4	8.0%	EVASMEGASPAAFQAAQQR		**16**
						GAFLGAGATGIVTELIEK		
						KLVDEHPFFQVVEDA		
						LLLDPTHETR		
	Hypothetical protein TGME49_107810 [Toxoplasma gondii ME49]	gi|211969572	44 152	2	7.8%	EAMTVEELQAAVHEAR		**19**
						GQDANAPESAAETAPPR		
	Hypothetical protein TGME49_032440 [Toxoplasma gondii ME49]	gi|211965778	54 027	3	7.1%	LGFSSTEEIDKR		**20**
						SRLDELQAQMDK		
						YQAFLAQQR		

S1 (continued)

T. gondii nuclear factors	Protein name	Protein accession number	Protein molecular weight (Da)	Number of unique peptides	Percentage sequence coverage	Peptide sequence	Nuclear localization using YFP tagging and/or specific antibodies	Slice number
	Hypothetical protein TGME49_040060 [Toxoplasma gondii ME49]	gi|211964299	88 515	4	8.3%	AVPSGEQPPPQPPPPHSPPR		**21**
						GASTASLHSEPGAASMPGPSPK		
						GFDRPGTTGTTGPQVR		
						KSPPLPLQEQGR		
	Hypothetical protein TGME49_112490 [Toxoplasma gondii ME49]	gi|211962135	51 601	2	7.5%	SDLLSLLATDEDFYAEDR		**22**
						SSYVSEMILGEPDSTVR		
	Hypothetical protein TGME49_055350 [Toxoplasma gondii ME49]	gi|211962527	66 010	4	8.0%	HMFETEIR		**26**
						SGIDQAAEGGGWHGSK		
						TSMIVTTDAVEAR		
						VQSDPFAPPSR		
	Hypothetical protein TGME49_051170 [Toxoplasma gondii ME49]	gi|211965042	75 621	6	14.7%	ALLSSASGQVPEPLR		**27-28-29**
						EHPPPATGGPLPQR		
						ELYVELGVAER		
						IAEYMLEEHNPNPSSSEIR		
						QQGQDAGEGTAATGPSAPSVSSASPMSR		
						YTPVPTTTPPYPADVER		
	Hypothetical protein TGME49_030930 [Toxoplasma gondii ME49]	gi|211965658	34 572	2	7.0%	LQQNALLSGR		**28**
						VQQAVAEIVEEK		
	Conserved hypothetical protein TGGT1_102650 [Toxoplasma gondii GT1]	gi|221485011	2 905	2	13.6%	LESMASEIAAK		**29**
						LLAAGVDLSHVPGDASLTPGASDQGPR		
	Hypothetical protein TGME49_089380 [Toxoplasma gondii ME49]	gi|211966037	100 228	5	6.6%	ATVDYLDELGK		**29**
						AVAELAATNAGTEK		
						GALPASAPVR		
						QAVAATETTPFSSAQK		
						VMFVGDSAER		
	Hypothetical protein, conserved TGME49_106400 [Toxoplasma gondii ME49]	gi|211968066	142 579	5	6.0%	AAEQDAVGPALQASR		**31-33**
						GPSSGGGAAPVVLR		
						IVILAETGSTAEDVTNR		
						LQGEEEEEGEEEGEEGEGQR		
						VTLPAEGEGPR		
	Hypothetical protein, conserved TGME49_047450 [Toxoplasma gondii ME49]	gi|211964776	245 852	3	1.9%	AVVYLQSQQQAESAQYR		**33**
						GPLSPPYGSQR		
						IEEGEEGGFSHFGYR		
