# Supplementary material for: A Novel Toxoplasma gondii Nuclear Factor TgNF3 Is a Dynamic Chromatin-Associated Component, Modulator of Nucleolar Architecture and Parasite Virulence
Source: PLoS Pathog. 2011 Mar 31;7(3):e1001328. doi: 10.1371/journal.ppat.1001328 (PMC3068996; doi:10.1371/journal.ppat.1001328)
Supplement: Table S2 — Identification of T. gondii genes and promoters defined by genome-wide TgNF3 occupancy and ChIP-seq. (0.10 MB PDF) [file ppat.1001328.s004.pdf]

	Genes				Contigs		Gene names	
	Start	End	Chr	ID	Contig position	Reads	Sense	Description
1	137459	143397	ChrIa	TGME49_093180	144086 - 144346<br>144833 - 145078	11	-	NADP-specific glutamate dehydrogenase, putative
2	147102	155058	ChrIa	TGME49_093190	144086 - 144346<br>144833 - 145078	11	+	endonuclease/exonuclease/phosphatase domain-containing protein
3	163972	164370	ChrIa	TGME49_093210	159957 - 160310	2	+	hypothetical protein, conserved
4	365539	383355	ChrIa	TGME49_093500	361855 - 362182	1	+	hypothetical protein
5	384776	386720	ChrIa	TGME49_093510	387911 - 388101	1	-	polyADP-ribose polymerase domain-containing protein
6	421458	428938	ChrIa	TGME49_093570	429499 - 429683<br>429743 - 429977	3	-	translocation protein Sec62, putative
7	587848	591645	ChrIa	TGME49_093830	592306 - 592537	2	-	calpain, putative
8	675042	675113	ChrIa	TGME49_094070	675241 - 675544	1	-	tRNA-Gly
9	676757	683589	ChrIa	TGME49_094180	683827 - 684010	1	-	tRNA guanine-N7--methyltransferase, putative
10	686345	694895	ChrIa	TGME49_094190	683827 - 684010	1	+	hypothetical protein, conserved
11	707286	714876	ChrIa	TGME49_094210	715446 - 715655<br>716147 - 716413	4	-	hypothetical protein
12	873265	875754	ChrIa	TGME49_094390	876265 - 876517	2	-	myosin light chain TgMLC4, putative
13	1049541	1050071	ChrIa	TGME49_094660	1050167 - 1050425	1	-	hypothetical protein
14	1214241	1216567	ChrIa	TGME49_094850	1217884 - 1218151	1	-	actin-like protein 7, putative
15	1220630	1229749	ChrIa	TGME49_094860	1217884 - 1218151	1	+	hypothetical protein
16	1300192	1301631	ChrIa	TGME49_094980	1300072 - 1300341	1	+	hypothetical protein
17	1301842	1302471	ChrIa	TGME49_094990	1301409 - 1301734	1	+	hypothetical protein
18	1454785	1456851	ChrIa	TGME49_095980	1454008 - 1454335<br>1454359 - 1454742	2	+	hypothetical protein
19	1501115	1506666	ChrIa	TGME49_095790	1507579 - 1507851	1	-	hypothetical protein
20	1509497	1511206	ChrIa	TGME49_095780	1507579 - 1507851	1	+	hypothetical protein
21	1511727	1518040	ChrIa	TGME49_095770	1511543 - 1511802	1	+	hypothetical protein
22	1555635	1559045	ChrIa	TGME49_095730	1559398 - 1559565<br>1560496 - 1560752<br>1561121 - 1561354	3	-	TPR domain-containing protein

23	1562558	1567762	ChrIa	TGME49_095720	1559398 - 1559565<br>1560496 - 1560752<br>1561121 - 1561354	3	+	sulfite oxidase, putative
24	61456	63727	ChrIb	TGME49_007440	66170 - 66488	1	-	hypothetical protein
25	68762	74619	ChrIb	TGME49_007450	66170 - 66488	1	+	hypothetical protein, conserved
26	140424	143521	ChrIb	TGME49_007590	139579 - 139859	1	+	T-cell activation protein phosphatase 2C,
27	299314	299880	ChrIb	TGME49_007790	302107 - 302459<br>302628 - 302915	2	-	hypothetical protein
28	325524	327575	ChrIb	TGME49_007840	333835 - 334058	1	-	40S ribosomal protein S17, putative
29	335249	336361	ChrIb	TGME49_007850	333835 - 334058	1	+	hypothetical protein
30	575282	586308	ChrIb	TGME49_008070	586218 - 586439	1	-	inositol polyphosphate kinase domain-containing protein
31	701805	708311	ChrIb	TGME49_008430	708211 - 708344	1	-	serine proteinase inhibitor TgPI-2, putative
32	708502	709733	ChrIb	TGME49_008440	708211 - 708344	1	+	hypothetical protein, conserved
33	773707	774985	ChrIb	TGME49_008550	770782 - 771003<br>771497 - 771699<br>772095 - 772258	3	+	hypothetical protein
34	776606	782003	ChrIb	TGME49_008560	783388 - 783651	1	-	mitochondrial carrier domain-containing protein
35	784297	790712	ChrIb	TGME49_008570	783388 - 783651	1	+	hypothetical protein
36	822287	825582	ChrIb	TGME49_008720	827661 - 828046<br>830992 - 831094	3	-	phosphatase, putative
37	855707	857596	ChrIb	TGME49_008780	854734 - 855005	1	+	ubiquitin-conjugating enzyme E2, putative
38	985374	993530	ChrIb	TGME49_009020	995168 - 995473	1	-	hypothetical protein
39	996576	998954	ChrIb	TGME49_009030	995168 - 995473	1	+	actin
40	1090710	1091995	ChrIb	TGME49_009170	1091874 - 1092120<br>1093429 - 1093789	3	-	hypothetical protein
41	1098137	1099767	ChrIb	TGME49_009180	1091874 - 1092120<br>1093429 - 1093789	3	+	PAN domain-containing protein, putative
42	1151808	1156638	ChrIb	TGME49_009260	1157398 - 1157686<br>1158207 - 1158450	3	-	cytochrome c oxidase, putative
43	1247950	1251784	ChrIb	TGME49_009470	1252320 - 1252798	3	-	hypothetical protein
44	1253119	1254432	ChrIb	TGME49_009480	1252320 - 1252798	3	+	hypothetical protein
45	1364497	1367400	ChrIb	TGME49_009630	1368095 - 1368379	1	-	hypothetical protein
46	1368784	1370252	ChrIb	TGME49_009640	1368095 - 1368379	1	+	hypothetical protein
47	1403629	1405121	ChrIb	TGME49_009700	1403271 - 1403599	2	+	hypothetical protein

48	1484602	1488979	ChrIb	TGME49_009850	1491254 - 1491599	5	-	hypothetical protein
49	1492391	1496468	ChrIb	TGME49_009860	1491254 - 1491599	5	+	nucleolar phosphoprotein p130, putative
50	1554454	1561232	ChrIb	TGME49_009940	1553606 - 1553873	1	+	hypothetical protein
51	1820247	1821911	ChrIb	TGME49_121420	1817774 - 1818008	1	+	kelch motif domain-containing protein
52	1825445	1836350	ChrIb	TGME49_121410	1836624 - 1836932	1	-	hypothetical protein
53	1837639	1838908	ChrIb	TGME49_121400	1836624 - 1836932	1	+	microsomal signal peptidase subunit SPCS1 domain-containing protein
54	3198	8889	ChrII	TGME49_020840	9573 - 9793	1	-	threonine synthase, putative
55	15972	18893	ChrII	TGME49_020870	21246 - 21613	1	-	hypothetical protein
56	21396	23674	ChrII	TGME49_020880	21246 - 21613	1	+	hypothetical protein
57	156781	162917	ChrII	TGME49_021250	154367 - 154664	1	+	hypothetical protein
58	519935	534178	ChrII	TGME49_021670	535396 - 535536<br>535561 - 535848	2	-	transcription elongation factor FACT 140 kDa, putative
59	1116481	1118211	ChrII	TGME49_022420	1115392 - 1115584	2	+	hypothetical protein
60	1239901	1252924	ChrII	TGME49_022860	1252628 - 1252938	2	-	eukaryotic translation initiation factor 3 subunit 9, putative
61	1253266	1255403	ChrII	TGME49_022870	1252628 - 1252938	2	+	hypothetical protein
62	1358164	1359921	ChrII	TGME49_023030	1360539 - 1360811	3	-	hypothetical protein
63	1586564	1587145	ChrII	TGME49_097190	1587564 - 1587793	1	-	hypothetical protein, conserved
64	1588241	1597193	ChrII	TGME49_097200	1587564 - 1587793	1	+	hypothetical protein
65	1889638	1893274	ChrII	TGME49_097670	1888874 - 1889179<br>1889209 - 1889542	2	+	hypothetical protein
66	290406	294793	ChrIII	TGME49_075670	294621 - 294887<br>296572 - 296812	3	-	hypothetical protein, conserved
67	321020	325321	ChrIII	TGME49_075620	327960 - 328369	2	-	hypothetical protein
68	596709	597381	ChrIII	TGME49_052490	598090 - 598325	1	-	vacuolar protein sorting 29, putative
69	979245	986216	ChrIII	TGME49_053490	978822 - 979186	2	+	hypothetical protein
70	1038518	1043589	ChrIII	TGME49_053580	1037221 - 1037433	1	+	CMGC kinase, CDK family
71	1207413	1214328	ChrIII	TGME49_053810	1215877 - 1216210	3	-	hypothetical protein
72	1217545	1219989	ChrIII	TGME49_053820	1215877 - 1216210	3	+	hypothetical protein, conserved
73	1434105	1435872	ChrIII	TGME49_054080	1432315 - 1432449	1	+	zinc transporter ZIP domain-containing
74	1564671	1566331	ChrIII	TGME49_054270	1564274 - 1564535	1	+	hypothetical protein
75	1581426	1592571	ChrIII	TGME49_054300	1579960 - 1580245	1	+	hypothetical protein
76	1735087	1738428	ChrIII	TGME49_054440	1740288 - 1740491	1	-	60S ribosomal protein L12, putative
77	1743620	1750167	ChrIII	TGME49_054450	1740288 - 1740491	1	+	cell division protein pelota, putative
78	1759977	1763520	ChrIII	TGME49_054480	1767764 - 1768091	1	-	WD-40 repeat-containing protein

79	1768385	1782821	ChrIII	TGME49_054490	1767764 - 1768091	1	+	hypothetical protein
80	644610	650675	ChrIV	TGME49_119910	650944 - 651169	1	-	hypothetical protein
81	965738	978376	ChrIV	TGME49_119370	978489 - 978707	1	-	hypothetical protein
82	982436	983848	ChrIV	TGME49_119360	978489 - 978707	1	+	SRS17
83	1117577	1120907	ChrIV	TGME49_118700	1115661 - 1115854	1	+	eukaryotic translation initiation factor 6, putative
84	1130628	1139119	ChrIV	TGME49_118680	1139207 - 1139407	1	-	3',5'--cyclic-nucleotide phosphodiesterase, putative
85	1240402	1254114	ChrIV	TGME49_118510	1256318 - 1256577	1	-	N-ethylmaleimide-sensitive factor
86	1288961	1296264	ChrIV	TGME49_118460	1296305 - 1296485<br>1297701 - 1297988	7	-	cation-transporting ATPase, putative
87	1297774	1298555	ChrIV	TGME49_118450	1296305 - 1296485<br>1297701 - 1297988	7	+	zinc finger C3HC4 RING finger protein, putative
88	1708048	1713145	ChrIV	TGME49_011420	1713676 - 1713970	1	-	hypothetical protein
89	1714340	1724125	ChrIV	TGME49_011410	1713676 - 1713970	1	+	hypothetical protein, conserved
90	1824774	1826229	ChrIV	TGME49_011300	1824384 - 1824634	1	+	hypothetical protein
91	1837858	1841297	ChrIV	TGME49_011280	1841672 - 1841960	1	-	hypothetical protein
92	1907805	1910416	ChrIV	TGME49_011100	1911885 - 1912059	1	-	hypothetical protein
93	1942135	1947424	ChrIV	TGME49_011020	1948193 - 1948385<br>1950098 - 1950358	2	-	RNA-binding protein, putative
94	1950468	1956305	ChrIV	TGME49_011010	1948193 - 1948385<br>1950098 - 1950358	2	+	hypothetical protein
95	2267527	2276360	ChrIV	TGME49_101220	2279389 - 2279746	1	-	hypothetical protein
96	2279491	2284864	ChrIV	TGME49_101230	2279389 - 2279746	1	+	DNA repair protein, putative
97	2476991	2478657	ChrIV	TGME49_101690	2473852 - 2474052<br>2474800 - 2475195	2	+	hypothetical protein
98	348909	350827	ChrIX	TGME49_079350	351015 - 351325<br>352148 - 352422<br>355077 - 355432	6	-	hypothetical protein
99	442807	447722	ChrIX	TGME49_067810	441090 - 441310	1	+	Rab 5
100	499451	502301	ChrIX	TGME49_067740	498694 - 498904	1	+	hypothetical protein
101	702773	704730	ChrIX	TGME49_067530	705665 - 705980	1	-	hypothetical protein
102	706316	707571	ChrIX	TGME49_067520	705665 - 705980	1	+	hypothetical protein
103	1104784	1105386	ChrIX	TGME49_066720	1105960 - 1106207<br>1106320 - 1106652	3	-	hypothetical protein, conserved

104	1106726	1110234	ChrIX	TGME49_066710	1105960 - 1106207<br>1106320 - 1106652	3	+	NEK kinase
105	1174840	1177532	ChrIX	TGME49_066620	1180172 - 1180385	1	-	thioredoxin, putative
106	1313236	1314477	ChrIX	TGME49_066330	1311016 - 1311428	4	+	hypothetical protein
107	1367867	1375705	ChrIX	TGME49_066140	1376149 - 1376412	4	-	sterol-regulatory element binding protein site 2 protease, putative
108	1564412	1576267	ChrIX	TGME49_065770	1579698 - 1579853	1	-	hypothetical protein
109	1579895	1581051	ChrIX	TGME49_065660	1579698 - 1579853	1	+	hypothetical protein
110	1704988	1705708	ChrIX	TGME49_065350	1704956 - 1705381	1	+	acyl-CoA thioester hydrolase, putative
111	1835316	1841872	ChrIX	TGME49_065150	1842117 - 1842323<br>1843423 - 1843622	4	-	hypothetical protein
112	1853426	1855521	ChrIX	TGME49_065120	1852592 - 1852866<br>1853057 - 1853257	2	+	hypothetical protein, conserved
113	2420233	2444533	ChrIX	TGME49_088050	2445030 - 2445272	2	-	selective LIM-binding factor, putative
114	2449033	2452272	ChrIX	TGME49_088060	2445030 - 2445272	2	+	hypothetical protein, conserved
115	3173801	3175973	ChrIX	TGME49_089210	3178447 - 3178812	2	-	prefoldin subunit, putative
116	3178861	3189830	ChrIX	TGME49_089220	3178447 - 3178812	2	+	hypothetical protein
117	3424591	3430389	ChrIX	TGME49_089580	3430932 - 3431258	1	-	strictosidine synthase domain-containing protein
118	3465754	3469081	ChrIX	TGME49_089680	3469577 - 3469788	1	-	Ras family domain-containing protein
119	3759242	3760324	ChrIX	TGME49_090250	3760918 - 3761172	1	-	hypothetical protein, conserved
120	3761022	3764123	ChrIX	TGME49_090260	3760918 - 3761172	1	+	hypothetical protein, conserved
121	4323074	4323742	ChrIX	TGME49_091300	4326039 - 4326281	2	-	RER1 protein, putative
122	4476664	4477092	ChrIX	TGME49_091860	4477049 - 4477347	2	-	hypothetical protein
123	4477493	4481656	ChrIX	TGME49_091870	4477049 - 4477347	2	+	hypothetical protein
124	4647791	4652267	ChrIX	TGME49_092110	4647363 - 4647628	1	+	formate/nitrite transporter, putative
125	4788369	4793296	ChrIX	TGME49_092340	4784355 - 4784606<br>4787656 - 4788231	3	+	zinc finger C3HC4 RING finger protein, putative
126	4814054	4821550	ChrIX	TGME49_092400	4811820 - 4812142	1	+	hypothetical protein
127	4863179	4868687	ChrIX	TGME49_010470	4869985 - 4870226	1	-	hypothetical protein
128	5278853	5293914	ChrIX	TGME49_105040	5294259 - 5294397	1	-	hypothetical protein

129	5296237	5297812	ChrIX	TGME49_105050	5294259 - 5294397	1	+	calmodulin, putative
130	5357374	5365489	ChrIX	TGME49_105180	5366386 - 5366673	1	-	sodium/hydrogen exchanger, putative
131	5689129	5691675	ChrIX	TGME49_105800	5691847 - 5692048	1	-	hypothetical protein, conserved
132	5693087	5695576	ChrIX	TGME49_105810	5691847 - 5692048	1	+	hypothetical protein
133	5703003	5704922	ChrIX	TGME49_105830	5702064 - 5702329	2	+	EF hand family protein
134	5816290	5816979	ChrIX	TGME49_106010	5813734 - 5814032	3	+	hypothetical protein
135	5849997	5856900	ChrIX	TGME49_106030	5858363 - 5858664<br>5858789 - 5859040	2	-	hypothetical protein
136	5929372	5937091	ChrIX	TGME49_106220	5928557 - 5928794	1	+	formin binding protein, putative
137	6129484	6134961	ChrIX	TGME49_106360	6128945 - 6129369	3	+	hypothetical protein
138	6199948	6203431	ChrIX	TGME49_106470	6197979 - 6198392<br>6199646 - 6199923	4	+	isoprenylcysteine carboxylmethyltransferase, putative
139	31093	31808	ChrV	TGME49_096340	32589 - 32697	1	-	hypothetical protein
140	73339	79281	ChrV	TGME49_087500	79929 - 80125	1	-	TCP-1/cpn60 family chaperonin, putative
141	147507	154164	ChrV	TGME49_020110	159085 - 159323<br>159867 - 160085	5	-	hypothetical protein
142	161600	165051	ChrV	TGME49_020120	159085 - 159323<br>159867 - 160085	5	+	hypothetical protein
143	257924	260232	ChrV	TGME49_020250	261282 - 261467	1	-	chloride channel, nucleotide-sensitive, 1A, putative
144	261635	263621	ChrV	TGME49_020260	261282 - 261467	1	+	hypothetical protein, conserved
145	265224	268338	ChrV	TGME49_020270	269444 - 269662	1	-	hypothetical protein, conserved
146	271238	275200	ChrV	TGME49_020280	269444 - 269662	1	+	SCP-like domain-containing protein
147	533657	533729	ChrV	TGME49_012750	535626 - 535977	1	-	tRNA-Met
148	606151	609069	ChrV	TGME49_012860	604684 - 604954	2	+	hypothetical protein
149	896709	900949	ChrV	TGME49_013340	901027 - 901205	1	-	glucose-methanol-choline oxidoreductase domain-containing protein
150	1205210	1217429	ChrV	TGME49_013720	1219889 - 1220250	1	-	hypothetical protein
151	1736410	1737967	ChrV	TGME49_086550	1735024 - 1735327<br>1735335 - 1735664	2	+	hypothetical protein, conserved
152	1867429	1873533	ChrV	TGME49_086210	1874410 - 1874696	2	-	serine/threonine protein phosphatase, putative
153	1918208	1922116	ChrV	TGME49_086130	1922340 - 1922704	1	-	hypothetical protein

154	2064055	2064126	ChrV	TGME49_085960	2063130 - 2063312	1	+	tRNA-Pro
155	2233388	2236549	ChrV	TGME49_085690	2236505 - 2236722	2	-	notch DSL domain-containing protein
156	2238517	2241643	ChrV	TGME49_085680	2236505 - 2236722	2	+	dihydrolipoamide acyltransferase, putative
157	2243969	2245478	ChrV	TGME49_085670	2247717 - 2248018<br>2249266 - 2249573	5	-	hypothetical protein
158	2249917	2266294	ChrV	TGME49_085660	2247717 - 2248018<br>2249266 - 2249573	5	+	DEAD/DEAH box helicase domain-containing protein
159	2585632	2587209	ChrV	TGME49_084660	2583460 - 2583757<br>2584283 - 2584521	4	+	hypothetical protein, conserved
160	2680813	2683091	ChrV	TGME49_084540	2684157 - 2684440	2	-	ATP synthase subunit O, putative
161	2684896	2689058	ChrV	TGME49_084530	2684157 - 2684440	2	+	hypothetical protein
162	2916593	2918813	ChrV	TGME49_083790	2921175 - 2921358	1	-	protein kinase
163	43243	44019	ChrVI	TGME49_038060	41688 - 41868	1	+	EF-hand domain-containing protein
164	237949	238503	ChrVI	TGME49_038460	238528 - 238723	1	-	SRS22B
165	266959	267507	ChrVI	TGME49_038630	267407 - 267707	3	-	SRS22H
166	268023	268490	ChrVI	TGME49_038640	267407 - 267707	3	+	hypothetical protein
167	520543	526732	ChrVI	TGME49_039290	527497 - 527707	1	-	hypothetical protein
168	531956	538662	ChrVI	TGME49_039300	527497 - 527707	1	+	hypothetical protein
169	746233	750035	ChrVI	TGME49_039540	750539 - 750846	1	-	LEM3 / CDC50 family protein
170	755180	756882	ChrVI	TGME49_039550	750539 - 750846	1	+	roadblock/LC7 domain-containing protein
171	1009780	1012273	ChrVI	TGME49_039920	1009155 - 1009456	1	+	hypothetical protein, conserved
172	1350347	1353751	ChrVI	TGME49_040640	1346114 - 1346418	1	+	Casein kinase one CK1 TgCK1a
173	1633296	1634372	ChrVI	TGME49_041130	1631224 - 1631428	1	+	hypothetical protein
174	1885439	1886940	ChrVI	TGME49_042120	1884413 - 1885023	6	+	hypothetical protein
175	1952175	1954344	ChrVI	TGME49_042350	1954577 - 1954818	2	-	TPR Domain containing protein
176	2051944	2054889	ChrVI	TGME49_042580	2054879 - 2055104	1	-	iron only hydrogenase large subunit, C-terminal domain-containing protein
177	2055330	2056983	ChrVI	TGME49_042590	2054879 - 2055104	1	+	hypothetical protein
178	2069805	2072364	ChrVI	TGME49_042600	2074425 - 2074639	1	-	6-phosphogluconate dehydrogenase, putative
179	2456839	2458481	ChrVI	TGME49_043260	2458527 - 2458857	1	-	hypothetical protein

180	2564287	2570176	ChrVI	TGME49_043380	2562643 - 2562925	1	+	hypothetical protein
181	2782604	2786727	ChrVI	TGME49_043720	2786502 - 2786760	2	-	peroxisomal biogenesis factor 11 domain-containing protein
182	2788570	2793124	ChrVI	TGME49_043730	2786502 - 2786760	2	+	p36 protein
183	2821310	2821789	ChrVI	TGME49_043770	2822985 - 2823255	1	-	hypothetical protein
184	2824146	2826080	ChrVI	TGME49_043780	2822985 - 2823255	1	+	hypothetical protein
185	2863906	2870659	ChrVI	TGME49_043930	2871989 - 2872140	2	-	hypothetical protein
186	2939199	2941289	ChrVI	TGME49_044030	2936567 - 2936917<br>2937800 - 2938075<br>2938879 - 2939119	5	+	hypothetical protein
187	3192703	3198834	ChrVI	TGME49_044380	3199311 - 3199644<br>3200413 - 3200782	2	-	hypothetical protein, conserved
188	3200507	3206948	ChrVI	TGME49_044390	3209694 - 3209978	1	-	coatomer epsilon subunit, putative
189	3210158	3211987	ChrVI	TGME49_044400	3209694 - 3209978	1	+	hypothetical protein
190	3249193	3254642	ChrVI	TGME49_044460	3258007 - 3258270<br>3259168 - 3259378	6	-	hypothetical protein
191	456639	459632	ChrVIIa	TGME49_080380	455672 - 455937	1	+	non-transmembrane antigen
192	481882	485310	ChrVIIa	TGME49_104470	486418 - 486653	1	-	oxidoreductase, putative
193	487731	489669	ChrVIIa	TGME49_104480	486418 - 486653	1	+	3-oxo-5 alpha-steroid delta 4-dehydrogenase alpha 2, putative
194	583917	587600	ChrVIIa	TGME49_104670	587539 - 587638	1	-	internalin, putative
195	697825	707630	ChrVIIa	TGME49_006670	710329 - 710542	3	-	hypothetical protein
196	712777	713943	ChrVIIa	TGME49_006660	710329 - 710542	3	+	hypothetical protein
197	737932	739635	ChrVIIa	TGME49_006630	742589 - 742856	1	-	hypothetical protein
198	745471	747573	ChrVIIa	TGME49_006620	742589 - 742856	1	+	hypothetical protein
199	979933	981574	ChrVIIa	TGME49_006410	976536 - 976654	1	+	hypothetical protein
200	1164398	1167777	ChrVIIa	TGME49_005720	1168390 - 1168588	1	-	adenosine/AMP deaminase domain containing protein
201	1168932	1173906	ChrVIIa	TGME49_005710	1168390 - 1168588	1	+	lysyl-tRNA synthetase, putative
202	1220769	1238439	ChrVIIa	TGME49_005620	1239155 - 1239458	1	-	hypothetical protein

203	1240675	1241130	ChrVIIa	TGME49_005610	1239155 - 1239458	1	+	hypothetical protein, conserved
204	1444700	1444779	ChrVIIa	TGME49_005310	1443586 - 1443820	1	+	tRNA-Leu
205	1543140	1550619	ChrVIIa	TGME49_005150	1541025 - 1541304	1	+	hypothetical protein
206	1561036	1587512	ChrVIIa	TGME49_005130	1558202 - 1558315 1558711 - 1559036	2	+	glyoxalase, putative
207	1649021	1650207	ChrVIIa	TGME49_004890	1648823 - 1649109	1	+	hypothetical protein
208	1659087	1660811	ChrVIIa	TGME49_004870	1661564 - 1661770	1	-	hypothetical protein
209	1663586	1686354	ChrVIIa	TGME49_004560	1661564 - 1661770	1	+	type I fatty acid synthase, putative
210	1769067	1776641	ChrVIIa	TGME49_004410	1776877 - 1777073	1	-	endonuclease/exonuclease/phosphatase domain-containing protein
211	1780178	1787919	ChrVIIa	TGME49_004400	1776877 - 1777073	1	+	ATP synthase alpha chain, putative
212	1815600	1822592	ChrVIIa	TGME49_004360	1814784 - 1815049	1	+	subtilase family serine protease, putative
213	2017894	2018652	ChrVIIa	TGME49_004000	2018871 - 2019182 2020042 - 2020397	2	-	hypothetical protein
214	2097789	2104823	ChrVIIa	TGME49_003910	2106240 - 2106545	1	-	TBC domain-containing protein
215	2135043	2139503	ChrVIIa	TGME49_003870	2139692 - 2140012	1	-	isy1-like splicing family domain-containing protein
216	2347324	2350929	ChrVIIa	TGME49_003640	2355324 - 2355514 2356062 - 2356302	2	-	hypothetical protein
217	2375607	2376896	ChrVIIa	TGME49_003590	2377776 - 2377964	1	-	hypothetical protein
218	2380855	2383638	ChrVIIa	TGME49_003580	2377776 - 2377964	1	+	NAD-binding domain-containing protein
219	2395888	2397120	ChrVIIa	TGME49_003560	2397184 - 2397521	1	-	hypothetical protein
220	2769464	2772044	ChrVIIa	TGME49_003110	2772467 - 2772748 2773792 - 2774003	3	-	citrate synthase, putative
221	2773899	2774446	ChrVIIa	TGME49_003100	2772467 - 2772748 2773792 - 2774003	3	+	hypothetical protein
222	2785341	2787695	ChrVIIa	TGME49_003060	2788249 - 2788510 2790344 - 2790611	3	-	hypothetical protein, conserved
223	2790918	2798241	ChrVIIa	TGME49_003050	2788249 - 2788510 2790344 - 2790611	3	+	hypothtetical protein, conserved
224	2945749	2954184	ChrVIIa	TGME49_002850	2942797 - 2943071	1	+	hypothetical protein

225	3452253	3458454	ChrVIIa	TGME49_002240	3460367 - 3460737	1	-	hypothetical protein
226	3462455	3471812	ChrVIIa	TGME49_002230	3460367 - 3460737	1	+	histone deactylase, putative
227	3923179	3932058	ChrVIIa	TGME49_001220	3934453 - 3934715	1	-	hypothetical protein
228	4326121	4328823	ChrVIIa	TGME49_082050	4328721 - 4329032	6	-	hypothetical protein
229	4383515	4385589	ChrVIIa	TGME49_082160	4383170 - 4383458	1	+	hypothetical protein
230	78151	81029	ChrVIIb	TGME49_064200	83970 - 84179	1	-	hypothetical protein
231	84414	90748	ChrVIIb	TGME49_064190	83970 - 84179	1	+	hypothetical protein
232	128367	129795	ChrVIIb	TGME49_064150	129943 - 130086	1	-	hypothetical protein
233	268239	275853	ChrVIIb	TGME49_063870	276153 - 276326	1	-	glutamyl-tRNA synthetase, putative
234	276801	280519	ChrVIIb	TGME49_063860	276153 - 276326	1	+	hypothetical protein
235	574071	574974	ChrVIIb	TGME49_063400	577720 - 577935	1	-	hypothetical protein
236	577912	582741	ChrVIIb	TGME49_063390	577720 - 577935	1	+	hypothetical protein
237	856582	867347	ChrVIIb	TGME49_063000	855468 - 855751	1	+	hypothetical protein, conserved
238	868948	873827	ChrVIIb	TGME49_062990	876380 - 876725	1	-	hypothetical protein
239	877377	880550	ChrVIIb	TGME49_062980	876380 - 876725	1	+	hypothetical protein
240	1590113	1597110	ChrVIIb	TGME49_061660	1596865 - 1597286<br>1600627 - 1600967	2	-	hypothetical protein
241	1965294	1970821	ChrVIIb	TGME49_061010	1971900 - 1972172	1	-	26S protease regulatory subunit 8, putative
242	1972204	1980585	ChrVIIb	TGME49_061000	1971900 - 1972172	1	+	DNA mismatch repair protein, putative
243	2121627	2131574	ChrVIIb	TGME49_060600	2121426 - 2121838	1	+	pumilio-family RNA binding repeat domain-containing protein
244	2139081	2143978	ChrVIIb	TGME49_060580	2138912 - 2139146	2	+	hypothetical protein
245	2302848	2303943	ChrVIIb	TGME49_060400	2300897 - 2301127<br>2302724 - 2302925	2	+	hypothetical protein
246	2628349	2632303	ChrVIIb	TGME49_059880	2633151 - 2633375	1	-	hypothetical protein
247	2634917	2641526	ChrVIIb	TGME49_059870	2633151 - 2633375	1	+	hypothetical protein
248	2776228	2783809	ChrVIIb	TGME49_059530	2775771 - 2776031	1	+	UDP-N-acetyl-D-galactosamine:polypeptide N-acetylgalactosaminyltransferase T1, putative
249	2796363	2797667	ChrVIIb	TGME49_059300	2796234 - 2796410	1	+	SRS26B

250	2879560	2885879	ChrVIIb	TGME49_059190	2886649 - 2886899<br>2887093 - 2887313	7	-	hypothetical protein
251	3095433	3100813	ChrVIIb	TGME49_058870	3102829 - 3103005	2	-	hypothetical protein
252	3104472	3108784	ChrVIIb	TGME49_058860	3102829 - 3103005	2	+	hypothetical protein
253	3224947	3233900	ChrVIIb	TGME49_058700	3234335 - 3234589	1	-	hypothetical protein
254	3389529	3391443	ChrVIIb	TGME49_058460	3391309 - 3391687<br>3392162 - 3392385<br>3394263 - 3394477	4	-	hypothetical protein
255	3396069	3402026	ChrVIIb	TGME49_058450	3391309 - 3391687<br>3392162 - 3392385<br>3394263 - 3394477	4	+	hypothetical protein, conserved
256	3914473	3915462	ChrVIIb	TGME49_057570	3917092 - 3917310	1	-	hypothetical protein
257	3919401	3922962	ChrVIIb	TGME49_057560	3917092 - 3917310	1	+	WD domain, G-beta repeat-containing protein
258	3938035	3941620	ChrVIIb	TGME49_057530	3944102 - 3944388	1	-	hypothetical protein, conserved
259	3944715	3947336	ChrVIIb	TGME49_057520	3944102 - 3944388	1	+	synaptobrevin-like protein, putative
260	4034671	4040853	ChrVIIb	TGME49_057370	4031749 - 4032090<br>4032203 - 4032447<br>4033790 - 4034058	3	+	hypothetical protein
261	4077270	4077761	ChrVIIb	TGME49_057320	4077790 - 4078077	1	-	hypothetical protein
262	4080760	4082443	ChrVIIb	TGME49_057310	4077790 - 4078077	1	+	nuclear movement domain-containing protein
263	4153427	4160894	ChrVIIb	TGME49_057120	4163841 - 4164123	2	-	glucose transporter, putative
264	4614696	4617602	ChrVIIb	TGME49_055750	4617802 - 4618053	6	-	hypothetical protein
265	4768707	4771213	ChrVIIb	TGME49_055420	4772102 - 4772215<br>4772549 - 4772688	2	-	hypothetical protein
266	6252	8709	ChrVIII	TGME49_028990	9345 - 9678	1	-	zinc finger CCCH type protein, putative
267	44269	49910	ChrVIII	TGME49_029140	51658 - 51913<br>52354 - 52578	2	-	peroxisomal multifunctional enzyme, putative
268	53314	57291	ChrVIII	TGME49_029150	58910 - 59162<br>59610 - 59922	2	-	sugar permease, putative
269	59807	62205	ChrVIII	TGME49_029160	58910 - 59162<br>59610 - 59922	2	+	zinc finger DHHC domain-containing protein

270	164574	165518	ChrVIII	TGME49_029320	166222 - 166521	2	-	haloacid dehalogenase-like hydrolase domain-containing protein
271	166682	168259	ChrVIII	TGME49_029330	169149 - 169451<br>169506 - 169739	2	-	haloacid dehalogenase-like hydrolase domain containing protein
272	252746	258824	ChrVIII	TGME49_029440	258624 - 258990	2	-	zinc finger C3HC4 type RING finger protein, putative
273	369324	369869	ChrVIII	TGME49_029660	369895 - 370128	1	-	hypothetical protein
274	369947	373222	ChrVIII	TGME49_029670	369895 - 370128	1	+	ribosomal protein S23, putative
275	483347	490479	ChrVIII	TGME49_029950	481836 - 481997	1	+	26S protease regulatory subunit 6b, putative
276	547585	553795	ChrVIII	TGME49_030020	556048 - 556301	1	-	kinesin motor domain-containing protein
277	799153	805460	ChrVIII	TGME49_030460	805813 - 806183	1	-	dpy-30 motif-containing protein
278	807853	809229	ChrVIII	TGME49_030470	805813 - 806183	1	+	Rhoptry kinase family protein ROP46, putative
279	925048	927046	ChrVIII	TGME49_030640	927522 - 927658	1	-	hypothetical protein
280	928006	932862	ChrVIII	TGME49_030650	927522 - 927658	1	+	hypothetical protein
281	936868	941815	ChrVIII	TGME49_030660	942934 - 943177<br>947443 - 947666<br>950362 - 950631<br>950904 - 951333	7	-	hypothetical protein
282	953116	956220	ChrVIII	TGME49_030670	942934 - 943177<br>947443 - 947666<br>950362 - 950631<br>950904 - 951333	7	+	tubulin-tyrosine ligase family domain-containing protein
283	962262	965903	ChrVIII	TGME49_030680	966983 - 967378	4	-	hypothetical protein
284	1113367	1117268	ChrVIII	TGME49_030930	1111831 - 1112095	3	+	hypothetical protein
285	1231396	1232816	ChrVIII	TGME49_031090	1232941 - 1233130	2	-	hypothetical protein
286	1235672	1240297	ChrVIII	TGME49_031100	1232941 - 1233130	2	+	hypothetical protein
287	1326442	1334647	ChrVIII	TGME49_031210	1334670 - 1334890	2	-	EH protein, putative
288	1337354	1340086	ChrVIII	TGME49_031220	1334670 - 1334890	2	+	hypothetical protein
289	1375148	1384475	ChrVIII	TGME49_031370	1385713 - 1385988<br>1387610 - 1387965	3	-	patatin-like phospholipase domain-containing protein
290	1388087	1389872	ChrVIII	TGME49_031380	1385713 - 1385988<br>1387610 - 1387965	3	+	DNA-directed RNA polymerase II, putative

291	1621395	1626032	ChrVIII	TGME49_031900	1626554 - 1626892	1	-	acyl-CoA dehydrogenase, putative
292	1628325	1634005	ChrVIII	TGME49_031910	1626554 - 1626892	1	+	ATP synthase gama chain, putative
293	1650511	1655336	ChrVIII	TGME49_031930	1655642 - 1655856	1	-	hypothetical protein
294	1657931	1663056	ChrVIII	TGME49_031940	1655642 - 1655856	1	+	thiF family domain-containing protein
295	2031756	2045829	ChrVIII	TGME49_032360	2046265 - 2046479	1	-	RNA polymerase Rpb1 C-terminal repeat domain-containing protein / exonuclease domain-containing protein
296	2114029	2115942	ChrVIII	TGME49_032480	2113691 - 2114009	2	+	paramyosin, putative
297	2316541	2330422	ChrVIII	TGME49_033000	2331439 - 2331771<br>2332075 - 2332383	4	-	supt5h protein, putative
298	2335952	2341851	ChrVIII	TGME49_033010	2331439 - 2331771<br>2332075 - 2332383	4	+	CMGC kinase, MAPK family TgMAPK2
299	2555557	2556822	ChrVIII	TGME49_033270	2557702 - 2557904	1	-	MORN repeat-containing protein
300	2874367	2874440	ChrVIII	TGME49_033880	2874629 - 2874909	4	-	tRNA-Ile
301	3235003	3236460	ChrVIII	TGME49_073810	3234978 - 3235200	6	+	hypothetical protein
302	3294496	3298369	ChrVIII	TGME49_073740	3298158 - 3298479<br>3299293 - 3299625	3	-	acetyl-CoA acyltransferase B, putative
303	3299360	3301867	ChrVIII	TGME49_073730	3303195 - 3303422	5	-	hypothetical protein
304	3835977	3837656	ChrVIII	TGME49_072620	3837995 - 3838327	1	-	hypothetical protein
305	3968791	3975902	ChrVIII	TGME49_072430	3976744 - 3976936	1	-	membrane-attack complex / perforin domain-containing protein
306	4316738	4327323	ChrVIII	TGME49_071870	4327763 - 4327951	1	-	zinc carboxypeptidase, putative
307	4513724	4514770	ChrVIII	TGME49_071390	4513327 - 4513563	1	+	hypothetical protein, conserved
308	4548485	4556098	ChrVIII	TGME49_071350	4556279 - 4556488	1	-	folylpolyglutamate synthase, putative
309	4557758	4559210	ChrVIII	TGME49_071340	4556279 - 4556488	1	+	hypothetical protein, conserved
310	4725115	4730553	ChrVIII	TGME49_071090	4733501 - 4733703	1	-	hypothetical protein
311	4735900	4736454	ChrVIII	TGME49_071080	4733501 - 4733703	1	+	hypothetical protein
312	4825698	4826306	ChrVIII	TGME49_070970	4827197 - 4827578	2	-	hypothetical protein
313	4829004	4835615	ChrVIII	TGME49_070960	4827197 - 4827578	2	+	hypothetical protein
314	5065533	5078433	ChrVIII	TGME49_070690	5080449 - 5080706	1	-	hypothetical protein, conserved

315	5081039	5081791	ChrVIII	TGME49_070680	5080449 - 5080706	1	+	hypothetical protein
316	5310228	5319586	ChrVIII	TGME49_070230	5321164 - 5321451	1	-	hypothetical protein
317	5321889	5326207	ChrVIII	TGME49_070220	5321164 - 5321451	1	+	hypothetical protein
318	5586395	5586466	ChrVIII	TGME49_069810	5585962 - 5586150	1	+	tRNA-Trp
319	5593926	5593997	ChrVIII	TGME49_069790	5593238 - 5593490	1	+	tRNA-Pro
320	5878583	5883172	ChrVIII	TGME49_069340	5883032 - 5883321	1	-	hypothetical protein
321	5883542	5887885	ChrVIII	TGME49_069330	5883032 - 5883321	1	+	hypothetical protein, conserved
322	5945684	5947008	ChrVIII	TGME49_069270	5945059 - 5945291	1	+	leucine rich repeat protein, putative
323	5975785	5975857	ChrVIII	TGME49_069220	5975855 - 5976046	1	-	tRNA-Lys
324	5977793	5982928	ChrVIII	TGME49_069210	5975855 - 5976046	1	+	hypothetical protein
325	6027809	6029575	ChrVIII	TGME49_069170	6029777 - 6029967	1	-	hypothetical protein
326	6088312	6097485	ChrVIII	TGME49_069060	6086637 - 6086939	1	+	hypothetical protein
327	6245895	6248259	ChrVIII	TGME49_068850	6249150 - 6249365	1	-	enolase
328	6363482	6366786	ChrVIII	TGME49_068680	6366551 - 6366835	1	-	hypothetical protein
329	6367482	6367984	ChrVIII	TGME49_068670	6366551 - 6366835	1	+	hypothetical protein
330	6753636	6754857	ChrVIII	TGME49_000230	6755316 - 6755614	2	-	PAN domain-containing protein
331	6769176	6772022	ChrVIII	TGME49_000280	6773438 - 6773670	1	-	WD-repeat protein, putative
332	6774168	6778232	ChrVIII	TGME49_000290	6773438 - 6773670	1	+	rhomboid family domain-containing protein
333	6825330	6830302	ChrVIII	TGME49_000370	6832922 - 6833202	3	-	protein farnesyltranstransferase beta subunit, putative
334	218717	220698	ChrX	TGME49_028630	216387 - 216503	1	+	hypothetical protein
335	499336	501218	ChrX	TGME49_028140	497311 - 497664	1	+	hypothetical protein
336	506421	524588	ChrX	TGME49_028120	503892 - 504155	1	+	hypothetical protein
337	718399	722093	ChrX	TGME49_027850	723746 - 723994	1	-	RNA recognition motif-containing protein
338	725423	732139	ChrX	TGME49_027840	723746 - 723994	1	+	hypothetical protein
339	846996	852420	ChrX	TGME49_027580	854124 - 854359	1	-	hypothetical protein
340	987952	990477	ChrX	TGME49_027290	991624 - 991801	1	-	histone deacetylase, putative
341	1172628	1189179	ChrX	TGME49_026910	1190876 - 1191059<br>1193147 - 1193397	4	-	glycogen debranching enzyme, putative

342	1316022	1320668	ChrX	TGME49_026770	1320727 - 1320908	1	-	cGMP-inhibited 3',5'-cyclic phosphodiesterase, putative
343	1324689	1330010	ChrX	TGME49_026750	1332505 - 1332688	1	-	hypothetical protein
344	1334783	1342207	ChrX	TGME49_026740	1332505 - 1332688	1	+	zinc finger C3HC4 RING finger protein, putative
345	1344726	1352360	ChrX	TGME49_026730	1352801 - 1352920	1	-	aconitate hydratase, putative
346	1355973	1358203	ChrX	TGME49_026720	1352801 - 1352920	1	+	translin, putative
347	1504043	1514501	ChrX	TGME49_026510	1515571 - 1515862<br>1516493 - 1516669	3	-	sec23/Sec24 helical domain-containing protein
348	1518245	1520644	ChrX	TGME49_026500	1515571 - 1515862<br>1516493 - 1516669	3	+	hypothetical protein
349	1717830	1719597	ChrX	TGME49_026240	1720442 - 1720663	1	-	zinc finger protein, putative
350	1721028	1722578	ChrX	TGME49_026230	1720442 - 1720663	1	+	hypothetical protein
351	1807930	1812343	ChrX	TGME49_026020	1813801 - 1814094	1	-	transmembrane domain-containing protein
352	1813867	1815235	ChrX	TGME49_026010	1813801 - 1814094	1	+	pterin-4a-carbinolamine dehydratase
353	1976021	1979658	ChrX	TGME49_025800	1979493 - 1979758	2	-	ABC transporter, putative
354	1982363	1985719	ChrX	TGME49_025790	1979493 - 1979758	2	+	hypothetical protein
355	1994419	2003742	ChrX	TGME49_025770	2004515 - 2005058<br>2005294 - 2005527<br>2007358 - 2007639	5	-	Tyrosine kinase-like TKL protein
356	2010004	2014840	ChrX	TGME49_025760	2004515 - 2005058<br>2005294 - 2005527<br>2007358 - 2007639	5	+	hypothetical protein
357	2322892	2327135	ChrX	TGME49_025240	2321007 - 2321274<br>2321401 - 2321835	5	+	50s ribosomal protein L13, putative
358	2331215	2333622	ChrX	TGME49_025230	2336656 - 2337062	3	-	hypothetical protein
359	2438494	2442939	ChrX	TGME49_025110	2436166 - 2436419	1	+	hypothetical protein
360	2497906	2500692	ChrX	TGME49_025050	2501123 - 2501270<br>2505784 - 2506019	2	-	adenosylhomocysteinase, putative
361	2506109	2519517	ChrX	TGME49_025030	2501123 - 2501270<br>2505784 - 2506019	2	+	myosin heavy chain, putative
362	2771634	2772812	ChrX	TGME49_024790	2773754 - 2774032	2	-	SRS40A

363	2805625	2808600	ChrX	TGME49_024710	2808974 - 2809227	1	-	vacuolar sorting receptor protein, putative
364	3079622	3090729	ChrX	TGME49_024270	3094709 - 3095029	3	-	hypothetical protein, conserved
365	3157909	3161669	ChrX	TGME49_024220	3155095 - 3155398	1	+	serine/threonine protein phosphatase, putative
366	3227462	3239474	ChrX	TGME49_024160	3224593 - 3224812	2	+	hypothetical protein
367	3514842	3522160	ChrX	TGME49_023790	3513128 - 3513374<br>3513905 - 3514312	2	+	hypothetical protein, conserved
368	3780966	3788836	ChrX	TGME49_023440	3790871 - 3791095	1	-	ATP-dependent RNA helicase, putative
369	4082889	4096163	ChrX	TGME49_011890	4096049 - 4096373	2	-	hypothetical protein
370	4098004	4098579	ChrX	TGME49_011880	4096049 - 4096373	2	+	hypothetical protein, conserved
371	4225756	4227534	ChrX	TGME49_034290	4229848 - 4230212	1	-	hypothetical protein
372	4387640	4391737	ChrX	TGME49_034540	4393008 - 4393186	1	-	hypothetical protein
373	4436346	4443999	ChrX	TGME49_034610	4433144 - 4433422	1	+	hypothetical protein
374	4815719	4819750	ChrX	TGME49_035450	4820088 - 4820292	1	-	ubiquitin-conjugating enzyme, putative
375	4953954	4960408	ChrX	TGME49_035650	4951269 - 4951373<br>4953710 - 4954022	2	+	methionyl-tRNA formyltransferase, putative
376	4980923	4999089	ChrX	TGME49_035680	4998950 - 4999179	1	-	M16 family peptidase, putative
377	5001981	5003692	ChrX	TGME49_035690	4998950 - 4999179	1	+	hypothetical protein
378	5178361	5180909	ChrX	TGME49_036010	5182674 - 5182960	1	-	prenylcysteine oxidase, putative
379	5183317	5184837	ChrX	TGME49_036020	5182674 - 5182960	1	+	BT1 transmembrane domain-containing protein
380	5292169	5297782	ChrX	TGME49_036250	5291522 - 5291809	1	+	hypothetical protein, conserved
381	5324327	5326426	ChrX	TGME49_036400	5326695 - 5326864<br>5327831 - 5328087	3	-	hypothetical protein
382	5356496	5358751	ChrX	TGME49_036550	5359046 - 5359251	2	-	hypothetical protein
383	5360925	5369195	ChrX	TGME49_036560	5359046 - 5359251	2	+	hypothetical protein
384	5382596	5386249	ChrX	TGME49_036590	5386540 - 5386746	1	-	hypothetical protein, conserved
385	5393491	5399540	ChrX	TGME49_036620	5393122 - 5393319	1	+	protein kinase incomplete catalytic triad
386	5410182	5415050	ChrX	TGME49_036650	5416573 - 5416846	1	-	DEAD/DEAH box helicase, putative
387	5620314	5621942	ChrX	TGME49_037030	5623142 - 5623326	1	-	hypothetical protein

388	5634912	5636233	ChrX	TGME49_037070	5636831 - 5636972	4	-	hypothetical protein
389	5681793	5687876	ChrX	TGME49_037150	5688827 - 5689093	1	-	signal peptide peptidase domain-containing protein
390	6065500	6086829	ChrX	TGME49_014230	6062247 - 6062481<br>6062929 - 6063264	2	+	dopey, N-terminal domain-containing protein
391	6119023	6133686	ChrX	TGME49_014260	6118089 - 6118362	1	+	alpha-glucan water dikinase 1, putative
392	6191614	6193326	ChrX	TGME49_014330	6193455 - 6193720	3	-	hypothetical protein, conserved
393	6193519	6197132	ChrX	TGME49_014340	6193455 - 6193720	3	+	hypothetical protein, conserved
394	6495931	6498340	ChrX	TGME49_014870	6493616 - 6493915<br>6495689 - 6495967	8	+	ribosomal protein L9, N-terminal domain protein
395	6714322	6721370	ChrX	TGME49_015150	6724107 - 6724304	1	-	hypothetical protein
396	6729005	6731809	ChrX	TGME49_015160	6724107 - 6724304	1	+	hypothetical protein
397	6757011	6757529	ChrX	TGME49_015200	6756662 - 6756957	3	+	hypothetical protein
398	7011170	7013925	ChrX	TGME49_015660	7008224 - 7008443	3	+	hypothetical protein
399	7088838	7089816	ChrX	TGME49_015760	7088548 - 7089003	2	+	Cytidine and deoxycytidylate deaminase zinc-binding domain-containing protein
400	7350819	7351905	ChrX	TGME49_007130	7352703 - 7353039	2	-	SRS49A = SAG2Y
401	7358954	7360860	ChrX	TGME49_007160	7361824 - 7362400<br>7362592 - 7362949	4	-	SRS49D = SAG2C
402	7363083	7365954	ChrX	TGME49_007170	7361824 - 7362400<br>7362592 - 7362949	4	+	hypothetical protein
403	212334	216485	ChrXI	TGME49_106890	208978 - 209250	1	+	platelet binding protein GspB, putative
404	597690	599219	ChrXI	TGME49_109270	599610 - 599916	1	-	hypothetical protein
405	600529	602669	ChrXI	TGME49_109280	604215 - 604483	1	-	hypothetical protein
406	604479	607321	ChrXI	TGME49_109290	604215 - 604483	1	+	HD domain-containing protein
407	813248	817742	ChrXI	TGME49_109800	818095 - 818331	1	-	small nuclear ribonucleoprotein U1A, putative
408	900697	907436	ChrXI	TGME49_109930	908425 - 908686	1	-	alpha-N-acetylgalactosaminidase, putative
409	911252	919673	ChrXI	TGME49_109940	908425 - 908686	1	+	cardiolipin synthase, putative
410	935457	979511	ChrXI	TGME49_109980	979447 - 979555	1	-	dynein-1-alpha heavy chain, flagellar inner arm I1 complex, putative
411	981785	983521	ChrXI	TGME49_109990	979447 - 979555	1	+	hypothetical protein, conserved

412	1333515	1335087	ChrXI	TGME49_110440	1332346 - 1332647	1	+	phosphatidylinositol-4-phosphate 5-kinase, putative
413	1415530	1418956	ChrXI	TGME49_110540	1418956 - 1419137	1	-	hypothetical protein, conserved
414	1597637	1601435	ChrXI	TGME49_110810	1594474 - 1594773	2	+	apyrase, putative
415	1610625	1612370	ChrXI	TGME49_110830	1610492 - 1610602	1	+	delta3,5-delta2,4-dienoyl-CoA isomerase, putative
416	2381850	2384378	ChrXI	TGME49_112040	2380651 - 2380796	1	+	hypothetical protein
417	2715571	2720334	ChrXI	TGME49_112490	2715485 - 2715774	1	+	hypothetical protein
418	2735542	2740722	ChrXI	TGME49_112510	2740491 - 2740845<br>2741508 - 2741744<br>2742924 - 2743272<br>2743473 - 2743738	6	-	hypothetical protein
419	2809164	2811726	ChrXI	TGME49_112600	2807806 - 2808091	1	+	small heat shock protein 21
420	2977795	2979911	ChrXI	TGME49_112930	2981082 - 2981419	2	-	cystathionine beta-lyase, putative
421	4010701	4017120	ChrXI	TGME49_114340	4019519 - 4019625	1	-	sodium- and chloride-dependent neutral and basic amino acid transporter, putative
422	4047152	4050510	ChrXI	TGME49_114390	4051471 - 4051738	5	-	hypothetical protein
423	4052244	4057265	ChrXI	TGME49_114400	4051471 - 4051738	5	+	branched-chain alpha-keto acid dehydrogenase E1 component beta chain, putative
424	4140592	4150935	ChrXI	TGME49_114500	4154561 - 4155070	2	-	subtilisin-like protease TgSUB2
425	4155284	4155772	ChrXI	TGME49_114510	4154561 - 4155070	2	+	hypothetical protein, conserved
426	4716046	4719039	ChrXI	TGME49_115440	4714711 - 4714982	2	+	hypothetical protein
427	4724484	4728225	ChrXI	TGME49_115450	4719877 - 4720130<br>4720208 - 4720722	3	+	hypothetical protein, conserved
428	4941606	4943921	ChrXI	TGME49_115730	4940529 - 4940820<br>4941019 - 4941216	3	+	apical membrane antigen, putative
429	5083049	5086042	ChrXI	TGME49_115920	5087951 - 5088256	1	-	RNA polymerase II subunit RPB11, putative
430	5088176	5089688	ChrXI	TGME49_115930	5087951 - 5088256	1	+	hypothetical protein
431	5300086	5315164	ChrXI	TGME49_116440	5299862 - 5300117	2	+	phosphatidylinositol 3- and 4-kinase domain-containing protein
432	5419663	5419735	ChrXI	TGME49_116590	5418549 - 5418686	1	+	tRNA-Lys

433	5547619	5557919	ChrXI	TGME49_116730	5547351 - 5547698	1	+		hypothetical protein
434	5631188	5634067	ChrXI	TGME49_017230	5635624 - 5635820<br>5636262 - 5636484<br>5636594 - 5636943	5	-		hypothetical protein
435	5974913	5979394	ChrXI	TGME49_016650	5980946 - 5981314<br>5981340 - 5981500<br>5982366 - 5982703	3	-		hypothetical protein, conserved
436	5982374	5985736	ChrXI	TGME49_016640	5980946 - 5981314<br>5981340 - 5981500<br>5982366 - 5982703	3	+		homoserine kinase, putative
437	6099855	6108667	ChrXI	TGME49_016500	6109947 - 6110176	1	-		tRNA synthetase, putative
438	6120192	6122156	ChrXI	TGME49_016470	6124660 - 6124830	1	-		hypothetical protein
439	6125385	6127560	ChrXI	TGME49_016460	6124660 - 6124830	1	+		hypothetical protein
440	6341501	6347493	ChrXI	TGME49_016190	6341110 - 6341357	3	+		hypothetical protein
441	6473977	6485407	ChrXI	TGME49_015990	6470635 - 6470874<br>6471105 - 6471302<br>6473633 - 6473738<br>6473815 - 6473977	4	+		helicase, putative
442	27260	29792	ChrXII	TGME49_100360	30159 - 30314	1	-		mitochondrial carrier domain-containing protein
443	31749	39660	ChrXII	TGME49_100350	30159 - 30314	1	+		molybdopterin cofactor sulfurase, putative
444	123772	130199	ChrXII	TGME49_100220	130034 - 130263	1	-		hypothetical protein
445	300477	304309	ChrXII	TGME49_099820	305399 - 305653<br>305699 - 305864	3	-		hypothetical protein
446	407542	410686	ChrXII	TGME49_107830	410776 - 410988	4	-		hypothetical protein
447	784684	791915	ChrXII	TGME49_019620	794375 - 794785	1	-		hypothetical protein
448	795149	796170	ChrXII	TGME49_019610	794375 - 794785	1	+		hypothetical protein
449	1098304	1099255	ChrXII	TGME49_019110	1099572 - 1099817<br>1099955 - 1100201	4	-		hypothetical protein, conserved
450	1146244	1157172	ChrXII	TGME49_018960	1156996 - 1157306	1	-		hypothetical protein
451	1252646	1257516	ChrXII	TGME49_018810	1251072 - 1251282	1	+		histidyl tRNA synthetase 2
452	1714056	1726196	ChrXII	TGME49_017330	1729487 - 1729838	1	-		hypothetical protein
453	1731213	1734946	ChrXII	TGME49_017340	1729487 - 1729838	1	+		hypothetical protein

454	1848268	1849720	ChrXII	TGME49_017490	1847423 - 1847546	1	+	hypothetical protein
455	1980067	1990059	ChrXII	TGME49_017690	1993779 - 1994018	1	-	hypothetical protein
456	1997873	2003306	ChrXII	TGME49_017700	1993779 - 1994018	1	+	hypothetical protein
457	2048327	2050819	ChrXII	TGME49_017730	2052359 - 2052693	1	-	hypothetical protein
458	2057672	2061404	ChrXII	TGME49_017740	2052359 - 2052693	1	+	oxoacyl-ACP reductase, putative
459	2435798	2441946	ChrXII	TGME49_045660	2441620 - 2441949	1	-	hypothetical protein, conserved
460	2443396	2447710	ChrXII	TGME49_045670	2441620 - 2441949	1	+	pyruvate dehydrogenase, putative
461	2483984	2500508	ChrXII	TGME49_045730	2480927 - 2481176<br>2482847 - 2483123<br>2483389 - 2483630	4	+	phosphatidylinositol-4-phosphate 5-Kinase, putative
462	2562607	2565070	ChrXII	TGME49_045990	2565070 - 2565499	1	-	hypothetical protein
463	2566420	2569266	ChrXII	TGME49_046000	2565070 - 2565499	1	+	hypothetical protein
464	2737316	2739454	ChrXII	TGME49_046220	2741593 - 2741859	1	-	hypothetical protein, conserved
465	2743034	2747070	ChrXII	TGME49_046330	2749002 - 2749104	7	-	CRAL/TRIO domain-containing protein
466	2749745	2756782	ChrXII	TGME49_046340	2749002 - 2749104	7	+	DnaJ domain-containing protein
467	2852061	2853012	ChrXII	TGME49_046590	2850941 - 2851184	2	+	hypothetical protein
468	2943595	2947580	ChrXII	TGME49_046750	2950529 - 2950677	1	-	hypothetical protein
469	2950548	2966587	ChrXII	TGME49_046760	2950529 - 2950677	1	+	hypothetical protein
470	3038065	3054715	ChrXII	TGME49_046980	3054769 - 3055074	1	-	hypothetical protein
471	3057232	3063231	ChrXII	TGME49_046990	3054769 - 3055074	1	+	hypothetical protein
472	3193930	3203380	ChrXII	TGME49_047280	3204193 - 3204586	1	-	hypothetical protein
473	3205456	3214729	ChrXII	TGME49_047290	3204193 - 3204586	1	+	hypothetical protein
474	3280123	3287210	ChrXII	TGME49_047380	3289217 - 3289494<br>3289587 - 3289830	5	-	hypothetical protein
475	3291146	3306446	ChrXII	TGME49_047390	3289217 - 3289494<br>3289587 - 3289830	5	+	ATPase, AAA family domain-containing protein
476	3353708	3356296	ChrXII	TGME49_047490	3352727 - 3353059	1	+	hypothetical protein
477	3542054	3544246	ChrXII	TGME49_047750	3540412 - 3540721	2	+	hypothetical protein
478	3592023	3598216	ChrXII	TGME49_047910	3598497 - 3598770	3	-	hypothetical protein

479	3621860	3622962	ChrXII	TGME49_047960	3623897 - 3624141	1	-	hypothetical protein
480	3626937	3630008	ChrXII	TGME49_047970	3623897 - 3624141	1	+	hypothetical protein
481	3668974	3671744	ChrXII	TGME49_048140	3673166 - 3673387	1	-	hypothetical protein
482	3743943	3747199	ChrXII	TGME49_048250	3748003 - 3748299	1	-	pre-mRNA-splicing factor, putative
483	3748250	3749667	ChrXII	TGME49_048260	3748003 - 3748299	1	+	hypothetical protein
484	3752963	3755536	ChrXII	TGME49_048270	3749670 - 3749953	1	+	zinc finger CCCH type protein, putative
485	3762330	3775305	ChrXII	TGME49_048290	3761778 - 3762097	1	+	WD domain-containing protein
486	3869966	3872218	ChrXII	TGME49_048490	3872357 - 3872592	1	-	hypothetical protein
487	3872374	3876523	ChrXII	TGME49_048500	3872357 - 3872592	1	+	hypothetical protein
488	4178449	4194388	ChrXII	TGME49_048830	4195879 - 4196122	1	-	phospholipase C delta 1
489	4241444	4245616	ChrXII	TGME49_048940	4247949 - 4248198	1	-	zinc knuckle domain-containing protein
490	5263249	5267883	ChrXII	TGME49_050870	5269760 - 5270081	1	-	zinc finger DHHC domain-containing protein
491	5459026	5463668	ChrXII	TGME49_051590	5465436 - 5465836	3	-	protease, putative
492	5467865	5470960	ChrXII	TGME49_051600	5465436 - 5465836	3	+	RNA processing factor, putative
493	5579633	5581733	ChrXII	TGME49_051800	5582314 - 5582603	1	-	hypothetical protein
494	5584385	5585487	ChrXII	TGME49_051810	5587819 - 5587939	1	-	translation initiation factor eIF-5A, putative
495	5623296	5627789	ChrXII	TGME49_051880	5629520 - 5629725	1	-	tyrosyl-tRNA synthetase, putative
496	5698121	5698555	ChrXII	TGME49_078980	5699013 - 5699183	2	-	hypothetical protein, conserved
497	5882694	5885741	ChrXII	TGME49_078760	5887068 - 5887429<br>5887777 - 5887924	2	-	hypothetical protein
498	5891634	5892137	ChrXII	TGME49_078750	5887068 - 5887429<br>5887777 - 5887924	2	+	hypothetical protein
499	5928652	5930500	ChrXII	TGME49_078670	5932676 - 5932993	1	-	ubiquitin-conjugating enzyme E2, putative
500	5934976	5947946	ChrXII	TGME49_078660	5932676 - 5932993	1	+	P-type Ca2+-ATPase, putative
501	6145005	6146216	ChrXII	TGME49_078320	6146368 - 6146532<br>6146755 - 6147157<br>6147470 - 6147717	5	-	hypothetical protein
502	6150877	6151236	ChrXII	TGME49_078300	6151545 - 6151823<br>6152632 - 6152902	3	-	hypothetical protein

503	6212244	6220206	ChrXII	TGME49_078210	6220299 - 6220618<br>6220923 - 6221226	6	-	hypothetical protein
504	6243864	6245051	ChrXII	TGME49_078180	6246337 - 6246719	1	-	hypothetical protein
505	6246724	6247701	ChrXII	TGME49_078170	6248233 - 6248615	1	-	hypothetical protein
506	6248819	6251376	ChrXII	TGME49_078160	6248233 - 6248615	1	+	hypothetical protein
507	6293587	6295014	ChrXII	TGME49_078090	6292151 - 6292444<br>6293128 - 6293347	2	+	hypothetical protein
508	6295668	6297827	ChrXII	TGME49_078080	6295041 - 6295220	1	+	hypothetical protein
509	6441980	6443151	ChrXII	TGME49_077880	6443417 - 6443582<br>6444628 - 6444910	4	-	hypothetical protein
510	6445071	6450102	ChrXII	TGME49_077870	6443417 - 6443582<br>6444628 - 6444910	4	+	hypothetical protein, conserved
511	6469352	6475317	ChrXII	TGME49_077840	6475454 - 6475683	2	-	Ras family domain containing protein
512	6532758	6535725	ChrXII	TGME49_077740	6536233 - 6536518	1	-	hypothetical protein
513	6537212	6538178	ChrXII	TGME49_077730	6536233 - 6536518	1	+	hypothetical protein, conserved
514	6689124	6690700	ChrXII	TGME49_077220	6691522 - 6691827	2	-	hypothetical protein
515	6693175	6695593	ChrXII	TGME49_077210	6691522 - 6691827	2	+	XPG N-terminal domain containing protein
516	6879564	6880400	ChrXII	TGME49_076840	6880249 - 6880455	2	-	hypothetical protein, conserved
